# Supplementary material for: Opportunities and Challenges of In Vitro Tissue Culture Systems in the Era of Crop Genome Editing
Source: Int J Mol Sci. 2023 Jul 25;24(15):11920. doi: 10.3390/ijms241511920 (PMC10419073; doi:10.3390/ijms241511920)
Supplement: Supplementary file 1 [file ijms-24-11920-s001.zip › ijms-2490815-supplementary.pdf]

Table S1. List of genome editing (GE) studies among the nine major crop species using tissue culture. The genotypes, explants, SDNs, delivery systems and gene targets, mutation rates and study references are listed in separate columns. If nothing else is stated under the mutation rates, they are based on the rate of regenerated plants. The list is based on GE studies from January 2011 until June 2022.

**Species**	**Genotype**	**Explant**	**SDN**	**Delivery systems**	**Target gene(s)**	**Mutation rate (%)**	**References**
Barley	Compass, Flinders, Scope, Spartacus, Golden Promise	Anther culture	CRISPR/Cas9	*Agrobacterium*	*HvPDS*	53	[1]
	Golden Promise	Immature embryo	CRISPR/Cas9	*Agrobacterium*	*HvPM19-1, HvPM19-3*	10-23	[2]
	Igri	Microspore	TALENs	*Agrobacterium*	*GFP*	22	[3]
	Golden Promise	Immature embryo	TALENs	*Agrobacterium*	*HvPAPhy_a*	16-31	[4]
	Igri	Immature embryo	TALENs-HDR	Biolistic	*YFP, GFP*	2-3	[5]
	Golden Promise	Immature embryo	CRISPR/Cas9	*Agrobacterium*	*HvCMF7*	nd	[6]
	Golden Promise	Immature embryo	CRISPR/Cas9	*Agrobacterium* Biolistic	*ENGase*	78	[7]
	Golden Promise	Immature embryo	CRISPR/Cas9-HDR	*Agrobacterium*	*HORVU4Hr1G061310*	50-89	[8]
	Golden Promise	Immature embryo	CRISPR/Cas9	*Agrobacterium*	*HvMPK6*	0.14	[9]
	Igri	Microspore	CRISPR/Cas9	*Agrobacterium*	*EIF4E*	nd	[10]
	Igri	Microspore	CRISPR/Cas9	*Agrobacterium*	*PDIL5-1, EIF4E, QSD1*	nd	[11]
	Golden Promise	Immature embryo	CRISPR/Cas9	*Agrobacterium*	*HvCKX1, HvCKX3*	21-88	[12]
	Golden Promise	Immature embryo	CRISPR/Cas9	*Agrobacterium*	*HvCslF6, HvCslH1, HvCslF3, HvCslF9*	4.7-35.3	[13]
	Golden Promise	Immature embryo	CRISPR/Cas9	*Agrobacterium*	*HvHPT, HvHGGT*	50-65	[14]
	Golden Promise	Immature embryo	CRISPR/Cas9	*Agrobacterium*	*HvCOMT1*	42	[15]
	Golden Promise	Immature embryo	CRISPR/Cas9	*Agrobacterium*	*WDV1,WDV2*	nd	[16]
	Golden Promise	Immature embryo	CRISPR/Cas9	*Agrobacterium*	*D hordein*	0.39	[17]
	Golden Promise	Immature embryo	CRISPR/Cas9	*Agrobacterium*	*B-1,3-glucanases*	nd	[18]
	Golden Promise	Immature embryo	CRISPR/Cas9	*Agrobacterium*	*PTST1, GBSS1a*	1.74-3.33	[19]
	Golden Promise	Immature embryo	CRISPR/Cas9	*Agrobacterium*	*HvITPK1*	46	[20]

	Golden Promise	Immature embryo	CRISPR/Cas9	*Agrobacterium*	*EGT2*	nd	[21]
	Golden Promise	Immature embryo	CRISPR/Cas9	*Agrobacterium*	*MORC1, MORC6a*	80-90<br>58	[22]
	Golden Promise	Immature embryo	CRISPR/Cas9	*Agrobacterium*	*HvCKX1*	92.8	[23]
	Golden Promise	Immature embryo	CRISPR/Cas9	Biolistic	*HvMORC1*	70-100	[24]
	Golden Promise	Immature embryo	CRISPR/Cas9	*Agrobacterium*	*HvLFY*	nd	[25]
	Golden Promise	Immature embryo	CRISPR/Cas9	*Agrobacterium*	*HvAP2, HvMADS29*	nd	[26]
	Golden Promise, and 5 commercial barley cultivars	nd	CRISPR/Cas9	nd	*eIF4E*	nd	[27]
	Golden Promise	Immature embryo	CRISPR/Cas9	*Agrobacterium*	*D-hordein*	14-25	[28]
	Golden Promise	Immature embryo	CRISPR/Cas9	*Agrobacterium*	*Qsd1, Qsd2*	30-42.9	[29]
	Golden promise	Immature embryo	CRISPR/Cas9	*Agrobacterium*	*CRT, PDI, IPI, GST, HSP70, HSP26, HSP16.9*	nd	[30]
	Golden Promise, Spartacus, Vlamingh, Compass	Immature embryo	CRISPR/Cas9	*Agrobacterium*	*HvARE1*	50–90	[31]
	Golden promise	Immature embryo	CRISPR/Cas9	*Agrobacterium*	*HvTB2*	nd	[32]
	Golden promise	Immature embryo	CRISPR/Cas9	*Agrobacterium*	*HVP10*	nd	[33]
	Golden promise	Immature embryo	CRISPR/Cas9	*Agrobacterium*	*SSIIa*	5.6-15.4	[34]
	Golden Promise	Embryogenic callus	CRISPR/Cas9	Biolistic	*HvPIN9*	86.7	[35]
	Golden promise	Immature embryo	CRISPR/Cas9<br>TALENs	*Agrobacterium*	*HvPAPhy_a*	44<br>43	[36]
Wheat	Kenong199<br>Bobwhite	Immature embryo	TALENs	Biolistic	*TaMLO-A1, TaMLO-B1, TaMLO-D1*	3.4-6	[37]
	Kenong 199	Immature embryo	CRISPR/Cas9	Biolistic	*TaEDR1*	nd	[38]
	Kenong 199	Immature embryo	CRISPR/Cas9	Biolistic	*TaGW2, TaGASR7*	2.2-4.4	[39]
	BW208,THA53, Don Pedro	Immature scutella	CRISPR/Cas9	Biolistic	*α-gliadin*	5.18	[40]
	Kenong199, Bobwhite	Immature embryo	CRISPR/Cas9	Biolistic	*TaGASR7, TaDEP1, TaNAC2, TaPIN1, TaLOX2, TdGASR7, TaGW2*	1.1-9.5	[41]

	Fielder	Immature embryo	CRISPR/Cas9	*Agrobacterium*	*TaQsd1*	0.88	[42]
	Zhengmai7698	Callus	CRISPR/Cas9	Biolistic	*TaGASR7*	nd	[43]
	Fielder	Immature embryo	CRISPR/Cas9	*Agrobacterium*	*TaPDS*	11-17	[44]
	Bobwhite	Immature embryo	Nickases	Biolistic	*TaMLO*	nd	[45]
	Fielder	Immature embryo	CRISPR/Cas9	*Agrobacterium*	*TaCKX2-1, TaGLW7, TaGW2, TaGW8*	10	[46]
	Fielder	Immature embryo	CRISPR/Cas9	*Agrobacterium*	*TaPDS*	83	[47]
	Kenong199, YZ814	Immature embryo	CRISPR/Cas9 IVTs	Biolistic	*GASR7, LOX2 and CER9*	1.8	[48]
	Fielder	Immature embryo	CRISPR/Cas9	*Agrobacterium*	*PINB, WAXY, DA1*	6.8-54.17	[49]
	Zhengmai, Bobwhite	Immature embryo	CRISPR/Cas9	Biolistic	*TaSBEIIa*	nd	[50]
	Fielder	Immature embryo	CRISPR/Cas9	Biolistic<br>*Agrobacterium*	*TaQsd1*<br>*TraesCS4A02G110300*<br>*TaLOX2*	3-38.5<br>3.8-6.7<br>6.9	[51]
	CB037	Immature embryo	CRISPR/Cas9	Biolistic	*TaNP-A1, TaNP-B1, TaNP-D1*	5.83-6.56	[52]
	Bobwhite	Immature embryo	CRISPR/Cas9 replicons	Biolistic	*TaUbi, MLO*	10-fold	[53]
	Fielder, Gladius	Immature embryo	CRISPR/Cas9	*Agrobacterium*	*Ms1*	70	[54]
	Fielder	Immature embryo	CRISPR/Cas9	*Agrobacterium*	*TaDA1 , TaPDS, TaNCED1*	37.04	[55]
	H29, Fielder Ningchun4	Immature embryo	CRISPR/Cas9	*Agrobacterium*	*TaWaxy, TaMTL*	80.5	[56]
	Bobwhite	Mature embryo SAM	CRISPR/Cas9	Biolistic	*TaGASR7-A1,TaGASR7-B1,TaGASR7-D1*	5.2	[57]
	Svevo	Immature embryo	CRISPR/Cas9	Biolistic	*CM3*<br>*CM16*	13<br>7.4	[58]
	Borlaug 2016	Immature embryo	CRISPR/Cas9	*Agrobacterium*	*TaIPK1.A, TaIPK1.B, TaIPK1.D*	10.8-12.7	[59]
	Fielder	Immature embryo	CRISPR/Cas9	*Agrobacterium*	*pinb-47*<br>*waxy-2*<br>*ppo-7*<br>*psy-13*	6.82-38.72<br>46.73-100<br>6.02-37.27<br>5.81-16.7	[60]

	Cadenza, Cezanne, Goncourt, Prevert	Immature embryo	CRISPR/Cas9	Biolistic	*TaBAK1-2, Ta-eIF4E, Ta-eIF(iso)4E*	nd	[61]
	Cadenza	Immature embryo	CRISPR/Cas9	Biolistic	*TaASN2*	64	[62]
	DMSW	Immature embryo	CRISPR/Cas9	*Agrobacterium*	*Ms2*	9	[63]
	Bobwhite	Immature embryo	CRISPR/Cas9	Biolistic	*TaGW7*	1.1-8.3	[64]
	Bobwhite	Immature embryo	CRISPR/Cas9	Biolistic	*TaGW2, TaLpx-1, TaMLO*	20	[65]
	Fielder	Immature embryo	CRISPR/dCas9	*Agrobacterium*	*TaPDS*	nd	[66]
	Fielder	Immature embryo	CRISPR/dCas9	*Agrobacterium*	*α- gliadin, γ-gliadin*	nd	[67]
	Fielder, SBC0456D	Immature embryo	CRISPR/dCas9	*Agrobacterium*	*Ms45*	nd	[68]
	Fielder	Immature embryo	CRISPR/dCas9	*Agrobacterium*	*TaNFXL1*	nd	[69]
	Reeder	Immature embryo	CRISPR/Cas9<br>CRISPR/AsCpf1<br>CRISPR/LbCpf1	Biolistic	*TaPDS*	nd	[70]
	Desert King, Fielder<br>Hahn, Cadenza	Immature embryo	CRISPR/dCas9	*Agrobacterium*	*AP2L-A5*	97	[71]
	Fielder	Immature embryo	CRISPR/Cas9	*Agrobacterium*	*TaHAG1*	nd	[72]
	Fielder	Immature embryo	CRISPR/Cas9	*Agrobacterium*	*YGL1*	nd	[73]
	Kenong199	Immature embryo	CRISPR/MAD7	Biolistic	*TaDEP1, TaDME-T1*	65.6	[74]
	Fielder	Immature embryo	CRISPR/Cas9	*Agrobacterium*	*TaZIP4-B2*	4.9	[75]
	Yumechikara<br>Haruyokoi<br>Kitanokaori	Embryonic shoot apical meristem (SAM)	CRISPR/Cas9	Biolistic	*TaQsd1*	2.51	[76]
	Fielder	Embryonic shoot apical meristem (SAM)	CRISPR/Cas9	Biolistic	*TaLOX2*<br>*TaGASR7*	8-11	[77]
	Fielder	Immature embryo	CRISPR/Cas9	*Agrobacterium*	*TaPLA*	nd	[78]
	Fielder	Immature embryo	Base editing-ABE	*Agrobacterium*	*Tubulins*	78.3	[79]
	Fielder	Immature embryo	CRISPR/Cas9	*Agrobacterium*	*TaPHT1;9-4B*	nd	[80]
	Kenong199	Immature embryos	Base editing-ABE	Biolistic	*TaDEP1*<br>*TaGW2*	1.1<br>0.4	[81]

	Kenong199	Immature embryo	Base editing-CBE	*Agrobacterium*	*TamiR396 and TaGASR6*	2.0-3.5	[82]
	Kenong199	Immature embryo	TALEN	Biolistic	*TaGW2*	1.77	[83]
	NIL-Ne2, RIL-66	Immature embryo	CRISPR/Cas9	Biolistic	*Ne2*	nd	[84]
	Fielder	Immature embryo	CRISPR/Cas9	*Agrobacterium*	*WFZP-A/D, TaDL-A/B/D*	nd	[85]
	nd	Maize sperm cell	CRISPR/Cas9	Pollination	*TaBRI1, TaSD1*	nd	[86]
	Bobwhite, Zhengmai 7698, Fielder	Seedlings	CRISPR/Cas9	BSMV-Virus	*TaPDS, TaGW2, TaGASR7,*	12.9-100	[87]
	Fielder	Immature embryo	CRISPRa and CRISPRi	*Agrobacterium*	*TaPDS*	nd	[66]
	nd	Mature embryos SAMs	CRISPR/Cas9 RNPs	Biolistic	*TaQsd1, TaOr_t0, TaOr_t1 TaHRGP-like1_t2, TaSD1*	1-8.3	[88]
	CB037B	Immature embryo	CRISPR/Cas9	*Agrobacterium*	*TaPDI*	65.6	[89]
	CB037	Immature embryo	CRISPR/Cas9	*Agrobacterium*	*TaMBF1c*	nd	[90]
	Fielder	Immature embryo	CRISPR/Cas9	*Agrobacterium*	*TaPGS1*	nd	[89]
	CB037	Immature embryo	CRISPR/Cas9	Biolistic	*FT-1*	nd	[91]
	Bobwhite	Immature embryo	CRISPR/Cas9	Biolistic	*TaPDS, TaHRC*	49-58	[92]
	Fielder	Immature embryo	CRISPR/Cas9	*Agrobacterium*	*MTL*	nd	[93]
Rice *Japonica*	Kitaake	Callus	TALENs	*Agrobacterium*	*Os11N3*	T_1 (48/63)	[94]
Japonica	Nipponbare	Callus	TALENs	*Agrobacterium*	*OsDEP1*<br>*OsBADH2*<br>*OsCKX2*<br>*OsSD1*	31.3<br>26.9<br>61.7<br>3.8	[95]
Rice *(Indica)*	Kasalath	Callus	CRISPR/Cas9	*Agrobacterium*	*SPP*<br>*ROC5*<br>*YSA*	5<br>26<br>75	[96]
	Kitaake	Callus	CRISPR/Cas9	*Agrobacterium*	*OsCAO1*<br>*OsLAZY1*	83.3 (T_1)<br>91.6 (T_1)	[97]
	Nipponbare	Callus	CRISPR/Cas9	Biolistics	*OsPDS*<br>*OsBADH2*	9.7<br>7.1	[98]

	Kitaake	Callus	CRISPR/Cas9	*Agrobacterium*	*OsSWEET11*<br>*OsSWEET13*<br>*OsSWEET1a*<br>*OsSWEET1b*<br>*Chr. 2 cluster (245 kb)*<br>*Chr. 4 cluster (170 kb)*	12.5<br>100<br>70<br>20<br>16.6<br>25	[99]
	Nipponbare	Callus	CRISPR/Cas9	Biolistic	*OsPDS*<br>*OsMPK2*	9.4<br>4	[100]
Indica	Nipponbare<br><br>Kasalath	Callus	CRISPR/Cas9	*Agrobacterium*	*OsMYB1*<br>*OsYSA*-gRNA 1<br>*OsROC5*<br>*OsDERF1*<br>*OsYSA* – gRNA 2<br>*OsPDS*<br>*OsMSH1*<br>*OsMYB5*<br>*OsSPP*<br>*OsPMS3*<br>*OsEPSPS*<br>*OsMSH1+OsDERF1*<br>*OsMSH1+OsPDS*<br>*OsPDS+OsPMS3*<br>*OsPDS+OsDERF1*	66.7<br>66.7<br>65.1<br>50.9<br>51.4<br>41.9<br>37<br>31.9<br>28.9<br>26.3<br>21.1<br>33.3<br>32.7<br>8.6<br>5.7	[101]
	Nipponbare	Callus	CRISPR/Cas9	*Agrobacterium*	*BEL*	2-16	[102]
	Nipponbare<br>Kitaake	Callus	TALENs	*Agrobacterium*	*OsSWEET14*	nd	[103]
	nd	Callus	CRISPR/Cas9	*Agrobacterium*	*OsDL*	77.8	[104]
	Nipponbare	Callus	TALENs<br>Base substitution	Biolistic	*OsEPSPS*	21<br>1.2	[105]
	Nipponbare	Callus	CRISPR/Cas9	*Agrobacterium*	*OsPDS*<br>*Os02g23823*<br>*OsMPK2*<br>*OsPDS+Os02g23823+OsMPK2*	68.2<br>66.4<br>81<br>60	[106]
	Kitaake	Callus	CRISPR/Cas9	*Agrobacterium*	*OsSWEET13*	nd	[107]
	Nipponbare	Callus	TALENs<br>TALENs	*Agrobacterium*<br>Biolistic	*OsBADH2*<br>*OsBADH2+ OsCKX2+ OsDEP1*	30<br>1.9	[108]
	Nipponbare	Callus	TALENs	*Agrobacterium*	*OsLOX3*	4	[109]

	Nipponbare	Callus	CRISPR/Cas9	*Agrobacterium*	*OsYSA-g1+OsROC5+OsYSA-g2*	33.3+53.3+46.7	[110]
	Nipponbare	Callus	CRISPR/Cas9	*Agrobacterium*	*OsAOX1a*<br>*OsAOX1b*<br>*OsAOX1c*<br>*OsBEL*	50<br>57<br>75<br>86	[111]
	Taichung 65	Callus	CRISPR/Cas9	*Agrobacterium*	Os 1 (8 FTL targets)<br>Os 2 (3 FTL targets)<br>Os 3 (3 targets)<br>Os 4 (3x Waxy targets)<br>Os 5 (Os05g0591600)<br>Os 6 (Os03g0126800+Os03g0126800)<br>Os 7 (Os07g0409500+Os07g0409500)<br>Os 8 (Os07g0625500+Os07g0625500)<br>Os 9 (Os07g0261200+Os07g0261200)<br>Os 10 (3x targets)<br>Os 11 (3x targets)<br>Os 12<br>(Os07g0411300+Os10g0484800)<br>Os 13 (Os04g0595000)<br>Os 14 (Os12g0242700)<br>Os 15 (Os03g0216800)<br>Os 16<br>(Os02g0459600+Os01g0891000)<br>Os 17<br>(Os10g0413900+Os10g0413900)<br>Os 18<br>(Os06g0142000+Os06g0142100)<br>Os 19 (Os03g0247300)<br>Os 20<br>(Os06g0275000+Os06g0275000)	Average 85.4	[112]
	Kitaake<br><br>Nipponbare	Callus	CRISPR/Cas9<br><br>CRISPR/Cas9	*Agrobacterium*	*sgRNA1*<br>*sgRNA2*<br>*PTG6*<br>*PTG7*<br>*PTG9*	44<br>60<br>100<br>47<br>86	[113]
	Nipponbare	Callus	CRISPR/Cas9	*Agrobacterium*	*PDS*-1<br>*PDS*-2<br>*PDS*-3<br>*PDS*-4	46<br>3<br>93<br>87	[114]

					DL-1<br>*DL*-2<br>*DL*-3<br>*DL*-4<br>*LigIV*-1<br>*LigIV*-2<br>*LigIV*-3<br>*LigIV*-4<br>*ALS*-1<br>*ALS*-2<br>*ALS*-3<br>*ALS*-4	68<br>94<br>93<br>76<br>62<br>90<br>96<br>88<br>5<br>73<br>69<br>26	
	LH422	Callus	CRISPR/Cas9	*Agrobacterium*	*GW2+ TGW6+ GW5*	95 (triple mutant)	[115]
	Nipponbare	Callus	CRISPR/FnCas12a	*Agrobacterium*	*OsDL*<br>*OsALS*	85.7<br>90	[116]
	Kuiku131	Callus	CRISPR/Cas9	*Agrobacterium*	*OsERF922*	42	[117]
	Zhonghua 11	Callus	CRISPR/Cas9	*Agrobacterium*	*OsGn1a*<br>*OsDEP1*<br>*OsGS3*<br>*OsIPA1*	42.5<br>67.5<br>57.5<br>27.5	[118]
	nd	Callus	CRISPR/Cas9	*Agrobacterium*	*OsPDS*<br>*OsYSA*<br>*OsDEP1*<br>*OsPDS (2 sgRNA)*<br>*OsYSA (2 sgRNA)*	85.2<br>100<br>72.2<br>86.2<br>66.7	[119]
	Nipponbare	Callus	TALENs	*Agrobacterium*	*OsCSA*<br>*OsPMS3*<br>*OsDERF1*<br>*OsGN1a*<br>*OsTAD1*<br>*OsMST7*<br>*OsMST8*<br>*OsPMS3*<br>*OsPMS3*	2.3<br>3.8<br>2.8<br>0<br>0<br>6.6<br>5.3<br>25<br>21.7	[120]
	Kitaake	Callus	TALENs-HDR	Biolistic	*OsALS*	6.3	[121]
	nd	Callus	CRISPR/Cas9	*Agrobacterium*	gRNA guides 1-21	Average 82.2	[122]

	Nipponbare/<br>Zhonghua11	Callus	CRISPR/Cas9-VQR<br><br><br><br><br><br><br>CRISPR/Cas9-VRER	*Agrobacterium*	*NAL1*-Q1<br>*NAL1*-Q2<br>*LPA1*<br>*LG1*<br>*GL1-1*<br>*LPA1+LG1*<br>*LG1+GL1-1*<br>*NAL1*<br>*LG1*<br>*GL1-1*	0<br>13.3<br>2.1<br>23.4<br>17<br>2.1<br>8.5<br>3.9<br>1.6<br>36.5	[123]
	Nipponbare	Callus	CRISPR/Cas9<br>CRISPR/Cas9n<br>CRISPR/Cas9<br>CRISPR/Cas9n	*Agrobacterium*	*OsCDKB2*<br><br>*OsDMC1A*	41.6<br>22.7<br>84<br>0	[124]
	Nakdong	Callus	CRISPR/Cas9	*Agrobacterium*	*JAZ9*	69	[125]
	Nipponbare	Callus	CRISPR/Cas9-HDR	Biolistic<br>*Agrobacterium*	*OsALS*	92<br>75	[126]
	Nipponbare	Callus	CRISPR/Cas9-HDR	Biolistic	*OsEPSPS* (gene replacement – 2 targets)<br>*OsEPSPS* (gene insertion – 1 target)	2<br>2.2	[127]
japonica<br>*Indica*<br><br><br><br><br><br><br><br><br><br>*japonica*	Zhonghua11<br>Zhenshan97B<br>ZhongzheB<br>TianfengB<br>YixiangB<br>ReB<br>HuahuiB<br>HuanongB<br>Yuejingsimiao<br>Yuenongsimiao<br>Wushansimiao<br>GAZ	Callus	CRISPR/Cas9	*Agrobacterium*	*TMS5a-j* (paired)<br>*TMS5ab*	69.2-94.1<br>88.89<br>83.33<br>92.86<br>100<br>88.23<br>84.62<br>72.72<br>100<br>11.11<br>85.71<br>92.86	[128]
Japonica<br>*Japonica*<br>*Indica*<br>*Hybrid*<br>*Japonica*	Nipponbare<br>Koshihikari<br>IR64[129]<br>NERICA1<br>Curinga	immature embryos	CRISPR/Cas9	*Agrobacterium*	*OsPDS*	84.9<br>64.7<br>100<br>90.7<br>79.8	[130]
	Xiushui11	Callus	CRISPR/Cas9	*Agrobacterium*	*OsTCD10*	nd	[131]

	Nipponbare	Callus	CRISPR/Cas9	Biolistic	*OsBEIIb*	5	[132]
Japonica	9522<br>Kongyu131<br>Jiaoyou5B	Callus	CRISPR/Cas9	*Agrobacterium*	*CSA*	50<br>6.8<br>69.2	[133]
	Kitaake	Callus	Base editing-CBE	*Agrobacterium*	*OsSERK1*<br>*OsSERK2*<br>*ipa1*<br>*pi-ta*	17<br>10.5<br>22.2<br>18.2	[134]
	Nipponbare	Callus	CRISPR/LbCas12a	*Agrobacterium*	*OsPDS*<br>*OsDEP1*<br>*OsROC5*	100<br>100<br>100	[135]
	Zhonghua11	callus	Base editing-CBE	*Agrobacterium*	*OsNRT1.1B*<br>*OsSLR1*	2.7<br>13.3	[136]
	nd	Embryonic cells	CRISPR/Cas9	*Agrobacterium*	*OsSAPK2*	10	[137]
	Kitaake	Callus	CRISPR/Cas9	*Agrobacterium*	*OsSBEI*<br>*OsSBEIIb*	80<br>70	[138]
Indica	Huazhan<br>Longke638S	Callus	CRISPR/Cas9	*Agrobacterium*	*OsNramp5*	82.4<br>80	[139]
	Nipponbare	Immature emrbyos or callus	TALENs	*Agrobacterium*	*Os09g29100*	4.1 (T_1)	[140]
Japonica	Longdao16<br>Longdao18<br>Daohuaxiang2<br>Songjing19<br>Dongnong430	Callus	CRISPR/Cas9	*Agrobacterium*	*OsHD2-1+OsHD4-1+OsHD4-2+OsHD5+OsHD2-2*	77.8 (sum of all cultivars)	[141]
	Taipei309	Callus	CRISPR/Cas9	*Agrobacterium*	*OsAnn3*	31.6	[142]
	Nipponbare	Callus	Base editing-CBE	*Agrobacterium*	*OsALS*	3.41	[143]
	Kitaake	Callus	TALENs	*Agrobacterium*	*SWEET14*-AvrXa7<br>*SWEET14*- TalC	51<br>30	[144]
	Kitaake	Callus	CRISPR/Cas9	*Agrobacterium*	*MPK1*<br>*MPK2*<br>*MPK6*<br>*MPK5*<br>*MPK5+1*<br>*MPK6+2*<br>*MPK5+1+6+2*	100<br>100<br>100<br>100<br>89<br>67<br>86	[145]

	Xidao #1<br>nd	nd	CRISPR/Cas9	*Agrobacterium*	*OsLCT1*<br>*OsBADH2*	100<br>100	[146]
	Nipponbare	Mature embryo	CRISPR/Cas12a	*Agrobacterium*	*OsPDS*<br>*OsBEL*	21.4, 41.2	[147]
	Nipponbare	Callus	Base editing-CBE	*Agrobacterium*	*OsCDC48*	43.48	[148]
	Kitaake	Callus	Base editing-CBE	*Agrobacterium*	*OsPDS*<br>*OsSBEIIb*	2.3<br>20	[149]
	Kitaake	Callus	CRISPR/FnCas12a-HDR<br>CRISPR/LbCas12a-HDR<br><br>CRISPR/FnCas12a<br>CRISPR/LbCas12a	Biolistic	*CAO1* site #1<br>*CAO1* site #1<br>*CAO1* site #2<br>*CAO1* site #1<br>*CAO1* site #1<br>*CAO1* site #2	8<br>0<br>3<br>24<br>1<br>10	[150]
	nd	Callus	CRISPR/Cas9	*Agrobacterium*	*TMS10L*	nd	[151]
Indica	IR64	Immature embryos	CRISPR/Cas9	*Agrobacterium*	*OsEPFL9*	4-10	[152]
	Nipponbare	Callus	CRISPR/Cas9	*Agrobacterium*	*OsMIR408*<br>*OsMIR528*<br>*OsMIR815(a/b/c)*<br>*OsMIR820(a/b/c)*	47.6<br>88.9<br>10<br>61.1	[153]
	Nipponbare	Callus	CRISPR/FnCas12a<br><br><br><br><br><br><br>CRISPR/LbCas12a	*Agrobacterium*	*OsRLK* (4 sites)<br>*OsEPSPS-1*<br>*OsEPSPS-2*<br>*OsBEL-1*<br>*OsBEL-2*<br>*OsPDS-1*<br>*OsPDS-2*<br>*OsBEL* (4 sites)<br>*OsEPSPS-1*<br>*OsEPSPS-2*<br>*OsBEL-1*<br>*OsBEL-2*<br>*OsPDS-1*<br>*OsPDS-2*	34.4<br>0<br>16.7<br>19.4<br>12.5<br>8.3<br>0<br>40<br>5.6<br>20.8<br>62.5<br>20<br>73.5<br>1.8	[154]
	Nipponbare	Callus	CRISPR/Cas9	*Agrobacterium*	*BADH2+DEP1+Gn1a+GS3+GW2*<br>*+Hd1+EP3+LPA1*	25	[155]
	Nipponbare	Callus	CRISPR/SpCas9-HDR	*Agrobacterium*	*OsALS*	2.1	[156]

	Nipponbare	Callus	CRISPR/Cas9	*Agrobacterium*	*OsCYP97A4*<br>*OsDSM2*<br>*OsCCD4a*<br>*OsCCD4b*<br>*OsCCD7*	37.5<br>50<br>37.5<br>93.7<br>20	[157]
	Zhonghua 11	Callus	CRISPR/Cas9	*Agrobacterium*	Library (12,802 genes)	Nd	[158]
Japonica	Kuiku 131	Callus	TALENs	*Agrobacterium*	*OsERF922*	2.7	[159]
	E4	Callus	CRISPR/Cas9	*Agrobacterium*	*SaF*<br>*SaM*	Nd<br>nd	[160]
	Nipponbare	Callus	CRISPR/Cas9<br><br><br><br><br><br><br>CRISPR/Cas12a	*Agrobacterium*	*OsDEP1*<br>*OsSPL14*<br>*OsTB1*<br>*OsLAC*<br>*OsmiR398a*<br>*Os02circ25329* (2x)<br>*Os03circ00204* (2x)<br>*OsPDS*<br>*OsDEP1*<br>*OsROC5*	75<br>63.6<br>15.4<br>58.8-100<br>71.4<br>66.7-80<br>18.8-43.8<br>100<br>100<br>100	[161]
	Nipponbare	Immature embryo or callus	CRISPR/Cas9	*Agrobacterium*	*RAD51A*-NGG<br>*RAD51A*-NAG<br>*DMC1*-NGG<br>*DMC1*-NAG<br>*NAL1+LPA1+LG1+GL1-1* (NAG)<br>*NAL1+ LPA1+LG1+LG1* (NAG)	87.5<br>67.9<br>74.3<br>34.3<br>15.5-44.8<br>21.4+82.1	[162]
Japonica	Nanjing 9108<br>Wuyunjing 27<br>Yangjing 4227<br>Zhejing 22<br>Zhejing 88	Callus	CRISPR/Cas9	*Agrobacterium*	*OsGS3+OsGn1a*	nd	[163]
	Zhonghua11	Callus	CRISPR/Cas9	*Agrobacterium*	*OsPDS (multiplex)*	50-85	[164]
	Kitaake	Callus	CRISPR/LbCas12a-RR	*Agrobacterium*	*OsPDS*<br>*OsSBEIIb*<br>*OsPDS+OsSBEIIb*	22.2<br>31.1<br>9.3	[165]
	Nipponbare	Callus	CRISPR/Cas9<br><br><br>CRISPR/Cas9-VQR	*Agrobacterium*	*OsMOC3*<br>*OsGW2*<br>*OsMOC3+OsGW2*<br>*3x targets*	65.9<br>56.5<br>29.35	[166]

						13.9 (double mutant)<br>Approx.. 5<br>Approx. 10	
	Nipponbare	Callus	CRISPR/Cas9	*Agrobacterium*	*OsPDS*<br>*OsGn1a*<br>*OsDEP1*	28<br>49<br>39	[167]
	Zhonghua11	Callus	CRISPR/LbCas12a-HDR	biolistic	*ALS*-gene replacement	2.1-2.8	[168]
	Zhonghua11	Callus	Base editing-CBE	*Agrobacterium*	*OsCDC48*<br>*OsNRT1.1B-T1*	82.9<br>44.1	[169]
	Dongjin	Germinating rice seed	ZFN	*Agrobacterium*	*SSIVa*	57.1	[170]
	Nipponbare	Callus	Base editing-CBE	*Agrobacterium*	*OsALS*<br>*OsALS+OsFTIP1e*	64.1<br>43.2	[171]
	Zhonghua11	Callus	Base editing-ABE	*Agrobacterium*	*OsACC*<br>*OsALS*<br>*OsCDC48*<br>*OsDEP1-T1*<br>*OsDEP1-T2*<br>*OsNRT.1B*	20.6<br>21.4<br>33.3<br>46.5<br>15.8<br>59.1	[81]
	Nipponbare	Immature embryo or callus	CRISPR/Cas9	*Agrobacterium*	*CCD7*	36.4-100	[172]
	Nipponbare	Callus	CRISPR/FnCas12a<br><br><br><br><br>CRISPR/LbCas12a-RR<br><br><br><br><br>CRISPR/FnCas12a-RR<br><br>CRISPR/LbCas12a-RVR<br><br>CRISPR/FnCas12a-RVR	*Agrobacterium*	*OsDEP1*-TTV<br>*OsEPFL9*-TTV<br>*OsDPS*-TTV<br>*OsDEP1*-TTTV<br>*OsEPFL9*-TTTV<br>*OsROC5*-CCCC<br>*OsPDS*-TCCG<br>*OsROC5*-TCCG<br>*OsPDS*-TTCC<br>*OsROC5*-TTCC<br>*OsDEP1*-CCCC<br>*OsDEP1*-TTCC<br>*OsPDS*-TATC<br>*OsROC5*-TATG<br>*OsDEP1*-TATG	46.7<br>57.1<br>0<br>87.1<br>89.3<br>47.5<br>0<br>40<br>93.3<br>100<br>0<br>13.3<br>0<br>60<br>0	[173]
	Nipponbare	Callus	CRISPR/FnCas12a	*Agrobacterium*	*LEA_1+LEA_2* (8 targets)	0-70.8<br>0-70.8	[174]

			CRISPR/LbCas12a<br><br>CRISPR/Cas9		*LEA_Dehydrins* (9 targets)<br><br>*CYP81A* (3 targets)	4.2-54.2<br>4.2-58.3<br>70.8-79.2<br>50-86.4	
	Kitaake	Callus	Base editing-CBE	*Agrobacterium*	*Pi-d2*<br>*OsFLS2*<br>*OsAOS1*<br>*OsJAR1*<br>*OsJAR2*<br>*OsCOI2*	30.8<br>57<br>23.3<br>21.7<br>11.8<br>69.4	[175]
	Nipponbare	Callus	TALENs	*Agrobacterium*	*Os01g0833500*	16.8-60.4	[176]
	Nipponbare	Callus	CRISPR/Cas9	*Agrobacterium*	*PYL1–PYL6+ PYL12*<br>*PYL7–PYL11+PYL13*	nd	[177]
	Nipponbare	Scutellum	CRISPR/Cas9	*Agrobacterium*	*OsFAD2-1*	8.7	[178]
	Nipponbare	Callus	Base editing-ABE	*Agrobacterium*	*OsSPL14*<br>*SLR1*<br>*OsSPL16+OsSPL18*<br>*OsSPL14+OsSPL17*<br>*OsSPL16+OsSPL18*	26<br>12.5<br>9.5<br>41.9<br>14.6	[179]
	Kitaake	Callus	Base editing-ABE	*Agrobacterium*	*OsSERK2*<br>*OsWRKY45*	32<br>62.3	[180]
Indica	IR64	Immature embryos	CRISPR/Cas9	*Agrobacterium*	*eIF4G*	59.7	[181]
Indica	ASD16	Immature embryos	CRISPR/Cas9	*Agrobacterium*	*OsPFT1*	nd	[182]
	Kitaake	Callus	CRISPR/Cas9	*Agrobacterium*	*OsBBM1*<br>*OsBBM2*<br>*OsBBM3*	Nd<br>Nd<br>Nd	[183]
	IR64	Immature embryos	CRISPR/Cas12a	*Agrobacterium*	*OsEPFL9*	nd	[184]
Rice hybrid	Chunyou 84	nd	CRISPR/Cas9	*Agrobacterium*	*OsREC8+OsPAIR1+OsOSD1*<br>*OsMTL*<br>*OsREC8+OsPAIR1+OsOSD1+OsMTL*	21.9<br>34.4<br>13.6	[185]
	Nipponbare	Callus	CRISPR/Cas-directed evolution (CDE)	*Agrobacterium*	*OsSF3B1*	0.14	[186]
	nd	Callus	CRISPR/Cas9	*Agrobacterium*	*OsMYB108*	14.3-100	[187]
	nd	nd	Base editing-ABE	nd	*Wx*<br>*GL2/OsGRF4*	15.1<br>13.3	[188]

					OsGRF3	16.7	
	Kitaake	Callus	CRISPR/Cas9	*Agrobacterium*	*OsSWEET11+OsSWEET13+OsSWEET14*	25	[189]
indica	IR64					Nd	
	Ciherang-Sub1					Nd	
	Xidao 1	Callus	CRISPR/Cas9	*Agrobacterium*	*OsITPK6*	30.4	[190]
	Nipponbare	Callus	CRISPR/Cas9	*Agrobacterium*	*OsPDS*	20.7	[191]
					OsDEP1	52.9	
					OsPDS	77.1	
					OsDEP1	86.6	
					OsPDS	75.9	
					OsDEP1	93.3	
	Nipponbare	Callus	CRISPR/xCas9	*Agrobacterium*	*OsGS3*-CGA	11.1	[192]
					DEP1-CGT	6.7	
					DEP1-TGC	21.1	
					DEP1-CGC	0	
			CRISPR/Cas9-NG		*DEP1*-CGC	56.3	
			CRISPR/Cas9-NGv1			18.2	
			CRISPR/Cas9-NG		*PDS*-AGG	33.3	
			CRISPR/Cas9-NGv1			8	
			CRISPR/Cas9-NG		*PDS*-CGT	22.2	
			CRISPR/Cas9-NGv1			3.5	
			CRISPR/Cas9-NG		*DEP1*-GGT	38.5	
			CRISPR/Cas9-NGv1			13.6	
			Cas9-NG(base editing)		*OsDEP1* site 1	45	
					OsDEP1 site 2	30.4	
	Kitaake	Callus	CRISPR/Cas9-NG	*Agrobacterium*	*OsMPK11*	38.2	[193]
					OsMPK7	25	
					OsMPK10	21.4	
					OsMPK8	4.5	
					OsSERK1+OsSERK2	93.3	
			Base editing-CBE		*OsGSK4+OsETR2*	12.8	
			Base editing-ABE		*OsBZR1*	35.3	
					OsSERK2	54.2	
					OsSERK2	40.4	
					OsSERK2	upregulation	

	Nipponbare	Callus	Base editing-ABE	*Agrobacterium*	*OsSPL14+OsSPL17*	66.7+74.3	[194]
					OsSPL16+OsSPL18	30+66.7	
					SNB	6.5	
			Base editing-CBE	*Agrobacterium*	*PMS3*	71.4	
			CRISPR/pSaKKH-Cas9	Bombardment	*PMS1*	35.7	
			Base editing-ABE+CBE	Bombardment	*OsSPL14+OsSPL17+SNB*	9.8	
	Nipponbare	Callus	CRISPR/Cas9	*Agrobacterium*	*OsPDS*	74.5	[195]
						88.2	
						81	
					OsPDS+OsDEP1	76.9	
						90.6	
						86.7	
					OsPDS+OsPDS-sgRNA02+*OsDEP1*	58.3	
						60.6	
						67.6	
					OsPDS-sgRNA01+*OsPDS*-sgRNA02+*OsDEP1*-sgRNA01+*OsDEP1*-sgRNA02+*OsYSA*-sgRNA01+*OsYSA*-sgRNA02 (6x)	47.4	
					OsDEP1-crRNA01		
			CRISPR/Cas12a		*OsDEP1*-crRNA02	61.1	
					OsROC5-crRNA01	82.4	
					OsROC5-crRNA02	53.8	
					OsDEP1-crRNA01+*OsDEP1*-crRNA02+*OsROC5*-crRNA01+*OsROC5*-crRNA02	54.5<br>16.7	
	Nipponbare	Callus	CRISPR/Cas9	*Agrobacterium*	*OsIAMT*	nd	[196]
	Nipponbare	Callus	CRISPR/Cas9	*Agrobacterium*	*OsCAld5H1*	0-100	[197]
	Nipponbare	Callus	CRISPR/Cas9	*Agrobacterium*	*Tos17*^{chr7}	10-90	[198]
	nd	nd	CRISPR/SaCas9	*Agrobacterium*	*PDS*	64.2	[199]
					DL	77.8	
			CRISPR/SaKKH		*PDS*	90.6	
					DL	41.7	
					NAL1	66.7	
			CRISPR/Sa-BE3 (CBE)		*SLR1*	10	
			CRISPR/Sa-eBE3 (CBE)			71.9	
			CRISPR/Sa-BE3 (CBE)		*IPA1*	8.3	
			CRISPR/Sa-eBE3 (CBE)			15	

			CRISPR/Sa-BE3 (CBE)<br>CRISPR/SaKKH-BE3 (CBE)<br><br>CRISPR/SaKKH-eBE3 (CBE)<br><br>CRISPR/Sa-CDA (CBE)<br><br>CRISPR/SaKKH-CDA (CBE)<br><br>CRISPR/Sa-ABE<br><br>CRISPR/SaKKH-ABE		*TAC1*<br>*Ehd1*<br>*Pi-d2*<br>*Ehd1*<br>*Pi-d2*<br>*IPA1*<br>*OsMKK6*<br>*OsMPK3*<br>*Pi-ta*<br>*IPA1*<br>*OsSPL17*<br>*GL2*<br>*OsGRF*<br>*Wx*<br>*Pi-d3*	15.6<br>20.5<br>6.3<br>25<br>5.4<br>8.5<br>2.1<br>6.3<br>0<br>41.2<br>63.2<br>6.5<br>16.1<br>9.4<br>0	
	WPB106	Callus	CRISPR/Cas9	*Agrobacterium*	*OsRR22*	64.3	[200]
Indica	93-11<br>Huazhan	Callus	CRISPR/Cas9	*Agrobacterium*	*PTGMS2-1*	nd	[201]
	Nipponbare	Callus	CRISPR/Cas9	*Agrobacterium*	*OsOr*	nd	[202]
	Nipponbare	Callus	CRISPR/Cas9<br>CRISPR/xCas9 3.6<br>CRISPR/xCas9 3.7<br>CRISPR/SpCas9-NG<br>CRISPR/xCas9 3.6<br>CRISPR/xCas9 3.7<br>CRISPR/SpCas9-NG<br>CRISPR/xCas9 3.6<br>CRISPR/xCas9 3.7<br>CRISPR/SpCas9-NG<br>CRISPR/xCas9 3.6<br>CRISPR/xCas9 3.7<br>CRISPR/xCas9 3.6<br>CRISPR/xCas9 3.7<br>Base editing-ABE	*Agrobacterium*	*OsSPL14*-CGG<br><br><br><br>*LF*-AGC<br><br><br>*OsSPL7*-CGT<br><br><br>*OsSPL4*-GAT<br><br>*OsMADS57*-GAA<br><br>*OsSPL14*<br><br><br><br>*LF1*	87.9<br>85.3<br>89.3<br>65.7<br>23.8<br>3.2<br>81.8<br>84<br>85.7<br>74.2<br>11.1<br>3.3<br>0<br>0<br>0<br>0<br>2.6<br>2.9<br>0	[203]

			Base editing-CBE			0<br>2<br>7.7	
					OsIAA13	0<br>0<br>11.9	
					OsSPL7	4.8<br>0<br>2.9	
					OsSPL4	0<br>0	
					OsMADS57	0<br>0	
					SNB	17.3<br>35.7<br>72.2<br>37.5	
					OsSPL7	0<br>0<br>27.2	
					PMS3	0<br>0<br>4.5	
					OsSPL14	0<br>0<br>0<br>0	
	Nipponbare	nd	CRISPR/LbCas12a	nd	*OsPDS*	21.4<br>39.6<br>43.8<br>77.8<br>71.2	[204]
					OsGS3	43.7<br>50<br>62.5<br>91.7<br>62.5	
					ALS	70	

	Zhonghua11		CRISPR/LbCas12a-RR<br>CRISPR/LbCas12a-RVR		*NAL*<br>*Gn1a+GS3+IPA1*<br>*LCD+OsNrAMP5+LCT1+OsNrAMP1*<br>*OsDL*-CCCC<br>*OsPDS*-TTCA<br>*OsDL*-TATC<br>*OsPDS*-TATG	92.9<br>35.7<br>66.7<br>17.8<br>56.3<br>41.7<br>69.4<br>8.3<br>45<br>2<br>13.9	
	Zhonghua 11	Callus	CRISPR/Cas9	*Agrobacterium*	*OsSPCH1*<br>*OsSPCH2*<br>*OsMUTE*<br>*OsFAMA*<br>*OsICE1*<br>*OsICE2*<br>*OsSCR1*<br>*OsSCR2*<br>*OsSHR1*<br>*OsSHR2*	nd	[205]
	Xidao 1	Callus	CRISPR/Cas9	*Agrobacterium*	*OsLCT1*<br>*OsNramp5*	21.7<br>16.7-100	[206]
	nd	Callus	CRISPR/xCas9<br>CRISPR/Cas9<br>CRISPR/xCas9<br>CRISPR/Cas9<br>CRISPR/xCas9<br>CRISPR/Cas9	*Agrobacterium*	GAA PAM sites<br>AGA, TGT and CGC PAM sites<br>GGG PAM site	0-4.2<br>2.1-12.5<br>0<br>2.1-8<br>4.2-18.4<br>0<br>4.2-10.4<br>75-77.1	[207]
	Pinzhan	Callus	CRISPR/Cas9	*Agrobacterium*	*TMS5+Pi21+Xa13*	nd	[208]
	Nipponbare	nd	CRISPR/Cas9	*Agrobacterium*	*OsLHT1*	nd	[209]
Japonica	Sasanishiki	Callus	CRISPR/Cas9	*Agrobacterium*	*Se13*<br>*PHYB*<br>*Se14*	60<br>45<br>35	[210]

					Hd3a<br>*Ef7*<br>*RFT1*<br>*Ehd1*<br>*Hd1*<br>*Ghd7*<br>*Dth8*	40<br>50<br>55<br>45<br>40<br>70<br>60<br>50	
	Zhonghua 11	Callus	CRISPR/Cas9	*Agrobacterium*	*LAZY1*	44.5-52.5	[211]
	Nipponbare	Callus	CRISPR/AsCas12a	*Agrobacterium*	*OsDEP1*<br>*OsROC5*	77.8<br>92.8	[212]
landraces	Kasalath<br><br>TTP (TeTePu)	Callus	CRISPR/Cas9	*Agrobacterium*	*SD1*<br><br>*SD1+SE*5	44-68<br><br>21-28	[213]
	BTA	Callus	mitoTALENs	*Agrobacterium*	*orf79*	33.3	[214]
	nd	Callus	Base editing-ABE	*Agrobacterium*	chr04_33074491–33074510	30.3	[215]
japonica	Nipponbare and Yukihikari	Zygotes	CRISPR/Cas9-RNP<br><br><br><br><br>CRISPR/Cas9	PEG	gDsRed2<br>gDL-2<br>gDL-3<br>gGW7<br>gGCS1<br>gDL-2<br>gPRR37-1<br>gPRR37-2	25<br>14.3<br>13.6<br>21.4<br>64.3<br>16.7<br>16<br>4	[216]
	Zhonghua 11	Callus	CRISPR/LbCpf1-HDR	Biolistics	*OsALS*	4.6	[217]
	Nipponbare	Callus	CRISPR/Cas9	*Agrobacterium*	Multiple targets (open- and closed chromatin regions)	Up to 100	[218]
	EYI	Mature embryos	CRISPR/Cas9<br>CRISPR/Cas9d	biolistics	*Wx*	nd	[219]
	Nipponbare	Callus	CRISPR/Cas9	*Agrobacterium*	*OsACS1*<br>*OsACS2*	nd	[220]
Indica	Jingxi Glutinous Rice	Callus	CRISPR/Cas9	*Agrobacterium*	*OsGA20ox2*	73.5	[221]
	Kitaake	Callus	CRISPR/Cas9	*Agrobacterium*	*Os8N3*	25	[222]
	Nipponbare	Mature seeds derived embryos	CRISPR/Cas9-RNP<br>CRISPR/Cas9	Biolistics	*OsPDS1*	62.8<br>62	[223]

		Callus	CRISPR/Cas9	*Agrobacterium*		100	
	GXU16<br>GXU20<br>GXU28	Callus	CRISPR/Cas9	*Agrobacterium*	*SRL1 or SRL2*	68.1<br>56.8<br>71.6	[224]
Indica	209B	Callus	CRISPR/Cas9	*Agrobacterium*	*OsWaxy*	68.2	[225]
Japonica	Jijing809<br>Liaojing237	Callus	CRISPR/Cas9	*Agrobacterium*	*OsGS3+OsGW2+OsGN1a*	80<br>33.3	[226]
	Nipponbare	nd	CRISPR/Cas9	*Agrobacterium*	LOC_Os09g26999	nd	[227]
	Dongjin	Callus	CRISPR/Cas9	*Agrobacterium*	*OsVP1*	60	[228]
	Heugseonchal<br><br>Sinmyungheugchal	Callus	CRISPR/Cas9	*Agrobacterium*	*OsF3'H*<br>*OsDFR*<br>*OsLDOX*	60<br>52.9<br>57.1	[229]
	Zhonghua 11	Callus	CRISPR/Cas9	*Agrobacterium*	*OsBIG*	nd	[230]
	Zhonghua 11	Callus	CRISPR/ScCas9	*Agrobacterium*	NAG (PAM)<br>NTG (PAM)<br>NCG (PAM)<br>NGG (PAM)	41.67<br>79.19<br>4.65<br>32.56	[231]
	Nipponbare	Callus	Base editor-CBE	*Agrobacterium*	*ALS-T1*<br>*CHL9*<br>*IPA*<br>*NRT1.1*<br>*SLR1*<br>*ALS-T2*<br>*Pi-d2*	Approx. 66<br>Approx. 71<br>Approx. 61<br>Approx. 52<br>Approx. 89<br>Approx. 75<br>Approx. 86	[232]
	Zhonghua11	Callus	CRISPR/Cas9-HDR	Biolistic	*DRO1*-dsADHE<br>*DRO1*-ssADHE<br>*DRO1*-ADHE<br>*SKC1*-ADHE<br>*SLR1*-ADHE<br>*SOS1*-ADHE<br>*WRKY71*-AMVE<br>*DEP1*-AMVE<br>*BZR1*-AMVE<br>*bZIP5*-AMVE<br><br>*OsPAP10a*- P1BS	4.2<br>0<br>45.5<br>31.7<br>26.5<br>35.7<br>25<br>43<br>47.3<br>23.5<br><br>26.1	[233]

			tandem repeat-HDR strategy (TR-HDR)		*OsPAP10c*- P1BS<br>*OsPAP21b*- P1BS<br>*AVP1*- P1BS<br>*SLR1*- CmYLCV9.11<br>*SKC1*- CmYLCV9.11<br>*SLR1*- UBI<br>*SKC1*- UBI<br>*SLR1*-BS<br>*TT1*-BS<br>*NRT1.1b*-BS<br>*TT1*-flag<br>*UBQ6*-flag	18.1<br>10.6<br>31.4<br>9.1<br>16.4<br>3.9<br>6.6<br>3.7<br>3.4<br>5.4<br>6.4<br>11.4	
	Dongjin	Callus	CRISPR/Cas9	*Agrobacterium*	*SLR1*	51.6	[234]
	Nipponbare	Callus or immature embryos	CRISPR/FnCas12a	*Agrobacterium*	*OsRLK*-798+*OsRLK*-802+*OsALS*<br><br><br><br><br><br>*OsRLK*-798+*OsRLK*-802+*OsALS*<br>*OsLG1-N1+ OsLG1*-N2+*OsNAL1*-N3+*OsNAL1-N4*<br>*OsRLK*-798+*OsRLK*-802+*OsALS*<br>*OsLG1-N1+ OsLG1*-N2+*OsNAL1*-N3+*OsNAL1-N4*	32.2<br><br>20.2<br><br>1.8<br><br>32.2<br>3.7 (double mutation)<br>16<br>9.3 (double mutation)	[235]
	Nipponbare	Callus	Base editor-CBE	*Agrobacterium*	*HIS1* (16-47 gRNA)<br>*HIS1* (16-49 gRNA)<br>*HIS1* (16-81 gRNA)<br>*HIS1* (16-82 gRNA)	3.6<br>13.5<br>13.8<br>21.2	[236]
O. Sativa	Kabre	Callus	CRISPR/Cas9-de *novo domestication*	*Agrobacterium*	*HTD1*<br>*GN1A+ GS3+ GW2*	Nd<br>21	[237]
Japonica	Nangeng 9108 and BL3045	Callus	CRISPR/Cas9-HDR	Biolistic	*pi-ta* → *Pi-ta*	3.8	[238]
	Nipponbare	nd	CRISPR/Cas9	*Agrobacterium*	*MIR396abc*<br>*MIR396d*<br>*MIR396ef*<br>*MIR396g*<br>*MIR396h*<br>*MIR396e*	nd	[239]

					OsGIF1		
	Kitaake	Callus	CRISPR/ScCas9<br><br><br><br><br><br><br><br><br><br><br><br><br><br>Base editor-CBE<br>Base editor-ABE	*Agrobacterium*	*OsMPK9*<br>*OsMPK17-1*<br>*OsCPK6-1*<br>*OsMPK15-1*<br>*OsMPK16*<br>*OsCPK7*<br>*OsMPK17-2*<br>*OsCPK5*<br>*OsCPK6-2*<br>*OsMPK9*<br>*OsMPK15-2*<br>*OsCPK8*<br>*OsMPK14+ OsMPK15*<br>*OsCPK9+ OsCPK10*<br>*OsBZR1*<br>*OsGS1*	0<br>0<br>97.2<br>0<br>91<br>95<br>0<br>0<br>47<br>7<br>0<br>0<br>82+50<br>94+89<br>37<br>47.5	[240]
	Nipponbare	Callus	CRISPR/Cas9	*Agrobacterium*	*OsPDS*<br><br><br><br>*OsDEP1*	75<br>80<br>88.9<br>87.5<br>68.8<br>82.4<br>88.2<br>83.3	[241]
	Zhonghua11	Callus	CRISPR/Cas9	*Agrobacterium*	*OsCCD8*<br>*OsDWARF14*<br>*OsRCD1*-T1+*OsRCD1*-T2	68.5<br>79.7<br>39.1	[242]
	Nipponbare	Rice embryos	CRISPR/Cas9<br>CRISPR/HiFi-SpCas9<br>CRISPR/SpCas9 D10A<br>CRISPR/AsCas12a<br>CRISPR/LbCas12a	RNP-complex-biolistic	*OsPDS*	3.6<br>8.8<br>0<br>0<br>32.3	[243]
	nd	Callus	Prime editor	*Agrobacterium*	*Inactive EGFP*<br><br>*OsALS*	15.6<br>17.1<br>9.1	[244]
	Nipponbare	Callus	Prime editor	*Agrobacterium*	*OsALS*-T1 pegRNA-01<br><br>*OsALS*-T2 pegRNA-03	1.1<br>3.5<br>1.1	[245]

						2.6	
					OsACC-T1 pegRNA-04	0	
						1.7	
					OsACC-T2 pegRNA-05	1.4	
						0	
					OsALS-T1 pegRNA-06	1.2	
						26	
					OsDEP1 pegRNA-07	0	
						9.1	
	Zhonghua11	Callus	CRISPR/SWISSv3 (CBE+ABE+indel)	*Agrobacterium*	*OsALS+ OsACC+ OsBADH2*	7.3	[246]
	Nipponbare	Callus	CRISPR/Cas9	*Agrobacterium*	*OsDEP1*-sgRNA01	90	[247]
					OsDEP1-sgRNA02	46.5	
					OsPDS-sgRNA01	60	
					OsPDS-sgRNA02	53.8	
					OsDEP1-sgRNA01	71.4	
					OsDEP1-sgRNA02	33.3	
					OsPDS-sgRNA01	84.6	
					OsPDS-sgRNA02	10	
					OsDEP1-sgRNA01	44	
					OsDEP1-sgRNA02	33.3	
					OsPDS-sgRNA01	27.3	
					OsPDS-sgRNA02	29.7	
					OsDEP1-sgRNA01+*OsDEP1*-sgRNA02	58.6+10	
					OsDEP1-sgRNA01+*OsPDS*-sgRNA02	71.4+4	
			CRISPR/Cas12a		*OsDEP1*-crRNA01	46.7	
					OsROC5-crRNA-01	9.1	
					OsDEP1-crRNA01	5.3	
					OsROC5-crRNA-01	0	
					OsROC5-crRNA01+*OsROC5*-crRNA02	4.5+13.7	
	Nipponbare	Callus	Base editor-CBE	*Agrobacterium*	*ALS*-T1	40	[248]
					ALS-T2	20.7	
					ALS-T3	20.7	
					DEP1	68.9	
					Waxy	13.8	
					NRT1.1B-T1	60	
					NRT1.1B-T2	44.8	
					NRT1.1B-T3	27.6	
					CDC48-T1	39.3	
					CDC48-T2	13.3	

					ALS-T1	66.7	
					ALS-T2	63.3	
					ALS-T3	47.8	
					DEP1	60	
					Waxy	13.3	
					NRT1.1B-T1	58.6	
					NRT1.1B-T2	56.6	
					NRT1.1B-T3	31	
					CDC48-T1	53.3	
					CDC48-T2	33.3	
					ALS-T1	67.8	
					ALS-T2	63.3	
					ALS-T3	69.2	
					DEP1	76.9	
					Waxy	30	
					NRT1.1B-T1	73.3	
					NRT1.1B-T2	76.9	
					NRT1.1B-T3	36	
					CDC48-T1	76.9	
					CDC48-T2	61.5	
			Base editor-ABE		*ALS*-T4	34.1	
					CDC48-T3	18.2	
					AAT	12.2	
					DEP1-T2	68.2	
					ACC	54.2	
					NRT1.1B-T4	0	
					SPL14	40.6	
					WRKY45	66.7	
					DELLA	26.5	
					ALS-T4	93.2	
					CDC48-T3	82.9	
					AAT	79.5	
					DEP1-T2	96	
					ACC	88	
					NRT1.1B-T4	13	
					SPL14	60	
					WRKY45	92.6	
					DELLA	65.2	
	TianFengB	nd	CRISPR/Cas9	*Agrobacterium*	*pWx*[a]<br>*pWxa*-5’UISS	nd	[249]

					Wx[a]		
Indica	MTU1010[250] (modified)	Callus	CRISPR/Cas9	*Agrobacterium*	*DST*	30-33.3	[251]
	Zhonghua 11	Callus	CRISPR/Cas9	*Agrobacterium*	*FWL1-8*	81.6 (avg.)	[252]
	Nipponbare	Callus	CRISPR/Cas9	*Agrobacterium*	*OsbZIP11+ OsbZIP41*<br>*OsRLI1*	nd	[253]
Indica	Super Basmati	Callus	CRISPR/Cas9	Biolistic	*OsSWEET14*-gRNA1<br>*OsSWEET14*-gRNA2<br>*OsSWEET14*-gRNA3	4.4<br>0<br>5.3	[254]
	Nipponbare	Callus	CRISPR/Cas9	*Agrobacterium*	*OsGAD3*	nd	[255]
	Zhonghua11	Callus	CRISPR/Cas9<br><br><br>CRISPR/AFID-3	*Agrobacterium*	*OsCDC48*-T2<br>*OsSPL14*<br>*OsSWEET14*<br>*OsCDC48*-T2<br>*OsSPL14*<br>*OsSWEET14*	93.6<br>50<br>64.2<br>93.5<br>65.9<br>66.2	[82]
	Kitaake	Callus	Cas9p-HDR	Biolistic	Carotenoid cassette (5.2 kb)	6.2	[256]
	nd	nd	CRISPR/Cas9<br><br><br><br>CRISPR/xCas9<br><br><br><br>CRISPR/Cas9-NG<br><br><br><br>CRISPR/eCas9-NG<br><br><br><br>Cas9n-NG-CBE<br>eCas9n-NG-CBE<br>Cas9n-ABE	nd	*OsWaxy*-TGG<br>*OsWaxy*-TGA<br>*OsWaxy*-TGT<br>*OsWaxy*-TGC<br>*OsWaxy*-TGG<br>*OsWaxy*-TGA<br>*OsWaxy*-TGT<br>*OsWaxy*-TGC<br>*OsWaxy*-TGG<br>*OsWaxy*-TGA<br>*OsWaxy*-TGT<br>*OsWaxy*-TGC<br>*OsWaxy*-TGG<br>*OsWaxy*-TGA<br>*OsWaxy*-TGT<br>*OsWaxy*-TGC<br><br><br>GGG (PAM)<br>TGG (PAM)	76.5<br>5.8<br>0<br>0<br>27.3<br>0<br>0<br>0<br>27.3<br>22.7<br>9.1<br>45.5<br>0<br>5.5<br>0<br>8.3<br>13.3<br>22.7<br>55.6<br>11.1	[257]

			Cas9n-NG-ABE		GGG (PAM)	6.5	
	Nipponbare<br>Kitaake	Callus	Base editor-ABE	*Agrobacterium*	*OsSPL14*<br><br>*SLR1*<br><br>*OsSERK2*<br><br>*Tms9-1*<br><br>*OsNRT1.1B*<br><br>*OsACC1*<br><br>*OsDEP1*<br><br>*SPX-MSF2*<br><br>*OsSPL14+OsSPL17*<br><br>*OsSPL16+OsSPL18*<br>*OsSPL13*<br><br>*SNB*	37.5<br>70.6<br>17.5<br>35<br>66.7<br>77.8<br>4.5<br>11.1<br>72.9<br>96.3<br>69.4<br>73.8<br>80<br>71.4<br>9.8<br>15.9<br>39.3<br>41.7<br>15.1<br>2.2<br>6.1<br>9.1<br>33.9	[258]
	Nipponbare	Callus	Base editor-ABE	*Agrobacterium*	*OsNRT1.1B*<br>*OsSPL14*<br>*OsWRKY45*<br>*OsSLR1*<br>*OsACC*<br>*OsNRT1.1B*<br>*OsSPL14*<br>*OsWRKY45*<br>*OsSLR1*<br>*OsACC*<br>*OsNRT1.1B*<br>*OsSPL14*<br>*OsWRKY45*<br>*OsSLR1*<br>*OsACC*	75<br>70.8<br>75<br>12.5<br>66.7<br>77.3<br>54.5<br>63.6<br>26.1<br>78.3<br>83.3<br>66.7<br>54.2<br>38.1<br>61.9	[259]

			Base editor-CBE		*OsNRT1.1B*<br>*OsSPL14*<br>*OsWRKY45*<br>*OsSLR1*<br>*OsACC*<br>*OsWaxy*-1+*OsWaxy*-2+*OsCDC48*+*OsSNB*	66.7<br>41.7<br>30.4<br>34.8<br>52.2<br>0+3.1+0+43.8<br>0+17.4+0+52.4<br>3.2+25.8+0+67.7	
	Nipponbare	Callus	Prime editor	*Agrobacterium*	*HPT*<br>*OsPDS*<br>*OsACC1*<br>*OsWx*<br>*OsACC1*<br>*OsACC1*	100<br>31.3<br>14.6<br>0<br>18<br>6.3	[260]
	nd	Callus	Prime editor	*Agrobacterium*	*OsIPA*<br>*OsTB1*<br>*OsALS*	nd<br>nd<br>approx. 1.8<br>approx. 2.2	[261]
	Zhonghua11	Callus	Prime editor	*Agrobacterium*	*OsCDC48 (deletion)*<br>*OsALS*<br>*OsCDC48 (substitution)*	21.8<br>14.3<br>2.6	[262]
	Zhonghua11	Callus	Prime editor	Biolistic	*OsEPSPS*	2.2	[263]
	Nipponbare	Callus	CRISPR/Cas9-NG<br><br><br><br><br>CRISPR/Cas9<br><br>CRISPR/xCas9	*Agrobacterium*	*OsPDS*<br>*OsLAZY*<br>*IPA1*<br>*DL*<br>*BADH2*<br>*OsPDS*<br>*OsLAZY*<br>*OsPDS*<br>*OsLAZY*	95.8<br>62.5<br>93.8<br>89.6<br>12.5<br>82.3<br>50<br>10.4<br>64.6	[264]
	Nipponbare	Callus	CRISPR/AacCas12b<br>CRISPR/AaCas12b<br>CRISPR/AacCas12b<br>CRISPR/AaCas12b<br>CRISPR/dAaCas12b-TV	*Agrobacterium*	*OsEPFL9*<br><br>*OsEPFL9*<br><br>*OsROC5+OsEPFL9+OsGS3*<br><br><br>*OsROC5+OsEPFL9+OsGS3*	36.4<br><br>54.2<br><br>16.7 (double mutants)<br>66.7 (double mutants)	[265]

	Kitaake<br>Nangeng 46	Callus (cells)<br>Callus	Base editor-ABE+CBE<br>Base editor-CBE	Biolistic<br>*Agrobacterium*<br>*Agrobacterium*	*OsALS1*	28<br>24<br>10	[266]
	Yandao 8	nd	CRISPR/Cas9	*Agrobacterium*	*OsSUN1+OsSUN2*	nd	[267]
	Nipponbare	Callus	CRISPR/Cas9-HDR	Biolistic	*OsALS*<br>*OsCCD7*	1.58-9.87<br>1.5-8.8	[268]
	Nipponbare	Callus	SaKKHn-Base editor-CBE	*Agrobacterium*	16 target sites<br>3x multiplex<br>O + 3 bp	6.3-75 (12 sites)<br>2.5-45.9<br>10-86.4	[269]
	Nipponbare	Callus	CRISPR/Cas9	*Agrobacterium*	*GS3+ GL3.1*	100+72.2	[270]
	Nipponbare	Callus	CRISPR/xCas9<br><br><br><br><br><br><br><br>xCas9-CBE	*Agrobacterium*	GAA (PAM)<br>GAT (PAM)<br>GAG (PAM)<br>GAC (PAM)<br>NGG (PAM)<br>NGA (PAM)<br>NGT (PAM)<br>NGC (PAM)<br>GAA (PAM)<br>GAT (PAM)<br>GAG (PAM)<br>NGG (PAM)<br>NGA (PAM)<br>NGT (PAM)<br>NGC (PAM)<br>GAC (PAM)	0-29.4<br>5-75<br>5-65<br>0<br>45-95<br>26.3-70<br>68.4-94.7<br>0-30<br>0-33.3<br>0-47.4<br>10-70<br>25-68.4<br>5.3-65<br>50-66.7<br>0-51.9<br>0-10.5	[271]
	Nipponbare	Callus	CRISPR/Cas9<br><br>CRISPR/Cas12a	*Agrobacterium*	*pOsXa13*<br><br>*OsPDS*	85.7<br>91.3<br>16.4<br>28.7	[272]
	VP 1636	Callus	CRISPR/Cas9	*Agrobacterium*	*Pi21*	66	[273]
	CWA<br><br><br>Taichung T65	Callus	CRISPR/Cas9	*Agrobacterium*	*PPR2*<br>*pPPR2*<br>*RMS*<br>*RMS*	28.6<br>14.3<br>T_1<br>T_1	[274]

	Nipponbare	nd	CRISPR/Cas9	*Agrobacterium*	*pWx*[b] (1 or 2 targets)	42.6	[275]
	Chao2-10	Callus	CRISPR/Cas9-AAC	*Agrobacterium*	*LAZY1*	Approx. 80	[276]
	Nipponbare	Callus	CRISPR/Cas9	*Agrobacterium*	*OsmiR535*	nd	[277]
	3-8 line	nd	CRISPR/Cas9	nd	*SAW1*	nd	[278]
	ASD16	Immature embryo	CRISPR/Cas9	*Agrobacterium*	*eIF4G*	46	[279]
	Kitaake	Callus	Base editor-ABE	*Agrobacterium*	*OsWSL5+OsZEBRA3*	1.34+38.92<br>2.81+14	[280]
	Zhonghua 11	Callus	CRISPR/Cas9	*Agrobacterium*	*Dof1-30*	5-26.3 (homo)	[281]
	Nipponbare	Callus	CRISPR/FnCas12a	*Agrobacterium*	*DL*<br>*ALS*<br>*AAO2*<br>*NCED1*	0-95.6<br>20-93.5<br>32.8-75.8<br>18.8-25	[282]
	Nipponbare	Callus	CRISPR/Cas9	*Agrobacterium*	*OsCNGC14*<br>*OsCNGC16*	nd	[283]
	nd	Callus	CRISPR/Cas9	*Agrobacterium*	*OsBHY+ OsEHY+ OsWaxy* (5 sgRNA)	nd	[284]
	Nipponbare	Callus	CRISPR/Cas9-HDR	*Agrobacterium*	*OsALS*<br><br>*OsCly1*	0-0.26 (calli)<br>0.24-1.31 (calli)<br>0-0.52 (calli)	[285]
	nd	Callus	CRISPR/ SpG<br><br><br><br>CRISPR/SpCas9<br>CRISPR/SpRY	*Agrobacterium*	NGA (PAM)<br>NGT (PAM)<br>NGC (PAM)<br>NGG (PAM)<br>NGG (PAM)<br>NAA (PAM)<br>NAC (PAM)<br>NAG (PAM)<br>NTA (PAM)<br>NTT (PAM)<br>NTC (PAM)<br>NTG (PAM)¨<br>NCA (PAM)<br>NCT (PAM)<br>NCC (PAM)<br>NCG (PAM)<br>NGA (PAM)	100<br>66.7<br>4.2-95.8<br>0-93.7<br>35.4-100<br>1.7-95.6<br>0-40<br>0-67.8<br>0<br>2.1<br>2.1-2.4<br>0-2.1<br>0<br>0<br>0-2.1<br>0-20.8<br>2.1-16.67	[286]

			ABE-nSpCas9<br>ABE-nSpG<br>ABE-nSpRY		NGT (PAM)<br>NGC (PAM)<br>NGG (PAM)<br>NGG (PAM)<br>NGG (PAM)<br>NGG (PAM)	0-6.8<br>6.2-12.5<br>0-45.8<br>4.3 (calli)<br>4.3 (calli)<br>2.1 (calli)	
	Xidao #1	Callus	CRISPR/Cas9	*Agrobacterium*	*OsIPK1*	38.1	[287]
	Zhonghua11	Callus	CRISPR/Cas9	*Agrobacterium*	*OsGSK1+OsGSK2+OsGSK3+OsGSK4*<br>*OsBZR1+OsBZR2+OsBZR3+OsBZR4*<br>*OsPPKL1+ OsPPKL2+ OsPPKL3*	14.3<br>0<br>0 (lethal?)	[288]
	Kitaake	Callus	CRISPR/SpyMacCas9<br>CRISPR/iSpyMacCas9<br><br><br>iSpymac-zCas9-CBE<br><br>iSpymac-zCas9-ABE	*Agrobacterium*	*OsPDS*-GAAG<br>*OsPDS*-GAAG<br>*OsPDS*-GAAA<br>*OsROC5*-AAAG<br>*OsROC5*- TAAA<br>*OsROC5*- CAAA<br>*OsROC5*- TAAA	20<br>57.1<br>37.5<br>88.9<br>18.8<br>38.9<br>12.5	[289]
Japonica	Songjing	nd	CRISPR/Cas9	*Agrobacterium*	*OsHd2*-uORF1<br>*OsHd2*-uORF2<br>*OsHd2*-uORF3	nd	[290]
	Nipponbare	nd	CRISPR/Cas9	*Agrobacterium*	*eIF4G*	50	[291]
Japonica	Presidio	Callus	CRISPR/Cas9	*Agrobacterium*<br>Biolistic	*OsPDS*<br>*OsPDS*	61.2<br>59.4	[292]
Japonica	TP309	Callus	CRISPR/Cas9	*Agrobacterium*	*OsGS3*	80	[293]
	Zhonghua 11	Callus	CRISPR/Cas9	*Agrobacterium*	*OsOPR7*	100	[294]
	Nipponbare	Callus	CRISPR/Cas9	*Agrobacterium*	*OsHOL1*<br>*OsHOL2*	63<br>83.3	[295]
	Nipponbare	Callus	CRISPR/Cas9	*Agrobacterium*	*OsTIR1*<br>*OsAFB2*<br>*OsTIR1+ OsAFB2*<br>*OsAFB3*<br>*OsAFB4*<br>*OsAFB5*	Nd<br>Nd<br>Nd<br>Nd<br>Nd<br>Nd	[296]
	Chunyou84 (CY84)	Callus	CRISPR/Cas9	*Agrobacterium*	*ZEP1*	73.3	[297]
	nd	nd	CRISPR/ScCas9 (Sc^{++})	*Agrobacterium*	NAG (PAM)<br>NTG (PAM)	5.6-73.8<br>0-19	[298]

					NCG (PAM)	3.6-59	
					NGG (PAM)	9.2-51.3	
			CRISPR/ScCas9		NAG (PAM)	0-82.3	
					NTG (PAM)	0-7.1	
					NCG (PAM)	0-46.7	
					NGG (PAM)	0-97.9	
			Base editor-CBE		NAG (PAM)	0-39.1	
					NGG (PAM)	0-7.4	
					NCG (PAM)	0-4.6	
					NTG (PAM)	0	
					NAG (PAM)	0-37	
					NGG (PAM)	0	
					NCG (PAM)	0	
					NTG (PAM)	0	
			Base editor-ABE		NAG (PAM)	31.2-52.6	
					NGG (PAM)	61.4-95.7	
					NCG (PAM)	50	
					NTG (PAM)	0	
					NAG (PAM)	0-68.7	
					NGG (PAM)	0-20.8	
					NCG (PAM)	10.4	
					NTG (PAM)	0	
	Kitaake	Callus	CRISPR/SpCas9-NG and	*Agrobacterium*	*OsPAL5*	62.5 and 30	[299]
			CRISPR/SpG		*OsGSK4*	52.6 and 36.2	
					OsCERK1	46.3 and 11.1	
					OsETR2	5.2 and 2.2	
			CRISPR/SpRY		*OsRLCK185*	2.6 and 2	
			Base editor-CBE		Multiple targets and PAMs	2.13 to 73.9	
			Base editor-ABE		*OsCOI2*	34.1	
					BSR-K1	4.3	
					OsMPK13	29.8	
					OsGS1	93.7	
					BSR-D1	0	
					OsGSK4	51.3	
	Nipponbare	Callus	SpCas9-NRRH	*Agrobacterium*	PDS	33.3	[300]
					LAZY1	75	
					DL	20.8	
					BADH2	2	
			SpCas9-NRCH		PDS	0	
					LAZY1	14.6	

					DL	12.5	
					BADH2	6.25	
			CRISPR/Cas9		PDS	75	
					LAZY1	20.8	
					DL	79.2	
					BADH2	8.3	
			NRRH-Base editor-CBE		IPA1-CAGC	33.3	
					Pikh-GAGC	75	
					WX-GAAA	64.6	
					IPA1-CAGC	25	
					Pikh-GAGC	43.7	
					WX-GAAA	29.2	
			NRRH-Base editor-ABE		IPA1-GAGT	100	
					ACC1-CAGA	100	
					Pi37-TAAA	52	
			NRCH-Base editor-CBE		Pita-GACT	100	
					IPA1-AACC	0	
					ALS-CACC	2	
					Pita-GACT	35.4	
					IPA1-AACC	4.2	
					ALS-CACC	20.8	
			NRCH-Base editor-ABE		Hd6-GACC	100	
					NAL1-GACT	89.6	
					PSS2-TACC	87.5	
			NRTH-Base editor-CBE		IPA1-GATA	72.9	
					D2-CATC	75	
					GS5-AATT	0	
			NRTH-Base editor-ABE		Pi36-TATT	100	
					Pi37-CATC	100	
					SPL18-AATC	100	
			SpG-Base editor-CBE		Pita-TGA	87.5	
					D2-CGC	79.2	
					ACC1-AGC	47.9	
					SLR1-GGT	37.5	
					Pita-TGA	77	
					D2-CGC	33.3	
					ACC1-AGC	29.2	
					SLR1-GGT	33.3	
			SpG-Base editor-ABE		Hd6-GGA	100	
					IPA1-AGT	100	

					ACC1-TGC	85.4	
					NAL1-TGA	79.2	
			SpRY-Base editor-CBE		TAC1-GAA	52	
					IPA1-CAG	16.7	
					ACC1-AAC	8.3	
					SLR1-TCG	0	
					TT1-CTT	0	
					TAC1-GAA	12.5	
					IPA1-CAG	6.2	
					ACC1-AAC	14.6	
					SLR1-TCG	0	
					TT1-CTT	0	
			SpRY-Base editor-ABE		SPL13-TAG	47.9	
					ACC1-CGA	22.9	
					IPA1-GCT	6.2	
					NAL1-ACT	0	
					WX-TTC	0	
	nd	Callus	CRISPR/LbCas12a	*Agrobacterium*	*OsDEP1*-TTTC	90	[301]
			CRISPR/Lb5Cas12a			54.5	
			CRISPR/BsCas12a			37.5	
			CRISPR/Mb2Cas12a			68.4	
			CRISPR/TsCas12a			100	
			CRISPR/MlCas12a			0	
			CRISPR/BoCas12a			29.2	
			CRISPR/MbCas12a			70.6	
			CRISPR/ErCas12a			44.4	
			CRISPR/LbCas12a		*OsEPFL9*-TTTG	91.7	
			CRISPR/Lb5Cas12a			68.4	
			CRISPR/BsCas12a			88.2	
			CRISPR/Mb2Cas12a			83.3	
			CRISPR/TsCas12a			85	
			CRISPR/MlCas12a			40.9	
			CRISPR/BoCas12a			14.3	
			CRISPR/MbCas12a			58.6	
			CRISPR/ErCas12a			89.3	
			CRISPR/MbCas12a		*Os09g37860*-CTTC	80	
					Os12g24050-GTTG	20	
			CRISPR/LbCas12a		16 targets	Avg. 89.3 (16x 4.8)	

	Zhonghua11	Callus	Base editor-CBE	*Agrobacterium*	*OsALS*-P171<br>*OsALS*-G628<br>*OsALS*-P171+*OsALS*-G628	35<br>50<br>19.2	[302]
Indica	Longke638S	nd	CRISPR/Cas9	*Agrobacterium*	*Bsr-d1*<br>*Pi21*<br>*ERF922*<br>*Bsr-d1/Pi21/ERF922*	82<br>68<br>73<br>71	[303]
O. alta (CCDD)	polyploid rice 1	Callus	CRISPR/Cas9<br><br><br><br><br>Base editor-CBE<br>CRISPR/Cas9	*Agrobacterium*	*OaqSH1-CC+OaqSH1-DD*<br>*OaAn-1-CC+OaAn-1-DD*<br>*OaAn-1-DD*<br>*OaSD1-CC+OaSD1-DD*<br>*OaGS3-CC+OaGS3-DD*<br>*OaIPA1-CC+OaIPA1-DD*<br>*OaGhd7-CC+OaGhd7-DD+OaDTH7-CC+OaDTH7-DD*	nd	[304]
indica	IR58025B	Callus	CRISPR/ LbCas12a	*Agrobacterium*	*xa13* –transgene-free enrichment-1<br>*xa13* –transgene-free enrichment-2	39.6<br>56.4	[305]
Oryza alta	PPR1	Callus	CRISPR/Cas9-*de novo domestication*<br><br><br><br><br><br>Base editor-CBE	*Agrobacterium*	*OaqSH1-CC+OaqSH1-DD* (1 guide)<br>*OaAn-1-CC+OaAn-1-DD* (1 guide)<br>*OaSD1-CC+OaSD1-DD* (1 guide)<br>*OaGS3* (1 guide)<br>*OaGhd7-CC+OaGhd7-DD+OaDTH7-CC+ OaDTH7-DD* (3 guides)<br>*OaIPA1-DD* (1 guide)	Nd<br>Nd<br>Nd<br>Nd<br><br>75<br>Nd	[306]
	Nipponbare	Callus	CRISPR/SpRY<br><br>SpRY-Base editor-CBE<br>SpRY-Base editor-ABE	*Agrobacterium*	*OsPDS*<br>*OsDEP1*<br>*OsDEP1*<br>*OsALS*<br>*OsPDS*	Up to 95.7<br>Up to 100<br>Up to 42.1<br>72.2<br>Up to 79	[307]
Japonica	Nanjing46	nd	CRISPR/Cas9	*Agrobacterium*	*OsPHYC*	nd	[308]
	IPB3S	Immature embryo	CRISPR/Cas9	*Agrobacterium*	*OsSD1*	nd	[309]
Japonica	Nipponbare	Callus	iSpyMacCas9-CBE<br>Base editor-CBE	*Agrobacterium*	*OsROC5*<br>*OsCGRS55*<br>*OsCGRS56*<br>*OsCGRS57*<br>*OsCGRS58*<br>*OsCGRS55*<br>*OsCGRS56*	68.8<br>18.8<br>9.5<br>30<br>16.7<br>10<br>22.7	[310]

					OsCGRS57	34.6	
					OsCGRS58	0	
					OsCGRS55	6.3	
					OsCGRS56	13.6	
					OsCGRS57	59.3	
					OsCGRS58	13	
					OsCGRS55	32	
					OsCGRS56	0	
					OsCGRS57	65.4	
					OsCGRS58	35	
					OsCGRS55	42.4	
					OsCGRS56	25	
					OsCGRS57	0	
					OsCGRS58	58.3	
					OsCGRS55	16.7	
					OsCGRS56	40.9	
					OsCGRS57	65	
					OsCGRS58	13.6	
	Nd	Callus	CRISPR/ScCas9	*Agrobacterium*	*BADH2*	5.6	[311]
					CHALK5-2	0	
					CHALK5-3	0	
					Wx-4	0	
					Sd1	16.7	
					ALS	0	
					IPA1	0	
					GS3	0	
					TB1	0	
					MADS26	0	
			CRISPR/ScCas9+		*BADH2*	22.2	
					CHALK5-2	0	
					CHALK5-3	0	
					Wx-5	0	
					Sd1	24.1	
					ALS	0	
					IPA1	3.5	
					GS3	0	
					TB1	0	
					MADS26	0	
			CRISPR/ScCas9++		*BADH2*	53.6	
					CHALK5-2	0	

					CHALK5-3	0	
					Wx-6	78.6	
					Sd1	51.8	
					ALS	0	
					IPA1	11.1	
					GS3	0	
					TB1	0	
					MADS26	0	
			ScCas9n++-base editor-		*Wx*-1	8.7	
			CBE		*Wx*-2	60.9	
					Wx-3	60.9	
					Euil-4	21.7	
					Euil-5	60.9	
					Intergenic-1	75	
					Os03g0163600	80.6	
					Os01g0101150	13.9	
					Os01g0101200	86.1	
					Intergenic-2	8.3	
Indica	TianFengB				*Wx*-1+*Wx*-2+*Wx*-3+*Euil*-4+*Euil*-5	nd	
Japonica	Guihong1<br>Zhonghua11	Callus	CRISPR/Cas9	*Agrobacterium*	*OsSWEET11*<br>*OsSWEET14*<br>*OsSULTR3;6*	nd	[312]
	Zhonghua11	Callus	CRISPR-MAD7	*Agrobacterium*	*OsALS*<br>*OsEPSPS*<br>*OsNRAMP5*	49<br>65.6<br>56.9	[74]
	Nipponbare	Callus	Nm1-base editor-ABE	*Agrobacterium*	*PDS-1*	0	[313]
					NAL1-4	0	
					WX-3	1	
					OsPDS-1	4.2	
					NAL1-4	87.5	
					WX-3	12.5	
					ACC-1	0	
					GS5-1	0	
					Hd6-1	2	
					ACC-1	25	
					GS5-1	0	
					Hd6-1	33.3	
			CRISPR/Nm1Cas9		*ALS-1*	Approx. 60	
					PDS-1	Approx. 62	
					PDS-2	Approx. 63	

			CRISPR/Nm2Cas9		*BEL-1*<br>*NAL1-1*<br>*WX-1*<br>*BADH2-1*<br>*IPA1-1*<br>*NAL1-3*<br>*PDS-3*<br>*WX-2*<br>*BADH2-2*<br>*PDS-4*<br>*ALS-3*<br>*PDS-5*<br>*DL-1*<br>*BADH2-3*<br>*IPA1-2*	Approx. 88<br>Approx. 70<br>Approx. 62<br>89.6<br>Approx. 87<br>4.2<br>6.3<br>18.8<br>29.2<br>Approx. 90<br>Approx. 20<br>Approx. 80<br>Approx. 35<br>100<br>Approx. 92	
	Nipponbare	Callus	CRISPR/Cas9 (Multiplex)	Biolistics	*OsFAD2-1*	10?	[314]
	DLMM	Callus	CRISPR/Cas9	*Agrobacterium*	*OsTTG1*	73.3	[315]
	NB7	nd	CRISPR/Cas9	Biolistic	*Xa7*	nd	[316]
	Yukihikari	Callus	CRISPR/Cas9	*Agrobacterium*	UGT74J1	nd	[317]
Indica	Y58S	Callus	CRISPR/Cas9	*Agrobacterium*	*Wx*	64.3	[318]
	Kitaake	Callus	Base editor-ABE<br>Cas9n-NG-base editor-ABE<br>SpRYn-Base editor-ABE	*Agrobacterium*	*OsMPK6*<br>*OsMPK13* site 1<br>*Tms9-1*<br>*OsWRKY45* site 1<br>*OsGS1* site 1<br>*OsACC* site 1<br>*OsGSK3*<br>*OsGSK4* site 1<br>*OsMPK6*<br>*OsSERK2* site 1<br>*OsWRKY45* site 2<br>*OsDEP2*<br>*OsETR2*<br>*OsGSK4* site 2<br>*OsJAR1*<br>*OsGS1* site 2<br>*OsGSK4* site 3	56.2 (callus)<br>87<br>97.9<br>82.5<br>94.4<br>91.7<br>72.4<br>79.3<br>93.7<br>100<br>93.7<br>77<br>83<br>92.1<br>47.5<br>93.7<br>72.9	[319]

	Nangeng 46		ScCas9n-Base editor-ABE<br><br><br><br><br>Base editor-ABE		*OsMPK13* site 2<br>*OsGS1* site 3<br>*OsGS1* site 4<br>*OsSERK2* site 2<br>*Wx*<br>*OsCPK6*<br>*OsMPK9*<br>*OsALS1+OsGS1* site 3<br>*OsTubA2+OsACC* site 2<br>*OsALS1+OsGS1* site 3+*OsTubA2+OsACC* site 2	33.3<br>95.8<br>41.7<br>66.7<br>68.7<br>10.4<br>0<br>72.9 (plants)<br>73.8<br>56.2	
	nd	nd	CRISPR/Cas9-TKC	nd	4 genes<br>4 genes<br>5 genes	61.5<br>19<br>0 (lethal gene)	[320]
	Nipponbare	nd	CRISPR/Cas9	nd	*CAO1*	nd	[321]
	Kitaake	Callus	CRISPR/Cas9	*Agrobacterium*	*OsPLT8*<br>*OsPLT9*<br>*OsPLT7*<br>*OsAP2*<br>*OsAD5*	Nd<br>Nd<br>Nd<br>Nd<br>Nd	[322]
	Kitaake	Callus	CRISPR/Cas9	*Agrobacterium*	*pOsREM20*<br>*OsREM20*	Nd<br>Nd	[323]
	Nipponbare<br><br><br><br><br><br><br><br>Wuyunjing31	Callus	eCDAL-base editor-CBE<br><br><br><br>pDuBE1-base editor-ABE	*Agrobacterium*	*ALS*<br>*BADH2*<br>*LAZY1*<br>*PDS*<br>*BADH2*<br>*FSD2*<br>*LAZY1*<br>*PDS*<br>*ALS* (dual C-to-T and A-to-G conversion)	81.3<br>95.8<br>68.8<br>87.5<br>89.6 (dual)<br>68.7 (dual)<br>72.9 (dual)<br>58.3 (dual)<br>100	[324]
	Nipponbare	nd	CRISPR/Cas9	*Agrobacterium*	*OsVQ4*	nd	[325]
Japonica	Jingeng 818	Callus	CRISPR/Cas9 knock-up	*Agrobacterium*	911 kb inversion<br>338 kb duplication	3<br>0.1	[326]
Indica	CO51	Immature embryos	CRISPR/Cas9	*Agrobacterium*	OsSWEET14	83.3	[327]
	Nipponbare	Callus	CRISPR/Cas9	*Agrobacterium*	*Os02circ25329*	14	[328]

					Os06circ02797<br>*Os03circ00204*<br>*Os05circ02465*	8.8<br>10.9<br>13.6	
Indica	MTU1010	Callus	CRISPR/Cas9	*Agrobacterium*	*OsGW2*	62.5	[329]
	Nipponbare	nd	CRISPR/Cas9	*Agrobacterium*	*Oscmt2*<br>*Oscmt3a*<br>*Oscmt3b*<br>*Osdrm1a*<br>*Osdrm1b*<br>*Osdrm2*<br>*Osdrm3*<br>*Osmet1a*<br>*OsMET1b*<br>*Oscmt2/3a*<br>*Osdrm1a/1b*<br>*Osdrm2/3*<br>*Osdrm2/cmt2*<br>*Osdrm2/cmt3a*<br>*Osdrm2/cmt2/3a*<br>*Osdrm2/3/cmt2/3a*<br>*Osdrm2/3/cmt2/3a/3b*	nd	[330]
	Pusa Basmati1 (PB1)	Callus	CRISPR/Cas9	*Agrobacterium*	*OsGA20ox2*	8.75	[331]
Indica	Ciherang-Sub1	Immature embryo	CRISPR/Cas9	*Agrobacterium*	*SUB1A*	100	[332]
	Nipponbare	Callus	CRISPR/Cas9	nd	*OsOR*	nd	[333]
	Kitaake	Callus	CRISPR/Cas9	*Agrobacterium*	*LHW*<br>*ARF15*<br>*OSH6*	nd	[334]
	RT102A	Callus	mitoTALEN	*Agrobacterium*	*orf352*	nd	[335]
	Kitaake	Callus	Base editor-CBE<br>SpRY-base editor-CBE	*Agrobacterium*	*OsALS*	47.6-94.1<br>47.6-75	[336]
	NIL-*HL6*	Callus	CRISPR/Cas9	*Agrobacterium*	*OsSPL10*	nd	[337]
	Zhonghua 11	nd	CRISPR/Cas9	nd	*SWEET2a*	nd	[338]
	Dongjin	Callus	CRISPR/Cas9	*Agrobacterium*	*OFF*	nd	[339]
Indica	ASD16	Immature embryos	CRISPR/Cas9	*Agrobacterium*	*eIF4G*	32.3-62.5	[340]

Oryza rufipogon Griff	CLCWR	Callus	CRISPR/Cas9	*Agrobacterium*	*OrNCED3*<br>*OrNCED5*<br>*OrNCED1-OrNCED3*	50-70<br>60-70<br>20-30	[341]
	Nipponbare	Callus	CRISPR/Cas9	*Agrobacterium*	*OsXLG1*<br>*OsXLG2*<br>*OsXLG4*<br>*Osxlg1&2*<br>*Osxlg1&4*<br>*Osxlg2&4*<br>*Osxlg1,2&4*	nd	[342]
	Taipei-309	Callus	CRISPR/Cas9	*Agrobacterium*	*OsLip1*	nd	[343]
	Kitaake	Callus	CRISPR/Cas9	*Agrobacterium*	*CYP76M7+CYP76M8*	nd	[344]
	Dongjin	Callus	CRISPR/Cas9	*Agrobacterium*	*OsMORE1a*	40	[345]
	Nipponbare	Callus	CRISPR/Cas9	*Agrobacterium*	*OsGA3ox1*	nd	[346]
	Kitaake	nd	CRISPR/Cas9	*Agrobacterium*	*RLK* family (1,072 members)	89	[347]
	Zhonghua11	Callus	CRISPR/Cas9-HDR	*Agrobacterium*	Marker gene excision	73.3	[348]
	Nipponbare	nd	CRISPR/Cas9	*Agrobacterium*	*TAS3a*<br>*TAS3b*<br>*TAS3c*	nd	[349]
	Nipponbare	Callus	CRISPR/Cas9	*Agrobacterium*	*OsAP47*	nd	[350]
	Zhonghua11	Callus	Prime editor	*Agrobacterium*	*OsALS*	11.3<br>2.1	[351]
	Zhonghua11	Callus	CRISPR/Cas9	*Agrobacterium*	*CDC20.1+CDC20.2+CDC20.3*	nd	[352]
Sorghum	nd	Immature embryo	CRISPR/Cas9	*Agrobacterium*	*mDsRED2*	33	[353]
	Tx430	Immature embryo	CRISPR/Cas9	*Agrobacterium*	*Sb-CENH3*	37-40	[354]
	Tx430	Immature embryo	CRISPR/Cas9	*Agrobacterium*	*k1C gene family*	92.4	[355]
	P898012	Immature embryo	CRISPR/Cas9	*Agrobacterium*	*SbFT*<br>*SbGA2ox5*	33.3<br>83.3	[356]
	Tx430	Immature embryo	CRISPR/Cas9	Biolistic	*SbLG1*	33.3	[357]

	Wheatlan	Immature embryo	CRISPR/Cas9	*Agrobacterium*	*SbBADH2*	38.9	[358]
	nd	Embryogenic callus	CRISPR/Cas9	Biolistic	*GHD7, β-kafrin, γ-kafrin, IDS1, SID1*	55.6-90.1	[35]
	RTx430, BTx623, BTx642, SC187	Immature embryos	CRISPR/Cas9	*Agrobacterium*	*pds*	16.7	[359]
Soybean	Bert	Cotyledons<br><br><br><br>Half-seed	ZFN	*A. rhizogenes*	*DCL1a, DCL1b*<br>*DCL2a, DCL2b*<br>*DCL4a+ DCL4b*<br>*RDR6a, RDR6b, HEN1a*<br>*DCL4a+ DCL4b*	0-100%<br><br><br><br>66.7%	[360]
	Bert	Half-seed	TALENs	*A. rhizogenes*	*FAD2-1A + FAD2-1B*	21%	[361]
	Jack	cotyledons<br><br><br>Somatic embryos	CRISPR/Cas9	*A. rhizogenes*<br><br><br>Biolistic	*Glyma07g14530*<br>*Glyma01g38150+ Glyma11g07220*<br>*miR1509, and miR1514*<br>*Glyma07g14530, 01g, 11gDDM1*	95 % of transgenic roots<br><br>nd	[362]
	Bert	cotyledons	CRISPR/Cas9	*A. rhizogenes*	*GS1, CHI20*	100 % of transgenic roots	[363]
	Williams	Cotyledonary node	CRISPR/Cas9	*A. rhizogenes*	*Glyma06g14180, Glyma08g02290, Glyma12g37050*	3-20 % of transgenic roots	[364]
	93B86	Embryogenic callus	CRISPR/Cas9-HDR	Biolistic	DD20<br>Genomic location<br><br><br>DD43<br>Genomic locations	Callus: OK; plants: not<br>HDR: nd<br>Callus ok Plants ok<br>HDR: nd	[365]
	Bert<br>Mutated in FAD2-1A, FAD2-1B	Half-seed	TALENS	Biolistic	*FAD3*	22%	[366]
	Hill	Cotyledonary node	CRISPR/Cas9	*A. rhizogenes*	*Rj4*<br>*Glyma.01G165800, Glyma.01G165800-D*	nd	[367]
	Bert	Half-seed	ZFN	*A. rhizogenes*	*DCL1a + DCL1b*	17%	[368]

	Jack	Cotyledon<br><br>Cotyledonary node	TALENs<br>CRISPR/Cas9<br>CRISPR/Cas9	*A. rhizogenes*<br><br><br><br>*Agrobacterium*	*PDS11, PDS18*<br><br>*PDS11+PDS18*<br><br>*PDS11+PDS18*	18-21 % of hairy roots<br>12-48% of hairy roots<br>nd	[369]
	Williams 82	Cotyledonary node	CRISPR/Cas9	*A. rhizogenes*	*Rj4*	nd	[370]
	Bert	Half-seed	TALENs<br>CRISPR/Cas9	*A. rhizogenes*	*Dcl2a, Dcl2b, Dcl3a*<br>*Drb2a+Drb2*	0-75%<br>75%	[371]
	Kariyutaka	Cotyledonary node	CRISPR/Cas9	*Agrobacterium*	*PPD1+PPD2*	nd	[372]
	Jack	Half-seed	CRISPR/Cas9	*Agrobacterium*	*FT2, GmFT5a*	36%-72%	[373]
	Jinong 38	Half-seed	CRISPR/Cas9	*Agrobacterium*	*FAD2–2*	21 %	[374]
	Tianlong 1	Cotyledonary node	CRISPR/Cas9	*Agrobacterium.*	*Hsp90A2*	nd	[375]
	Williams 82	Cotyledonary node	CRISPR/Cas9	*Agrobatcerium*	*SPL9a+SPL9b+SPL9c+SPL9d*	nd	[376]
	Harosoy	Cotyledonary node	CRISPR/Cas9	*Agrobatcerium*	*LHY1a+ LHY1b+ LHY2a+HY2b*	5%	[377]
	Maverick	Cotyledonary node	CRISPR/Cas9	*Agrobatcerium*	*GmFAD2–1A + GmFAD2–1B.*	100 %	[378]
	Jack	Cotyledonary node	CRISPR/Cas9	*Agrobacterium*	*E1*	75%	[379]
	Harosoy 63	Cotyledonary node	CRISPR/Cas9	*A. rhizogenes*	Glyma.20g148400, Glyma.20g146200, Glyma.10g246300, Glyma.20g148200, Glyma.10g037100, Glyma.03g163500, Glyma.19g164900, Glyma.13g123500,Glyma.19g164800	4-44% of hairy root	[380]
	X5<br><br>Westag 97<br><br>Maverick	Embryogenic callus<br><br><br><br>Immature embryos	ZFN	Biolistic	Targeted insertions of large fragments into the FAD2-1a gene via HDR<br>Targeted insertions of large fragments into the FAD2-1a gene via NHEJ<br>Targeted insertions of large fragments into the FAD2-1a gene via NHEJ	Callus: Via HDR: 0-9%.<br>Callus: Via NHEJ: 8-27%<br>Targeted insertion in regenerated plants via NHEJ: 0.04%	[381]
	Jack	Somatic embryos	CRISPR/Cas9	Biolistic	*CPR5*	100%	[382]
	Williams 82	Cotyledonary node	CRISPR/Cas9	*Agrobacterium*	*SWEET15a+SWEET15b*	Nd	[383]

	Williams 82	Cotyledonary node	CRISPR/Cas9	*A. rhizogenes*	*LCLa1+ LCLa2+ LCLb1+ LCLb2*	Nd	[384]
	Jack	nd	Base editing-CBE	*Agrobacterium*	*FT2a*<br>*FT4*	18%<br>6%	[385]
	Zigongdongdou<br>Jack	Half-seed	CRISPR/Cas9	*Agrobacterium*	*PRR37*	nd<br>nd	[386]
	Huachun 6	Cotyledonary node	CRISPR/Cas9	*Agrobacterium*	*Lox1+ Lox2+ Lox*	37%	[387]
	Jinong 38	nd	CRISPR/Cas9	*Agrobacterium*	*FAD2-1A +FAD2-2A*	66.67%.	[388]
	Jack	Cotyledons<br>Cotyledonary nodes	CRISPR/Cas9	*A. rhizogenes*<br>*Agrobacterium*	*F3H1+F3H2+FNSII-1*<br>*F3H1 + F3H2 + FNSII-1*	6-13%<br>44 %	[389]
	Tianlong no 1	Cotyledonary nodes	CRISPR/Cas9	*Agrobacterium*	*Nac8*	nd	[390]
	Hx3	Cotyledonary nodes	CRISPR/Cas9	*Agrobacterium*	*APa1a+ APa1b + APa1c+ APa1d*	20%	[391]
	Tianlong 1	Cotyledonary nodes	CRISPR/Cas9	*Agrobacterium*	*PRR3b*	nd	[392]
	Jack	Cotyledonary nodes	CRISPR/Cas9	*Agrobacterium*	*NMHC5*	25%	[393]
	DongNong50	Embryonic tips of mature seeds.	CRISPR/Cas9	Biolistic	*LMM2*	nd	[394]
	Tianlong1	Cotyledonary nodes	CRISPR/Cas9	*Agrobacterium*	*DCL2a + DCL2b*	nd	[395]
	Bert	Cotyledonary nodes	CRISPR/Cas9	*A. rhizogenes*	*KASII*	100%	[396]
	Maverick<br>DT26<br>Maverick<br>DT26	Cotyledons<br><br>Cotyledonary nodes	CRISPR/Cas9	*A. rhizogenes*<br><br>*Agrobacterium*	*GOLS1A + GOLS1B*<br>*GOLS1A + GOLS1B*<br>*GOLS1A + GOLS1B*<br>*GOLS1A + GmGOLS1B*	100% of roots<br>100% of roots<br>100%<br>100%	[397]
	Enrei<br>Kariyutaka	Cotyledonary nodes	CRISPR/Cas9	*Agrobacterium*	*m Bd 28 K + m Bd 30 K*	nd	[398]
	Jack	Embryogenic callus	CRISPR/Cas9	Biolistic	*m Bd 30K*	100%	[399]

	Williams 82	nd	CRISPR/Cas9	*Agrobacterium*	*MS1*	nd	[400]
	Williams 82	hypocotyl	CRISPR/Cas9	*A. rhizogenes*	*lncRNA77580*	nd	[401]
	N8855	Half-seeds	CRISPR/Cas9	*Agrobacterium*	*PPR576*	33%	[402]
	Tianlong 1	Half-seeds	CRISPR/Cas9	*A. rhizogenes*<br>*Agrobacterium*	*PLA-IIε, PLA-IIζ*	nd	[403]
	Williams 82	Cotyledonary nodes	CRISPR/Cas9	*Agrobacterium*	*LNK2a + LNK2b + LNK2c + LNK2d*	nd	[404]
	Williams 82	Cotyledonary nodes	CRISPR/Cas9	*Agrobacterium*	*FATB1a+FATB1b*	nd	[405]
	nd	nd	CRISPR/Cas9	nd	*Glyma.13G114200*	nd	[406]
	Huachun 6	nd	CRISPR/Cas9	nd	*GmJAG1+ GmJAG2*	nd	[407]
	Williams 82	Cotyledonary nodes	CRISPR/Cas9	*Agrobacterium*	*GmAMS1, GmAMS2*	14-25%	[408]
	ZP661	Cotyledonary nodes	CRISPR/Cas9	*Agrobacterium*	Glyma.13G114200	nd	[409]
	Jack	nd	CRISPR/Cas9	nd	*NMHC5*	nd	[410]
	nd	nd	CRISPR/Cas9	*A. rhizogenes*	*DRR1*	nd	[411]
	93Y21	embryonic axes	CRISPR/Cas9-HDR	*Ochrobactrum haywardense* Oh H1-8	DD38 genomic sites<br>DD51 genomic sites	HDR:3.4%<br>HDR:2 %	[412]
	Tianlong1	Cotyledonary nodes	CRISPR/Cas9	*Agrobacterium*	*E1*	nd	[413]
	Williams 82	nd	CRISPR/Cas9	*Agrobacterium*	*HSP17.9*	nd	[414]
	DS-9712	Leaf disks<br><br>Cotyledonary nodes	CRISPR/Cas9	*Agrobacterium*-mediated Disc Assay for Transient Expression<br>*Agrobacterium*	*IPK2*	60%<br><br>nd	[415]
	Tian Long 1	Cotyledonary nodes	CRISPR/Cas9	*Agrobacterium*	*BIC1a+ BIC1b + BIC2a + BIC2b*	nd	[416]

	Huachun6	Cotyledonary nodes	CRISPR/Cas9	*Agrobacterium*	*PDH1*	43%	[417]
	Tianlong No. 1	Cotyledonary nodes	CRISPR/Cas9	*Agrobacterium*	*UGT*	nd	[418]
Maize	nd	Immature embryo	CRISPR/Cas9 (HDR)	Biolistic<br>*Agrobacterium*	*LIG1, Ms26, Ms45, ALS1, ALS2*	1.3-3.9	[419]
	B73	Immature embryo	CRISPR/Cas9	*Agrobacterium*	*PSY1*	10.67	[420]
	Hi-II	Immature embryo	CRISPR/Cas9	*Agrobacterium*	*Zmzb7*	19-31	[421]
	nd	Immature embryo	CRISPR/Cas9<br>CRISPR/Cas9-HDR	Biolistic	*ARGOS8*	60-98<br>1	[422]
	Hi-II	Immature embryo	CRISPR/Cas9	*Agrobacterium*	*ZmPDS, ZmIPK1A, ZmIPK, ZmMRP4*	13.1-19.1	[423]
	ZC01	Immature embryo	CRISPR/Cas9	*Agrobacterium*	*ZmLG1, UB2, and UB3*	4.1	[424]
	B73-329	Immature embryo	CRISPR/Cas9	*Agrobacterium*	*ZmTMS5*	nd	[43]
	ZC01	Immature embryo	CRISPR/Cas9	*Agrobacterium*	*ZmMS26*	27.8	[425]
	NP2222	Immature embryo	CRISPR/Cas9	*Agrobacterium*	*MATL*	nd	[426]
	Z31	Immature embryo	CRISPR/Cas9	*Agrobacterium*	*ZmLTPg11, ZmLTPx2*	21.5-26.7	[427]
	Hi-II,<br>B104	Immature embryo	CRISPR/Cas9	*Agrobacterium*	*ZmAgo18a, ZmAgo18b, a1, a4*	16-35	[428]
	PH1V69	Immature embryo	CRISPR/Cas9-HDR	*Agrobacterium*	*NptII*	4.7	[429]
	B104	Immature embryo	CRISPR/Cas9<br>CRISPR/Cas12a	*Agrobacterium*	*gl2*	90.3-100<br>0-60	[430]
	ND73	Immature embryo	CRISPR/Cas9	*Agrobacterium*	*CDPK10*	69-93	[431]
	Hi-II	Immature embryo	CRISPR/Cas9	*Agrobacterium*	*MS8*	nd	[432]
	Hi-II	Immature embryo	CRISPR/Cas9–gRNA RNP	Biolistic	*LIG,ALS2,MS26, MS45*	2.4-9.7	[433]
	A188	Immature embryo	CRISPR/Cas9	*Agrobacterium*	*20 maize kernel development genes*	53	[434]
	Hi-II	Immature embryo	CRISPR/Cas9	*Agrobacterium*	*zb7*	66	[435]
	Hi-II	Immature embryo	CRISPR/Cas9<br>CRISPR/Cas12a	*Agrobacterium*	*Opaque2*	nd	[436]

	355, LN005M, XCW175	Immature embryo	CRISPR/Cas9	*Agrobacterium*	*ZmBADH2a, ZmBADH2b*	nd	[437]
	Cal	Immature embryo	CRISPR/Cas9	*Agrobacterium*	*GA20ox3*	nd	[438]
	PH1V5T	Immature embryo	CRISPR/Cas9-RNP	*Agrobacterium*	*pericentric inversion in chromosome*	nd	[439]
	KN5585	Immature embryo	CRISPR/Cas9-RNP	*Agrobacterium*	*Zm00001d040611, Zm00001d033267*	nd	[440]
	B104	Immature embryo	CRISPR/Cas9 CRISPR/Cas12a	*Agrobacterium*	*A842B-2-2, A842B-5-1*	51.4-100	[441]
	Hi-II	Immature embryo	CRISPR/Cas9	*Agrobacterium*	*14 TF genes*	nd	[442]
	Hi-II	Immature embryo	CRISPR/Cas9	*Agrobacterium*	*ZmPRO1, ZmPRO3*	0-90	[443]
	KN5585	Immature embryo	CRISPR/Cas9	*Agrobacterium*	*1244 candidate genes*	51-91	[444]
	nd	Immature embryo	CRISPR/Cas9	*Agrobacterium*	*br2*	nd	[445]
	Hi-II	Immature embryo	CRISPR/Cas9	*Agrobacterium*	*MADS, MYBR, AP2, RPL, PPR, lncRNA, lncRNA2*	57.1-100	[446]
	NP2222	Immature embryo	CRISPR-Cas12a-RNP	Biolistic	*Bx9*	60-100	[447]
	PH184C, PHH5G, PH1CJB, PH1V69, PH25KM, PH2DNP, PH1D84, PH1V5T, PH2DYM, PH17JT, PH1W4R, PH4257	Immature embryo	CRISPR/Cas9	Biolistic	*Wx1*	4-19	[448]
	B73	Immature embryo	CRISPR/Cas9-Prime	*Agrobacterium*	*ZmALS1, ZmALS2*	15.2-71.4	[449]
	ZC01	Immature embryo	CRISPR/Cas9	*Agrobacterium*	*ZmPHYC1, ZmPHYC2*	nd	[450]
	B73	Immature embryo	CRISPR/Cas9	*Agrobacterium*	*stiff1*	nd	[451]
	PH184C, PH1V5T	Immature embryo	CRISPR/Cas9	*Agrobacterium*	*CXE-20*	8.3	[452]
	Hi-II	Immature embryo	CRISPR/Cas9	*Agrobacterium*	*ZmGAMYB , ZmGAMYB-2*	nd	[453]
	Hi-II	Immature embryo	CRISPR/Cas9	*Agrobacterium*	*dek42*	nd	[454]
	ZC01	Immature embryo	CRISPR/Cas9	*Agrobacterium*	*ZmCCT9*	nd	[455]
	Hi-II	Immature embryo	CRISPR/Cas9	*Agrobacterium*	*ZmGB1*	nd	[456]

	ZC01	Immature embryo	CRISPR/Cas9-APOBEC1	*Agrobacterium*	*ZmALS1, ZmALS2*	nd	[457]
	B104	Immature embryo	CRISPR/Cas9	*Agrobacterium*	*ZmCLCg*	10-70	[458]
	B73	Immature embryo	CRISPR/Cas9	*Agrobacterium*	*ZmPT7*	nd	[459]
	32990700	Immature embryo	CRISPR/Cas9	*Agrobacterium*	*ZmHKT2*	nd	[460]
	B73	Immature embryo	CRISPR/Cas9	*Agrobacterium*	*ct2*	nd	[461]
	ZC01	Immature embryo	CRISPR/Cas9	*Agrobacterium*	*Zmpif3, Zmpif4, Zmpif5*	nd	[462]
	KN5585	Immature embryo	CRISPR/Cas9	*Agrobacterium*	*ZmACD6*	nd	[463]
	PHFFB	Immature embryo	CRISPR/Cas9-HDR	Biolistic	*Rf4*	nd	[464]
	ZC01	Immature embryo	CRISPR/Cas9	*Agrobacterium*	*nrpc2, smk7*	nd	[465]
	KN5585	Immature embryo	CRISPR/Cas9	*Agrobacterium*	*coi1a, jaz15*	nd	[466]
	Hi-II	Immature embryo	CRISPR/Cas9	*Agrobacterium*	*ZmMs33*	nd	[467]
	B73	Immature embryo	CRISPR/Cas9	*Agrobacterium*	*Zmsmc3*	25-100	[468]
	B73-329	Immature embryo	CRISPR/Cas9	*Agrobacterium*	*ZmCEP1*	nd	[469]
	Hi-II	Immature embryo	CRISPR/Cas9	*Agrobacterium*	*gl2*	80	[470]
	B104	Immature embryo	CRISPR/Cas9	*Agrobacterium*	*ZmSIP*	nd	[471]
	B73-329	Immature embryo	CRISPR/Cas9	*Agrobacterium*	*ZmNL4*	nd	[472]
	W22	Immature embryo	CRISPR/Cas9	*Agrobacterium*	*ZmbZIP22*	nd	[473]
	KN5585	Immature embryo	CRISPR/Cas9	*Agrobacterium*	*wx*	80-100	[474]
	Hi-II	Immature embryo	CRISPR/Cas9	*Agrobacterium*	*Zmabcg, zmfar1*	nd	[475]
	B73	Immature embryo	CRISPR/Cas9	*Agrobacterium*	*ZmGRP1*	nd	[476]
	KN5585	Immature embryo	CRISPR/Cas9	*Agrobacterium*	*AGAP, KNR6*	nd	[477]
	Hi-II	Immature embryo	CRISPR/Cas9	*Agrobacterium*	*ZmMs25*	nd	[478]
	ZC01	Immature embryo	CRISPR/Cas9	*Agrobacterium*	*srl5*	nd	[479]
	Hi-II	Immature embryo	CRISPR/Cas9	*Agrobacterium*	*lox3*	62.8	[480]

	Hi-II	Immature embryo	CRISPR/Cas9	*Agrobacterium*	*dcl5*	nd	[481]
	Hi-II	Immature embryo	CRISPR/Cas9	*Agrobacterium*	*ZmPEPR1, ZmPEPR2*	nd	[482]
	Hi-II<br>B104	Immature embryo	TALEN	*Agrobacterium*	*gl2*	3.7<br>10	[483]
	LH244	Immature embryo	CRISPR/Cas12a	*Agrobacterium*	*180 kb chromosomal region*	30–100	[484]
	Zheng 58, Hi-II	Immature embryo	CRISPR/Cas9	*Agrobacterium*	*ZmTGA9-1/-2/-3, ZmDFR1, ZmDFR2, ZmACOS5-1, ZmACOS5-2*	80-84	[485]
	KN5585	Immature embryo	CRISPR/Cas9	*Agrobacterium*	*YIGE1*	nd	[486]
	ZC01	Immature embryo	CRISPR/Cas9	*Agrobacterium*	*ZmGDIα*	41	[487]
	KN5585	Immature embryo	CRISPR/Cas9	*Agrobacterium*	*ZmPAT7*	nd	[488]
	B73	Immature embryo	CRISPR/Cas9	*Agrobacterium*	*ZmMI, ZmNTN2*	nd	[489]
	B104	Immature embryo	CRISPR/Cas9	*Agrobacterium*	*ZmCOI2a, ZmCOI2b*	nd	[490]
	Hi-II	Immature embryo	CRISPR/Cas9	*Agrobacterium*	*ZmFCP1, ZmCLE7, ZmCLE1E5*	nd	[491]
	Hi-II	Immature embryo	CRISPR/Cas9	*Agrobacterium*	*mms21*	nd	[492]
	LH244	Immature embryo	CRISPR/Cas9	*Agrobacterium*	*zmpld3, mtl, zmdmp*	nd	[493]
	Q319	Immature embryo	CRISPR/Cas9	*Agrobacterium*	*ZmThx20*	nd	[494]
	B104	Immature embryo	CRISPR/Cas9	*Agrobacterium*	*SAMBA-1, samba-2, samba-3*	nd	[495]
	Hi-II	nd	CRISPR/Cas9	nd	*GRX*	nd	[496]
Potato *Solanum tuberosum* L.	double-haploid DM	stem segments	CRISPR/Cas9	*Agrobacterium*	*StIAA2*	83.3	[497]
	Désirée<br>MSX914-10	leaf[498]	CRISPR/Cas9	*Agrobacterium*	*StALS1*	5-60<br>3-55	[499]
	Ranger Russet	Protoplast	TALEN	PEG	*VInv* (4 alleles)	11-33	[500]
	Ranger Russet	explant	TALEN-knock-in	*Agrobacterium*	*mStALS*	7	[501]
	D52	leaf	pLSLm+CRISPR	*Agrobacterium*	*ALS1*	32.2	[502]

			pLSLm+TALEN<br>pLSLm+TALEN/RT2			33.3<br>34.5	
	Désirée	protoplast	TALEN	PEG	*ALS*	10	[503]
	Sassy	nd	TALEN	*Agrobacterium*	*StSSR2*	6.9	[504]
	Kuras (tetraploid)	protoplast	CRISPR/Cas9	PEG	*GBSS*	10.3	[505]
	Désirée	internode	CRISPR/Cas9	*Agrobacterium*	*StMYB44*	81.8	[506]
	Kuras (tetraploid)	protoplast	CRISPR/Cas9-RNP	PEG	*GBSS*	9	[507]
	Sayaka	Stem internodes	CRISPR/Cas9	*Agrobacterium*	*GBSSI (2x gRNA)*<br>*GBSSI (3x gRNA)*	54 (>1 allele)<br>28 (all 4 alleles)	[508]
	Mayqueen	Shoot	CRISPR/Cas9	*A. rhizogenes*	*St16DOX*	nd	[509]
	Phureja S15-65	internodes	CRISPR/Cas9	*Agrobacterium*	*S-RNase*	5.2	[510]
	Désirée	Stem and petiole	CRISPR/CBE	*Agrobacterium*	*StALS1*	25	[511]
	Sassy	Internodes	TALEN	*Agrobacterium*	*SSR2*	nd	[512]
	Xuan Shu 2	Stem segments	CRISPR/Cas9	*Agrobacterium*	*StPDS*	46.7	[513]
	DRH 195<br>DRH 310	Leaf	CRISPR/Cas9	*Agrobacterium*	*S-RNase*	4.3<br>3.8	[514]
	Désirée	protoplast	CRISPR/Cas9	PEG	*GBSS*	35	[515]
	Désirée	Stem<br>protoplast	CRISPR/Cas9	*Agrobacterium*<br>PEG	*SBE1+SBE2*	6 (large del.)<br>1.5	[516]
	Désirée	protoplast	Base editing-ABE	PEG	*StGBSS*	6.5	[169]
	Désirée<br><br><br>Furia	Stem, petiole<br><br>protoplast	CRISPR/Cas9<br>Base editing-CBE<br>CRISPR/Cas9 (transient)	*Agrobacterium*<br><br>PEG	*StGBSSI*<br>*StGBSSI (2x)*<br>*StGBSSI (2x)*<br>*StGBSSI*	71<br>86.6-89.6<br>0.7-8.6<br>16	[517]
Solanum chacoense	DMF1	Stem	CRISPR/Cas9	*A. rhizogenes*	*StPDS (2x)*	25-100	[518]
	Sassy	Stem	TALEN	*Agrobacterium*	*SSR2*	5.1	[519]
	Désirée	Protoplast	CRISPR/Cas9-RNP	PEG	*StPPO2*	24-68	[520]
	Désirée	Stem, petiole	CRISPR/Cas9	*Agrobacterium*	*StSS6*	50	[521]

	Désirée	Stem, petiole	CRISPR/SaCas9<br>CRISPR/SaCas9-CBE	*Agrobacterium*	*StGBSSI +StDMR6-1*<br>*StDMR6-1*	0+33<br>8	[522]
	Désirée	Stem, petiole	CRISPR/SpCas9-NG<br>CRISPR/SpCas9-NG-CBE<br>CRISPR/SpCas9-NG-ABE	*Agrobacterium*	*StDMR6-1+ StGBSSI*	0+10<br>9+64<br>8+48	[523]
	Désirée	Tuber<br>Leaf	CRISPR/Cas9	*Agrobacterium*	*PDS*	47-88<br>64-78	[524]
	Chicago	meristem	CRISPR/Cas9-RNP	Biolistics<br>Vacuum infiltration	Coilin	nd	[525]
	Désirée	Protoplast<br><br>internodes	CRISPR/Cas9-RNP<br>CRISPR/Cas9<br>CRISPR/Cas9	PEG<br><br>*Agrobacterium*	*StPPO2*	18.4<br>31.9<br>9.6	[526]
	Désirée<br><br><br><br><br>King Edward	Leaf	CRISPR/Cas9	*Agrobacterium*	*StMLO1*<br>*StHDS*<br>*StTTM2*<br>*StCHL1*<br>*StDMR6-2*<br><br>*StDND1*<br>*StDMR6-1*	33<br>14<br>16.1<br>76<br>23.9<br>14.2<br>3.9<br>11.7	[527]
	Atlantic	Stem segmenst	CRISPR/Cas9	*Agrobacterium*	*StSSR2*	46	[528]
	Sayaka	Stem internodes	CRISPR/Cas9	*Agrobacterium*	*SBE3*	71 (8 all 4 alleles)	[529]
	Désirée	Protoplast	CRISPR/Cas9-RNP	PEG	*Sbe1*<br>*Sbe1+Sbe2*	52<br>72	[530]
	Yagana-INIA	Internode, leaves	CRISPR/Cas9	*Agrobacterium*	*StPPO1*<br>*StPPO2*<br>*StvacINV1*<br>*StBAM1*	1<br>0<br>0<br>4.7	[531]
	CE3027	nd	CRISPR/Cas9	*Agrobacterium*	*pStFLORE*	2	[532]
	Russet Burbank	Internodal stem	CRISPR/Cas9-HDR	*Agrobacterium*	*StCCoAOMT*	6.2	[533]
	Désirée	Leaf disc	CRISPR/Cas9	*Agrobacterium*	*VInv*	nd	[534]
Cassava *(Manihot*	60444<br>TME 204	Friable embryogenic callus (FEC)	CRISPR/Cas9	*Agrobacterium*	*MePDS-1/MePDS-2*	47<br>22	[535]

esculenta Crantz*)*							
	60444	Friable embryogenic callus (FEC)	CRISPR/Cas9	*Agrobacterium*	*MeGBSS*<br>*MePTST1*	86<br>100	[536]
	60444	Friable embryogenic callus (FEC)	CRISPR/Cas9	*Agrobacterium*	*nCBP-1*<br>*nCBP-2*<br>*nCBP-1/2*	Avg. 91	[537]
	TME 419	Friable embryogenic callus (FEC)	CRISPR/Cas9-HDR-based gene tagging	*Agrobacterium*	*MeSWEET10a(GFP-tag)*	2	[538]
	TME 419<br>TME 204	Friable embryogenic callus (FEC)	CRISPR/Cas9-HDR	*Agrobacterium*	EPSPS (TIPA) (3.2 kb)	0.13 events per cm^3 settled cell volume	[539]
	60444	Friable embryogenic callus (FEC)	CRISPR/Cas9	*Agrobacterium*	SBE2	93.02	[540]
Foxtail millet (*Setaria italica* L.)	Ci846	Callus	CRISPR/Cas9	*Agrobacterium*	*SiNP1*	nd	[541]
S. viridis (green foxtail)		Callus	CRISPR/Cas9	*Agrobacterium*	*SvLes1* (2 targets)	nd	[542]
Foxtail millet (*Setaria italica*)	Yugu1<br><br><br><br>Xiaomi	Callus	CRISPR/Cas9<br><br><br><br><br>Base editing-CBE<br>Base editing-ABE<br>CRISPR/SpCas9 multiplex (MCTU)	*Agrobacterium*	*FMBP*<br>*Dof4*<br>*BADH2*<br>*GBSS1*<br>*Dof4*<br>*ALS*<br>*ACC*<br>*Dof4*, *BADH2*, *GBSS1*, and *IPK1*	100<br>100<br>83.3<br>100<br>100<br>50<br>30.8<br>80 (quadruple)	[543]

[1] Y. Han *et al.*, "Highly efficient and genotype-independent barley gene editing based on anther culture," *Plant Commun.*, vol. 2, no. 2, p. 100082, Mar. 2021, doi: 10.1016/J.XPLC.2020.100082.

[2] T. Lawrenson *et al.*, "Induction of targeted, heritable mutations in barley and Brassica oleracea using RNA-guided Cas9 nuclease," *Genome Biol. 2015 161*, vol. 16, no. 1, pp. 1–13, Nov. 2015, doi: 10.1186/S13059-015-0826-7.

[3] M. Gurushidze, G. Hensel, S. Hiekel, S. Schedel, V. Valkov, and J. Kumlehn, "True-Breeding Targeted Gene Knock-Out in Barley Using Designer TALE-Nuclease in Haploid Cells," *PLoS One*, vol. 9, no. 3, p. e92046, Mar. 2014, doi: 10.1371/JOURNAL.PONE.0092046.

[4] T. Wendt *et al.*, "TAL effector nucleases induce mutations at a pre-selected location in the genome of primary barley transformants," *Plant Mol. Biol.*, vol. 83, no. 3, p. 279, Oct. 2013, doi: 10.1007/S11103-013-0078-4.

[5] N. Budhagatapalli, T. Rutten, M. Gurushidze, J. Kumlehn, and G. Hensel, "Targeted Modification of Gene Function Exploiting Homology-Directed Repair of TALEN-Mediated Double-Strand Breaks in Barley," *G3 Genes|Genomes|Genetics*, vol. 5, no. 9, pp. 1857–1863, Sep. 2015, doi: 10.1534/G3.115.018762.

[6] M. Li *et al.*, "Leaf Variegation and Impaired Chloroplast Development Caused by a Truncated CCT Domain Gene in albostrians Barley," *Plant Cell*, vol. 31, no. 7, pp. 1430–1445, Jul. 2019, doi: 10.1105/TPC.19.00132.

[7] E. Kapusi, M. Corcuera-Gómez, S. Melnik, and E. Stoger, "Heritable Genomic Fragment Deletions and Small Indels in the Putative ENGase Gene Induced by CRISPR/Cas9 in Barley," *Front. Plant Sci.*, vol. 0, p. 540, May 2017, doi: 10.3389/FPLS.2017.00540.

[8] T. Lawrenson, A. Hinchliffe, M. Clarke, Y. Morgan, and W. Harwood, "In-planta Gene Targeting in Barley Using Cas9 With and Without Geminiviral Replicons," *Front. Genome Ed.*, vol. 0, p. 14, Jun. 2021, doi: 10.3389/FGEED.2021.663380.

[9] P. Křenek *et al.*, "CRISPR/Cas9-Induced Loss-of-Function Mutation in the Barley Mitogen-Activated Protein Kinase 6 Gene Causes Abnormal Embryo Development Leading to Severely Reduced Grain Germination and Seedling Shootless Phenotype," *Front. Plant Sci.*, vol. 0, p. 1307, Jul. 2021, doi: 10.3389/FPLS.2021.670302.

[10] R. E. Hoffie *et al.*, "Targeted Knockout of Eukaryotic Translation Initiation Factor 4E Confers Bymovirus Resistance in Winter Barley ," *Frontiers in Genome Editing* , vol. 3. 2021, [Online]. Available: https://www.frontiersin.org/articles/10.3389/fgeed.2021.784233.

[11] R. E. Hoffie, I. Otto, H. Hisano, and J. Kumlehn, "Site-Directed Mutagenesis in Barley Using RNA-Guided Cas Endonucleases During Microspore-Derived Generation of Doubled Haploids BT - Doubled Haploid Technology: Volume 1: General Topics, Alliaceae, Cereals," J. M. Segui-Simarro, Ed. New York, NY: Springer US, 2021, pp. 199–214.

[12] S. Gasparis, M. Kała, M. Przyborowski, L. A. Łyżnik, W. Orczyk, and A. Nadolska-Orczyk, "A simple and efficient CRISPR/Cas9 platform for induction of single and multiple, heritable mutations in barley (Hordeum vulgare L.)," *Plant Methods 2018 141*, vol. 14, no. 1, pp. 1–14, Dec. 2018, doi: 10.1186/S13007-018-0382-8.

[13] G. Garcia-Gimenez *et al.*, "Targeted mutation of barley (1,3;1,4)-β-glucan synthases reveals complex

relationships between the storage and cell wall polysaccharide content," vol. 104, no. 4, pp. 1009–1022, Nov. 2020.

[14] Z. Zeng *et al.*, "Functional dissection of HGGT and HPT in barley vitamin E biosynthesis via CRISPR/Cas9-enabled genome editing," *Ann. Bot.*, vol. 126, no. 5, pp. 929–942, Oct. 2020, doi: 10.1093/AOB/MCAA115.

[15] J. H. Lee, H. J. Won, P. Hoang Nguyen Tran, S. mi Lee, H. Y. Kim, and J. H. Jung, "Improving lignocellulosic biofuel production by CRISPR/Cas9-mediated lignin modification in barley," *GCB Bioenergy*, vol. 13, no. 4, pp. 742–752, Apr. 2021.

[16] A. Kis, É. Hamar, G. Tholt, R. Bán, and Z. Havelda, "Creating highly efficient resistance against wheat dwarf virus in barley by employing CRISPR/Cas9 system," *Plant Biotechnology Journal*, vol. 17, no. 6. 2019, doi: 10.1111/pbi.13077.

[17] Y. Li *et al.*, "New D hordein alleles were created in barley using CRISPR/Cas9 genome editing," vol. 48, no. 2, pp. 131–138, Jun. 2020.

[18] S. Y. Kim, T. Bengtsson, N. Olsson, V. Hot, L. H. Zhu, and I. Åhman, "Mutations in Two Aphid-Regulated β-1,3-Glucanase Genes by CRISPR/Cas9 Do Not Increase Barley Resistance to Rhopalosiphum padi L," *Front. Plant Sci.*, vol. 11, 2020, doi: 10.3389/fpls.2020.01043.

[19] Y. Zhong, A. Blennow, O. Kofoed-Enevoldsen, D. Jiang, and K. H. Hebelstrup, "Protein Targeting to Starch 1 is essential for starchy endosperm development in barley," *J. Exp. Bot.*, vol. 70, no. 2, pp. 485–496, Jan. 2019.

[20] T. Vlčko and L. Ohnoutková, "Allelic variants of CRISPR/Cas9 induced mutation in an inositol trisphosphate 5/6 kinase gene manifest different phenotypes in Barley," *Plants*, vol. 9, no. 2, 2020, doi: 10.3390/plants9020195.

[21] G. K. Kirschner *et al.*, "ENHANCED GRAVITROPISM 2 encodes a STERILE ALPHA MOTIF-containing protein that controls root growth angle in barley and wheat," *Proc. Natl. Acad. Sci. U. S. A.*, vol. 118, no. 35, 2021, doi: 10.1073/pnas.2101526118.

[22] M. Galli *et al.*, "CRISPR/SpCas9-mediated double knockout of barley Microrchidia MORC1 and MORC6a reveals their strong involvement in plant immunity, transcriptional gene silencing and plant growth," *Plant Biotechnol. J.*, vol. 20, no. 1, pp. 89–102, Jan. , doi: 10.1111/pbi.13697.

[23] K. Holubová, G. Hensel, P. Vojta, P. Tarkowski, V. Bergougnoux, and P. Galuszka, "Modification of Barley Plant Productivity Through Regulation of Cytokinin Content by Reverse-Genetics Approaches," *Front. Plant Sci.*, vol. 0, p. 1676, 2018, doi: 10.3389/FPLS.2018.01676.

[24] N. Kumar, M. Galli, J. Ordon, J. Stuttmann, K.-H. H. Kogel, and J. Imani, "Further analysis of barley MORC1 using a highly efficient RNA-guided Cas9 gene-editing system," *Plant Biotechnol. J.*, vol. 16, no. 11, 2018, doi: 10.1111/pbi.12924.

[25] C. Selva *et al.*, "HvLEAFY controls the early stages of floral organ specification and inhibits the formation of multiple ovaries in barley," *Plant J.*, 2021, doi: 10.1111/tpj.15457.

[26] J. R. Shoesmith *et al.*, "APETALA2 functions as a temporal factor together with BLADE-ON-PETIOLE2 and MADS29 to control flower and grain development in barley," *Dev.*, vol. 148, no. 5, 2021, doi:

10.1242/dev.194894.

[27] O. Kershanskaya, D. Nelidova, G. Esenbaeva, G. Mukiyanova, A. Karabekova, and S. Nelidov, “New breakthrough CRISPR/Cas9 biotechnology of genome editing for creation of elite barley cultivars in Kazakhstan,” *J. Biotechnol.*, vol. 305, 2019, doi: 10.1016/j.jbiotec.2019.05.079.

[28] Q. Yang *et al.*, “Mutation of the D-hordein gene by RNA-guided Cas9 targeted editing reducing the grain size and changing grain compositions in barley,” *Food Chem.*, vol. 311, 2020, doi: 10.1016/j.foodchem.2019.125892.

[29] H. Hisano *et al.*, “Regulation of germination by targeted mutagenesis of grain dormancy genes in barley,” *Plant Biotechnol. J.*, 2021, doi: 10.1111/pbi.13692.

[30] M. Panting, I. B. Holme, J. M. Björnsson, Y. Zhong, and H. Brinch-Pedersen, “CRISPR/Cas9 and Transgene Verification of Gene Involvement in Unfolded Protein Response and Recombinant Protein Production in Barley Grain,” *Front. Plant Sci.*, vol. 0, p. 2501, Nov. 2021, doi: 10.3389/FPLS.2021.755788.

[31] S. D. Karunarathne, Y. Han, X. Q. Zhang, and C. Li, “CRISPR/Cas9 gene editing and natural variation analysis demonstrate the potential for HvARE1 in improvement of nitrogen use efficiency in barley,” *J. Integr. Plant Biol.*, vol. 64, no. 3, pp. 756–770, Mar. 2022, doi: 10.1111/JIPB.13214.

[32] T. de Souza Moraes *et al.*, “The TCP transcription factor HvTB2 heterodimerizes with VRS5 and controls spike architecture in barley,” *Plant Reprod.*, vol. 35, no. 3, pp. 205–220, Sep. 2022, doi: 10.1007/S00497-022-00441-8/FIGURES/5.

[33] L. Fu *et al.*, “Vacuolar H+-pyrophosphatase HVP10 enhances salt tolerance via promoting Na+ translocation into root vacuoles,” *Plant Physiol.*, vol. 188, no. 2, pp. 1248–1263, Feb. 2022, doi: 10.1093/PLPHYS/KIAB538.

[34] Q. Yang *et al.*, “Editing of the starch synthase IIa gene led to transcriptomic and metabolomic changes and high amylose starch in barley,” *Carbohydr. Polym.*, vol. 285, p. 119238, Jun. 2022, doi: 10.1016/J.CARBPOL.2022.119238.

[35] K. Massel, Y. Lam, J. Hintzsche, N. Lester, J. R. Botella, and I. D. Godwin, “Endogenous U6 promoters improve CRISPR/Cas9 editing efficiencies in Sorghum bicolor and show potential for applications in other cereals,” *Plant Cell Rep.*, vol. 41, no. 2, 2022, doi: 10.1007/s00299-021-02816-z.

[36] I. B. Holme *et al.*, “Evaluation of the mature grain phytase candidate HvPAPhy_a gene in barley (Hordeum vulgare L.) using CRISPR/Cas9 and TALENs,” *Plant Mol. Biol.*, vol. 95, no. 1, pp. 111–121, 2017, doi: 10.1007/s11103-017-0640-6.

[37] Y. Wang *et al.*, “Simultaneous editing of three homoeoalleles in hexaploid bread wheat confers heritable resistance to powdery mildew,” *Nat. Biotechnol. 2014 329*, vol. 32, no. 9, pp. 947–951, Jul. 2014, doi: 10.1038/nbt.2969.

[38] Y. Zhang *et al.*, “Simultaneous modification of three homoeologs of TaEDR1 by genome editing enhances powdery mildew resistance in wheat,” *Plant J.*, vol. 91, no. 4, pp. 714–724, Aug. 2017, doi: 10.1111/TPJ.13599.

[39] Z. Liang *et al.*, “Efficient DNA-free genome editing of bread wheat using CRISPR/Cas9 ribonucleoprotein complexes,” *Nat. Commun. 2017 81*, vol. 8, no. 1, pp. 1–5, Jan. 2017, doi: 10.1038/ncomms14261.

[40] S. Sánchez-León *et al.*, “Low-gluten, nontransgenic wheat engineered with CRISPR/Cas9,” *Plant Biotechnol. J.*, vol. 16, no. 4, pp. 902–910, Apr. 2018.

[41] Y. Zhang *et al.*, “Efficient and transgene-free genome editing in wheat through transient expression of CRISPR/Cas9 DNA or RNA,” *Nat. Commun. 2016 71*, vol. 7, no. 1, pp. 1–8, Aug. 2016, doi: 10.1038/ncomms12617.

[42] F. Abe *et al.*, “Genome-Edited Triple-Recessive Mutation Alters Seed Dormancy in Wheat,” *Cell Rep.*, vol. 28, no. 5, pp. 1362-1369.e4, Jul. 2019.

[43] J. Hu *et al.*, “A barley stripe mosaic virus-based guide RNA delivery system for targeted mutagenesis in wheat and maize,” *Mol. Plant Pathol.*, vol. 20, no. 10, 2019, doi: 10.1111/mpp.12849.

[44] R. M. Howells, M. Craze, S. Bowden, and E. J. Wallington, “Efficient generation of stable, heritable gene edits in wheat using CRISPR/Cas9,” *BMC Plant Biol.*, vol. 18, no. 1, 2018, doi: 10.1186/s12870-018-1433-z.

[45] T. Čermák *et al.*, “A multipurpose toolkit to enable advanced genome engineering in plants,” *Plant Cell*, vol. 29, no. 6, pp. 1196–1217, Jun. 2017, doi: 10.1105/tpc.16.00922.

[46] Z. Zhang *et al.*, “Development of an Agrobacterium-delivered CRISPR/Cas9 system for wheat genome editing,” *Plant Biotechnol. J.*, vol. 17, no. 8, 2019, doi: 10.1111/pbi.13088.

[47] M. J. Milner, M. Craze, M. S. Hope, and E. J. Wallington, “Turning Up the Temperature on CRISPR: Increased Temperature Can Improve the Editing Efficiency of Wheat Using CRISPR/Cas9,” *Front. Plant Sci.*, vol. 11, 2020, doi: 10.3389/fpls.2020.583374.

[48] Z. Liang *et al.*, “Genome editing of bread wheat using biolistic delivery of CRISPR/Cas9 in vitro transcripts or ribonucleoproteins,” *Nat. Protoc.*, vol. 13, no. 3, 2018, doi: 10.1038/nprot.2017.145.

[49] S. Zhang *et al.*, “Targeted mutagenesis using the Agrobacterium tumefaciens-mediated CRISPR-Cas9 system in common wheat,” *BMC Plant Biol.*, vol. 18, no. 1, 2018, doi: 10.1186/s12870-018-1496-x.

[50] J. Li *et al.*, “Modification of starch composition, structure and properties through editing of TaSBEIIa in both winter and spring wheat varieties by CRISPR/Cas9,” *Plant Biotechnol. J.*, vol. 19, no. 5, 2021, doi: 10.1111/pbi.13519.

[51] Y. Kamiya, F. Abe, M. Mikami, M. Endo, and K. Kawaura, “A rapid method for detection of mutations induced by crispr/cas9-based genome editing in common wheat,” *Plant Biotechnol.*, vol. 37, no. 2, 2020, doi: 10.5511/PLANTBIOTECHNOLOGY.20.0404B.

[52] J. Li, Z. Wang, G. He, L. Ma, and X. W. Deng, “CRISPR/Cas9-mediated disruption of TaNP1 genes results in complete male sterility in bread wheat,” *J. Genet. Genomics*, vol. 47, no. 5, 2020, doi: 10.1016/j.jgg.2020.05.004.

[53] J. Gil-Humanes *et al.*, “High-efficiency gene targeting in hexaploid wheat using DNA replicons and CRISPR/Cas9,” *Plant J.*, vol. 89, no. 6, pp. 1251–1262, 2017, doi: 10.1111/tpj.13446.

[54] A. Okada *et al.*, “CRISPR/Cas9-mediated knockout of Ms1 enables the rapid generation of male-sterile hexaploid wheat lines for use in hybrid seed production,” *Plant Biotechnol. J.*, vol. 17, no. 10, pp. 1905–1913, Oct. 2019, doi: https://doi.org/10.1111/pbi.13106.

[55] J. Li *et al.*, “Efficient multiplex genome editing by CRISPR/Cas9 in common wheat,” *Plant Biotechnol. J.*, vol. 19, no. 3.

[56] H. Liu *et al.*, “Efficient induction of haploid plants in wheat by editing of TaMTL using an optimized Agrobacterium-mediated CRISPR system,” *J. Exp. Bot.*, vol. 71, no. 4, pp. 1337–1349, Feb. 2020, doi: 10.1093/JXB/ERZ529.

[57] H. Hamada, Y. Liu, Y. Nagira, R. Miki, N. Taoka, and R. Imai, “Biolistic-delivery-based transient CRISPR/Cas9 expression enables in planta genome editing in wheat,” *Sci. Rep.*, vol. 8, no. 1, 2018, doi: 10.1038/s41598-018-32714-6.

[58] F. Camerlengo *et al.*, “CRISPR-Cas9 Multiplex Editing of the α-Amylase/Trypsin Inhibitor Genes to Reduce Allergen Proteins in Durum Wheat,” *Front. Sustain. Food Syst.*, vol. 4, 2020, doi: 10.3389/fsufs.2020.00104.

[59] S. Ibrahim, B. Saleem, N. Rehman, S. A. Zafar, M. K. Naeem, and M. R. Khan, “CRISPR/Cas9 mediated disruption of Inositol Pentakisphosphate 2-Kinase 1 (TaIPK1) reduces phytic acid and improves iron and zinc accumulation in wheat grains,” *J. Adv. Res.*, 2021, doi: 10.1016/j.jare.2021.07.006.

[60] S. Zhang *et al.*, “CRISPR/Cas9-mediated genome editing for wheat grain quality improvement,” *Plant Biotechnol. J.*, vol. 19, no. 9, 2021, doi: 10.1111/pbi.13647.

[61] F. Hahn, L. Sanjurjo Loures, C. A. Sparks, K. Kanyuka, and V. Nekrasov, “Efficient crispr/cas-mediated targeted mutagenesis in spring and winter wheat varieties,” *Plants*, vol. 10, no. 7, 2021, doi: 10.3390/plants10071481.

[62] S. Raffan *et al.*, “Wheat with greatly reduced accumulation of free asparagine in the grain, produced by CRISPR/Cas9 editing of asparagine synthetase gene TaASN2,” *Plant Biotechnol. J.*, vol. 19, no. 8, 2021, doi: 10.1111/pbi.13573.

[63] H. Tang *et al.*, “Fertility recovery of wheat male sterility controlled by Ms2 using CRISPR/Cas9,” *Plant Biotechnol. J.*, vol. 19, no. 2, 2021, doi: 10.1111/pbi.13482.

[64] W. Wang *et al.*, “Gene editing of the wheat homologs of TONNEAU1-recruiting motif encoding gene affects grain shape and weight in wheat,” *Plant J.*, vol. 100, no. 2, 2019, doi: 10.1111/tpj.14440.

[65] W. Wang *et al.*, “Transgenerational CRISPR-Cas9 Activity Facilitates Multiplex Gene Editing in Allopolyploid Wheat,” *Cris. J.*, vol. 1, no. 1, 2018, doi: 10.1089/crispr.2017.0010.

[66] H. Zhou, L. Xu, F. Li, and Y. Li, “Transcriptional regulation by CRISPR/dCas9 in common wheat,” *Gene*, vol. 807, p. 145919, Jan. 2022.

[67] A. Jouanin *et al.*, “Outlook for coeliac disease patients: Towards bread wheat with hypoimmunogenic gluten by gene editing of α- And γ-gliadin gene families,” *BMC Plant Biol.*, vol. 19, no. 1, 2019, doi: 10.1186/s12870-019-1889-5.

[68] M. Singh, M. Kumar, M. C. Albertsen, J. K. Young, and A. M. Cigan, “Concurrent modifications in the three homeologs of Ms45 gene with CRISPR-Cas9 lead to rapid generation of male sterile bread wheat (Triticum aestivum L.),” *Plant Mol. Biol.*, vol. 97, no. 4–5, 2018, doi: 10.1007/s11103-018-0749-2.

[69] E. K. Brauer *et al.*, “Genome Editing of a Deoxynivalenol-Induced Transcription Factor Confers

Resistance to Fusarium graminearum in Wheat," *Mol. Plant-Microbe Interact.*, vol. 33, no. 3, 2020, doi: 10.1094/MPMI-11-19-0332-R.

[70] D. Kim, M. Hager, E. Brant, and H. Budak, "Efficient genome editing in wheat using Cas9 and Cpf1 (AsCpf1 and LbCpf1) nucleases," *Funct. Integr. Genomics*, vol. 21, no. 3–4, 2021, doi: 10.1007/s10142-021-00782-z.

[71] J. M. Debernardi *et al.*, "A GRF–GIF chimeric protein improves the regeneration efficiency of transgenic plants," *Nat. Biotechnol.*, pp. 1–6, Oct. 2020, doi: 10.1038/s41587-020-0703-0.

[72] M. Zheng *et al.*, "Histone acetyltransferase TaHAG1 acts as a crucial regulator to strengthen salt tolerance of hexaploid wheat," *Plant Physiol.*, vol. 186, no. 4, 2021, doi: 10.1093/plphys/kiab187.

[73] C. Dong *et al.*, "Combining a New Exome Capture Panel With an Effective varBScore Algorithm Accelerates BSA-Based Gene Cloning in Wheat," *Front. Plant Sci.*, vol. 11, 2020, doi: 10.3389/fpls.2020.01249.

[74] Q. Lin *et al.*, "Genome editing in plants with MAD7 nuclease," *J. Genet. Genomics*, vol. 48, no. 6, 2021, doi: 10.1016/j.jgg.2021.04.003.

[75] M. D. Rey *et al.*, "Magnesiumincreases homoeologous crossover frequency during meiosis in ZIP4 (Ph1 gene) mutant wheat-wild relative hybrids," *Front. Plant Sci.*, vol. 9, 2018, doi: 10.3389/fpls.2018.00509.

[76] Y. Liu *et al.*, "In planta Genome Editing in Commercial Wheat Varieties," *Front. Plant Sci.*, vol. 12, 2021, doi: 10.3389/fpls.2021.648841.

[77] R. Imai *et al.*, "In planta particle bombardment (IPB): A new method for plant transformation and genome editing," *Plant Biotechnol.*, vol. 37, no. 2, 2020, doi: 10.5511/PLANTBIOTECHNOLOGY.20.0206A.

[78] C. Liu *et al.*, "Extension of the in vivo haploid induction system from diploid maize to hexaploid wheat," *Plant Biotechnol. J.*, vol. 18, no. 2, 2020, doi: 10.1111/pbi.13218.

[79] H. Han *et al.*, "Generation of a high-efficiency adenine base editor with TadA8e for developing wheat dinitroaniline-resistant germplasm," *Crop J.*, 2021, doi: 10.1016/j.cj.2021.08.006.

[80] P. Wang *et al.*, "TaPHT1;9-4B and its transcriptional regulator TaMYB4-7D contribute to phosphate uptake and plant growth in bread wheat," *New Phytol.*, vol. 231, no. 5, 2021, doi: 10.1111/nph.17534.

[81] C. Li *et al.*, "Expanded base editing in rice and wheat using a Cas9-adenosine deaminase fusion," *Genome Biol.*, vol. 19, no. 1, p. 59, 2018, doi: 10.1186/s13059-018-1443-z.

[82] S. Wang *et al.*, "Precise, predictable multi-nucleotide deletions in rice and wheat using APOBEC–Cas9," *Nat. Biotechnol.*, vol. 38, no. 12, pp. 1460–1465, 2020, doi: 10.1038/s41587-020-0566-4.

[83] Z. Liang, K. Chen, Y. Yan, Y. Zhang, and C. Gao, "Genotyping genome-edited mutations in plants using CRISPR ribonucleoprotein complexes," *Plant Biotechnol. J.*, vol. 16, no. 12, 2018, doi: 10.1111/pbi.12938.

[84] Y. Si *et al.*, "Ne2, a typical CC–NBS–LRR-type gene, is responsible for hybrid necrosis in wheat," *New Phytol.*, vol. 232, no. 1, 2021, doi: 10.1111/nph.17575.

[85] D. Du *et al.*, "FRIZZY PANICLE defines a regulatory hub for simultaneously controlling spikelet formation

and awn elongation in bread wheat," *New Phytol.*, vol. 231, no. 2, 2021, doi: 10.1111/nph.17388.

[86] N. Budhagatapalli, T. Halbach, S. Hiekel, H. Büchner, A. E. Müller, and J. Kumlehn, "Site-directed mutagenesis in bread and durum wheat via pollination by cas9/guide RNA-transgenic maize used as haploidy inducer," *Plant Biotechnol. J.*, vol. 18, no. 12, 2020, doi: 10.1111/pbi.13415.

[87] T. Li *et al.*, "Highly efficient heritable genome editing in wheat using an RNA virus and bypassing tissue culture," *Mol. Plant*, vol. 14, no. 11, pp. 1787–1798, Nov. 2021, doi: 10.1016/J.MOLP.2021.07.010.

[88] Y. Kumagai *et al.*, "Introduction of a second 'Green Revolution' mutation into wheat via in planta CRISPR/Cas9 delivery," *Plant Physiology*, vol. 188, no. 4. 2022, doi: 10.1093/plphys/kiab570.

[89] J. Hu *et al.*, "Functional analysis of TaPDI genes on storage protein accumulation by CRISPR/Cas9 edited wheat mutants," *Int. J. Biol. Macromol.*, vol. 196, 2022, doi: 10.1016/j.ijbiomac.2021.12.048.

[90] X. Tian *et al.*, "Stress granule-associated TaMBF1c confers thermotolerance through regulating specific mRNA translation in wheat (Triticum aestivum)," *New Phytol.*, vol. 233, no. 4, 2022, doi: 10.1111/nph.17865.

[91] Z. Chen *et al.*, "A single nucleotide deletion in the third exon of FT-D1 increases the spikelet number and delays heading date in wheat (Triticum aestivum L.)," *Plant Biotechnol. J.*, vol. 20, no. 5, 2022, doi: 10.1111/pbi.13773.

[92] H. Chen *et al.*, "Development and optimization of a Barley stripe mosaic virus-mediated gene editing system to improve Fusarium head blight resistance in wheat," *Plant Biotechnol. J.*, vol. 20, no. 6, 2022, doi: 10.1111/pbi.13819.

[93] G. Sun *et al.*, "Matrilineal empowers wheat pollen with haploid induction potency by triggering postmitosis reactive oxygen species activity," *New Phytol.*, vol. 233, no. 6, 2022, doi: 10.1111/nph.17963.

[94] T. Li, B. Liu, M. H. Spalding, D. P. Weeks, and B. Yang, "High-efficiency TALEN-based gene editing produces disease-resistant rice," *Nat. Biotechnol.*, 2012, doi: 10.1038/nbt.2199.

[95] Y. Y. Zhang *et al.*, "Rapid and Efficient Gene Modification in Rice and Brachypodium Using TALENs," *Mol. Plant*, vol. 6, no. 4, pp. 1365–1368, Jul. 2013, doi: 10.1093/MP/SSS162.

[96] Z. Feng *et al.*, "Efficient genome editing in plants using a CRISPR/Cas system," *Nat. Publ. Gr.*, 2013, doi: 10.1038/cr.2013.114.

[97] J. Miao *et al.*, "Targeted mutagenesis in rice using CRISPR-Cas system," *Cell Res.*, vol. 23, no. 10, pp. 1233–1236, 2013, doi: 10.1038/cr.2013.123.

[98] Q. Shan *et al.*, "Targeted genome modification of crop plants using a CRISPR-Cas system," *Nat. Biotechnol.*, vol. 31, no. 8, pp. 686–688, 2013, doi: 10.1038/nbt.2650.

[99] H. Zhou, B. Liu, D. P. Weeks, M. H. Spalding, and B. Yang, "Large chromosomal deletions and heritable small genetic changes induced by CRISPR/Cas9 in rice," *Nucleic Acids Res.*, vol. 42, no. 17, pp. 10903–10914, Sep. 2014, doi: 10.1093/nar/gku806.

[100] Q. Shan, Y. Wang, J. Li, and C. Gao, "Genome editing in rice and wheat using the CRISPR/Cas system," *Nat. Protoc.*, vol. 9, no. 10, pp. 2395–2410, 2014, doi: 10.1038/nprot.2014.157.

[101] H. Zhang *et al.*, “The CRISPR/Cas9 system produces specific and homozygous targeted gene editing in rice in one generation,” *Plant Biotechnol. J.*, vol. 12, no. 6, pp. 797–807, Aug. 2014, doi: https://doi.org/10.1111/pbi.12200.

[102] R. Xu *et al.*, “Gene targeting using the Agrobacterium tumefaciens-mediated CRISPR-Cas system in rice,” *Rice*, vol. 7, no. 1, p. 5, 2014, doi: 10.1186/s12284-014-0005-6.

[103] T. Li, B. Liu, C. Y. Chen, and B. Yang, “TALEN utilization in rice genome modifications,” *Methods*, vol. 69, no. 1, pp. 9–16, 2014, doi: https://doi.org/10.1016/j.ymeth.2014.03.019.

[104] T. Ikeda, W. Tanaka, M. Mikami, M. Endo, and H.-Y. Hirano, “Generation of artificial *drooping leaf* mutants by CRISPR-Cas9 technology in rice,” *Genes Genet. Syst.*, vol. 90, no. 4, pp. 231–235, 2015, doi: 10.1266/ggs.15-00030.

[105] M. Wang *et al.*, “Gene Editing by Co-Transformation of TALEN and Chimeric RNA/DNA Oligonucleotides on the Rice OsEPSPS Gene and the Inheritance of Mutations,” *PLoS One*, vol. 10, no. 4, p. e0122755, Apr. 2015, [Online]. Available: https://doi.org/10.1371/journal.pone.0122755.

[106] C. Wang, L. Shen, Y. Fu, C. Yan, and K. Wang, “A Simple CRISPR/Cas9 System for Multiplex Genome Editing in Rice,” *J. Genet. Genomics*, vol. 42, no. 12, pp. 703–706, Dec. 2015, doi: 10.1016/J.JGG.2015.09.011.

[107] J. Zhou *et al.*, “Gene targeting by the TAL effector PthXo2 reveals cryptic resistance gene for bacterial blight of rice,” *Plant J.*, vol. 82, no. 4, pp. 632–643, May 2015, doi: https://doi.org/10.1111/tpj.12838.

[108] Q. Shan, Y. Zhang, K. Chen, K. Zhang, and C. Gao, “Creation of fragrant rice by targeted knockout of the OsBADH2 gene using TALEN technology,” *Plant Biotechnol. J.*, vol. 13, no. 6, pp. 791–800, Aug. 2015, doi: https://doi.org/10.1111/pbi.12312.

[109] L. Ma *et al.*, “TALEN-Based Mutagenesis of Lipoxygenase LOX3 Enhances the Storage Tolerance of Rice (Oryza sativa) Seeds,” *PLoS One*, vol. 10, no. 12, p. e0143877, Dec. 2015, [Online]. Available: https://doi.org/10.1371/journal.pone.0143877.

[110] L. G. Lowder *et al.*, “A CRISPR/Cas9 Toolbox for Multiplexed Plant Genome Editing and Transcriptional Regulation ,” *Plant Physiol.*, vol. 169, no. 2, pp. 971–985, Oct. 2015, doi: 10.1104/pp.15.00636.

[111] R.-F. Xu *et al.*, “Generation of inheritable and ‘transgene clean’ targeted genome-modified rice in later generations using the CRISPR/Cas9 system,” *Sci. Rep.*, vol. 5, no. 1, p. 11491, 2015, doi: 10.1038/srep11491.

[112] X. Ma *et al.*, “A Robust CRISPR/Cas9 System for Convenient, High-Efficiency Multiplex Genome Editing in Monocot and Dicot Plants,” *Mol. Plant*, vol. 8, no. 8, 2015, doi: 10.1016/j.molp.2015.04.007.

[113] K. Xie, B. Minkenberg, and Y. Yang, “Boosting CRISPR/Cas9 multiplex editing capability with the endogenous tRNA-processing system,” *Proc. Natl. Acad. Sci.*, vol. 112, no. 11, pp. 3570 LP – 3575, Mar. 2015, doi: 10.1073/pnas.1420294112.

[114] M. Mikami, S. Toki, and M. Endo, “Comparison of CRISPR/Cas9 expression constructs for efficient targeted mutagenesis in rice,” *Plant Mol. Biol.*, vol. 88, no. 6, pp. 561–572, 2015, doi: 10.1007/s11103-015-0342-x.

[115] R. Xu *et al.*, "Rapid improvement of grain weight via highly efficient CRISPR/Cas9-mediated multiplex genome editing in rice," *J. Genet. Genomics*, vol. 43, no. 8, pp. 529–532, 2016, doi: https://doi.org/10.1016/j.jgg.2016.07.003.

[116] A. Endo, M. Masafumi, H. Kaya, and S. Toki, "Efficient targeted mutagenesis of rice and tobacco genomes using Cpf1 from Francisella novicida," *Sci. Rep.*, vol. 6, no. 1, p. 38169, 2016, doi: 10.1038/srep38169.

[117] F. Wang *et al.*, "Enhanced Rice Blast Resistance by CRISPR/Cas9-Targeted Mutagenesis of the ERF Transcription Factor Gene OsERF922," *PLoS One*, vol. 11, no. 4, p. e0154027, Apr. 2016, [Online]. Available: https://doi.org/10.1371/journal.pone.0154027.

[118] M. Li *et al.*, "Reassessment of the Four Yield-related Genes Gn1a, DEP1, GS3, and IPA1 in Rice Using a CRISPR/Cas9 System ," *Frontiers in Plant Science* , vol. 7. p. 377, 2016, [Online]. Available: https://www.frontiersin.org/article/10.3389/fpls.2016.00377.

[119] X. Tang *et al.*, "A Single Transcript CRISPR-Cas9 System for Efficient Genome Editing in Plants," *Mol. Plant*, vol. 9, no. 7, pp. 1088–1091, Jul. 2016, doi: 10.1016/J.MOLP.2016.05.001.

[120] H. Zhang *et al.*, "TALEN-mediated targeted mutagenesis produces a large variety of heritable mutations in rice," *Plant Biotechnol. J.*, vol. 14, no. 1, pp. 186–194, Jan. 2016, doi: https://doi.org/10.1111/pbi.12372.

[121] T. Li, B. Liu, C. Y. Chen, and B. Yang, "TALEN-Mediated Homologous Recombination Produces Site-Directed DNA Base Change and Herbicide-Resistant Rice," *J. Genet. Genomics*, vol. 43, no. 5, pp. 297–305, May 2016, doi: 10.1016/J.JGG.2016.03.005.

[122] G. Liang, H. Zhang, D. Lou, and D. Yu, "Selection of highly efficient sgRNAs for CRISPR/Cas9-based plant genome editing," *Sci. Rep.*, vol. 6, no. 1, p. 21451, 2016, doi: 10.1038/srep21451.

[123] X. Hu, C. Wang, Y. Fu, Q. Liu, X. Jiao, and K. Wang, "Expanding the Range of CRISPR/Cas9 Genome Editing in Rice," *Mol. Plant*, vol. 9, no. 6, pp. 943–945, 2016, doi: https://doi.org/10.1016/j.molp.2016.03.003.

[124] M. Mikami, S. Toki, and M. Endo, "Precision Targeted Mutagenesis via Cas9 Paired Nickases in Rice," *Plant Cell Physiol.*, vol. 57, no. 5, pp. 1058–1068, May 2016, doi: 10.1093/pcp/pcw049.

[125] G. Jang *et al.*, "Genetic chimerism of CRISPR/Cas9-mediated rice mutants," *Plant Biotechnol. Rep.*, vol. 10, no. 6, pp. 425–435, 2016, doi: 10.1007/s11816-016-0414-7.

[126] Y. Sun *et al.*, "Engineering Herbicide-Resistant Rice Plants through CRISPR/Cas9-Mediated Homologous Recombination of Acetolactate Synthase," *Mol. Plant*, vol. 9, no. 4, pp. 628–631, Apr. 2016, doi: 10.1016/J.MOLP.2016.01.001.

[127] J. Li *et al.*, "Gene replacements and insertions in rice by intron targeting using CRISPR–Cas9," *Nat. Plants*, vol. 2, no. 10, p. 16139, Oct. 2016, doi: 10.1038/nplants.2016.139.

[128] H. Zhou *et al.*, "Development of Commercial Thermo-sensitive Genic Male Sterile Rice Accelerates Hybrid Rice Breeding Using the CRISPR/Cas9-mediated TMS5 Editing System," *Sci. Rep.*, vol. 6, no. 1, p. 37395, 2016, doi: 10.1038/srep37395.

[129] Y. Hiei and T. Komari, "Improved protocols for transformation of indica rice mediated by Agrobacterium tumefaciens," *Plant Cell. Tissue Organ Cult.*, vol. 85, no. 3, p. 271, 2006, doi: 10.1007/s11240-005-9069-8.

[130] T. Ishizaki, "CRISPR/Cas9 in rice can induce new mutations in later generations, leading to chimerism and unpredicted segregation of the targeted mutation," *Mol. Breed.*, vol. 36, no. 12, p. 165, 2016, doi: 10.1007/s11032-016-0591-7.

[131] L. Wu *et al.*, "The Rice Pentatricopeptide Repeat Gene TCD10 is Needed for Chloroplast Development under Cold Stress," *Rice*, vol. 9, no. 1, p. 67, 2016, doi: 10.1186/s12284-016-0134-1.

[132] C. Baysal, L. Bortesi, C. Zhu, G. Farré, S. Schillberg, and P. Christou, "CRISPR/Cas9 activity in the rice OsBEIIb gene does not induce off-target effects in the closely related paralog OsBEIIa," *Mol. Breed.*, vol. 36, no. 8, p. 108, 2016, doi: 10.1007/s11032-016-0533-4.

[133] Q. Li *et al.*, "Development of japonica Photo-Sensitive Genic Male Sterile Rice Lines by Editing Carbon Starved Anther Using CRISPR/Cas9," *J. Genet. Genomics*, vol. 43, no. 6, pp. 415–419, Jun. 2016, doi: 10.1016/J.JGG.2016.04.011.

[134] B. Ren *et al.*, "A CRISPR/Cas9 toolkit for efficient targeted base editing to induce genetic variations in rice," *Sci. China Life Sci.*, vol. 60, no. 5, pp. 516–519, 2017, doi: 10.1007/s11427-016-0406-x.

[135] X. Tang *et al.*, "A CRISPR–Cpf1 system for efficient genome editing and transcriptional repression in plants," *Nat. Plants*, vol. 3, no. 3, p. 17018, 2017, doi: 10.1038/nplants.2017.18.

[136] Y. Lu and J. K. Zhu, "Precise Editing of a Target Base in the Rice Genome Using a Modified CRISPR/Cas9 System," *Mol. Plant*, vol. 10, no. 3, pp. 523–525, Mar. 2017, doi: 10.1016/J.MOLP.2016.11.013.

[137] D. Lou, H. Wang, G. Liang, and D. Yu, "OsSAPK2 Confers Abscisic Acid Sensitivity and Tolerance to Drought Stress in Rice ," *Frontiers in Plant Science* , vol. 8. p. 993, 2017, [Online]. Available: https://www.frontiersin.org/article/10.3389/fpls.2017.00993.

[138] Y. Sun *et al.*, "Generation of High-Amylose Rice through CRISPR/Cas9-Mediated Targeted Mutagenesis of Starch Branching Enzymes ," *Frontiers in Plant Science* , vol. 8. p. 298, 2017, [Online]. Available: https://www.frontiersin.org/article/10.3389/fpls.2017.00298.

[139] L. Tang *et al.*, "Knockout of OsNramp5 using the CRISPR/Cas9 system produces low Cd-accumulating indica rice without compromising yield," *Sci. Rep.*, vol. 7, no. 1, p. 14438, 2017, doi: 10.1038/s41598-017-14832-9.

[140] L. Cai *et al.*, "A Transcription Activator-Like Effector Tal7 of Xanthomonas oryzae pv. oryzicola Activates Rice Gene Os09g29100 to Suppress Rice Immunity," *Sci. Rep.*, vol. 7, no. 1, p. 5089, 2017, doi: 10.1038/s41598-017-04800-8.

[141] X. Li *et al.*, "High-efficiency breeding of early-maturing rice cultivars via CRISPR/Cas9-mediated genome editing," *J. Genet. Genomics*, vol. 44, no. 3, pp. 175–178, Mar. 2017, doi: 10.1016/J.JGG.2017.02.001.

[142] C. Shen *et al.*, "Knock out of the annexin gene OsAnn3 via CRISPR/Cas9-mediated genome editing decreased cold tolerance in rice," *J. Plant Biol.*, vol. 60, no. 6, pp. 539–547, 2017, doi: 10.1007/s12374-016-0400-1.

[143] Z. Shimatani *et al.*, “Targeted base editing in rice and tomato using a CRISPR-Cas9 cytidine deaminase fusion,” *Nat. Biotechnol.*, vol. 35, no. 5, pp. 441–443, May 2017, doi: 10.1038/NBT.3833.

[144] S. Blanvillain-Baufumé *et al.*, “Targeted promoter editing for rice resistance to Xanthomonas oryzae pv. oryzae reveals differential activities for SWEET14-inducing TAL effectors,” *Plant Biotechnol. J.*, vol. 15, no. 3, pp. 306–317, Mar. 2017, doi: https://doi.org/10.1111/pbi.12613.

[145] B. Minkenberg, K. Xie, and Y. Yang, “Discovery of rice essential genes by characterizing a CRISPR-edited mutation of closely related rice MAP kinase genes,” *Plant J.*, vol. 89, no. 3, pp. 636–648, Feb. 2017, doi: https://doi.org/10.1111/tpj.13399.

[146] H.-P. Lu *et al.*, “CRISPR-S: an active interference element for a rapid and inexpensive selection of genome-edited, transgene-free rice plants,” *Plant Biotechnol. J.*, vol. 15, no. 11, pp. 1371–1373, Nov. 2017, doi: https://doi.org/10.1111/pbi.12788.

[147] R. F. Xu *et al.*, “Generation of targeted mutant rice using a CRISPR-Cpf1 system,” *PLANT Biotechnol. J.*, vol. 15, no. 6, pp. 713–717, 2017, doi: 10.1111/pbi.12669.

[148] Y. Zong *et al.*, “Precise base editing in rice, wheat and maize with a Cas9-cytidine deaminase fusion,” *Nat. Biotechnol.*, vol. 35, no. 5, pp. 438–440, May 2017, doi: 10.1038/NBT.3811.

[149] J. Li, Y. Sun, J. Du, Y. Zhao, and L. Xia, “Generation of Targeted Point Mutations in Rice by a Modified CRISPR/Cas9 System,” *Mol. Plant*, vol. 10, no. 3, pp. 526–529, Mar. 2017, doi: 10.1016/J.MOLP.2016.12.001.

[150] M. B. Begemann *et al.*, “Precise insertion and guided editing of higher plant genomes using Cpf1 CRISPR nucleases,” *Sci. Rep.*, vol. 7, no. 1, p. 11606, 2017, doi: 10.1038/s41598-017-11760-6.

[151] J. Yu *et al.*, “Two rice receptor-like kinases maintain male fertility under changing temperatures,” *Proc. Natl. Acad. Sci.*, vol. 114, no. 46, pp. 12327–12332, Nov. 2017, doi: 10.1073/pnas.1705189114.

[152] X. Yin *et al.*, “CRISPR-Cas9 and CRISPR-Cpf1 mediated targeting of a stomatal developmental gene EPFL9 in rice,” *Plant Cell Rep.*, vol. 36, no. 5, pp. 745–757, 2017, doi: 10.1007/s00299-017-2118-z.

[153] J. Zhou *et al.*, “CRISPR-Cas9 Based Genome Editing Reveals New Insights into MicroRNA Function and Regulation in Rice ,” *Frontiers in Plant Science* , vol. 8. p. 1598, 2017, [Online]. Available: https://www.frontiersin.org/article/10.3389/fpls.2017.01598.

[154] M. Wang, Y. Mao, Y. Lu, X. Tao, and J. kang Zhu, “Multiplex Gene Editing in Rice Using the CRISPR-Cpf1 System,” *Mol. Plant*, vol. 10, no. 7, pp. 1011–1013, Jul. 2017, doi: 10.1016/J.MOLP.2017.03.001.

[155] L. Shen *et al.*, “Rapid generation of genetic diversity by multiplex CRISPR/Cas9 genome editing in rice,” *Sci. China Life Sci.*, vol. 60, no. 5, 2017, doi: 10.1007/s11427-017-9008-8.

[156] H. Butt *et al.*, “Efficient CRISPR/Cas9-Mediated Genome Editing Using a Chimeric Single-Guide RNA Molecule ,” *Frontiers in Plant Science* , vol. 8. 2017, [Online]. Available: https://www.frontiersin.org/article/10.3389/fpls.2017.01441.

[157] X. Yang, L. Chen, J. He, and W. Yu, “Knocking out of carotenoid catabolic genes in rice fails to boost carotenoid accumulation, but reveals a mutation in strigolactone biosynthesis,” *Plant Cell Rep.*, vol. 36, no. 10, pp. 1533–1545, 2017, doi: 10.1007/s00299-017-2172-6.

[158] X. Meng *et al.*, “Construction of a Genome-Wide Mutant Library in Rice Using CRISPR/Cas9,” *Mol. Plant*, vol. 10, no. 9, pp. 1238–1241, 2017, doi: https://doi.org/10.1016/j.molp.2017.06.006.

[159] F. WANG *et al.*, “Creation of gene-specific rice mutants by AvrXa23-based TALENs,” *J. Integr. Agric.*, vol. 16, no. 2, pp. 424–434, 2017, doi: https://doi.org/10.1016/S2095-3119(16)61411-9.

[160] Y. Xie *et al.*, “Suppression or knockout of SaF/SaM overcomes the Sa-mediated hybrid male sterility in rice,” *J. Integr. Plant Biol.*, vol. 59, no. 9, pp. 669–679, Sep. 2017, doi: https://doi.org/10.1111/jipb.12564.

[161] X. Tang *et al.*, “A large-scale whole-genome sequencing analysis reveals highly specific genome editing by both Cas9 and Cpf1 (Cas12a) nucleases in rice,” *Genome Biol.*, vol. 19, no. 1, p. 84, 2018, doi: 10.1186/s13059-018-1458-5.

[162] X. Meng *et al.*, “Robust genome editing of CRISPR-Cas9 at NAG PAMs in rice,” *Sci. China Life Sci.*, vol. 61, no. 1, pp. 122–125, 2018, doi: 10.1007/s11427-017-9247-9.

[163] L. Shen *et al.*, “QTL editing confers opposing yield performance in different rice varieties,” *J. Integr. Plant Biol.*, vol. 60, no. 2, pp. 89–93, Feb. 2018, doi: https://doi.org/10.1111/jipb.12501.

[164] D. Ding, K. Chen, Y. Chen, H. Li, and K. Xie, “Engineering Introns to Express RNA Guides for Cas9- and Cpf1-Mediated Multiplex Genome Editing,” *Mol. Plant*, vol. 11, no. 4, pp. 542–552, 2018, doi: https://doi.org/10.1016/j.molp.2018.02.005.

[165] S. Li *et al.*, “Expanding the Scope of CRISPR/Cpf1-Mediated Genome Editing in Rice,” *Mol. Plant*, vol. 11, no. 7, pp. 995–998, Jul. 2018, doi: 10.1016/J.MOLP.2018.03.009.

[166] X. Hu, X. Meng, Q. Liu, J. Li, and K. Wang, “Increasing the efficiency of CRISPR-Cas9-VQR precise genome editing in rice,” *Plant Biotechnol. J.*, vol. 16, no. 1, 2018, doi: 10.1111/pbi.12771.

[167] L. Huang *et al.*, “Developing superior alleles of yield genes in rice by artificial mutagenesis using the CRISPR/Cas9 system,” *Crop J.*, vol. 6, no. 5, pp. 475–481, Oct. 2018, doi: https://doi.org/10.1016/j.cj.2018.05.005.

[168] S. Li *et al.*, “Synthesis-dependent repair of Cpf1-induced double strand DNA breaks enables targeted gene replacement in rice,” *J. Exp. Bot.*, vol. 69, no. 20, pp. 4715–4721, Sep. 2018, doi: 10.1093/jxb/ery245.

[169] Y. Zong *et al.*, “Efficient C-to-T base editing in plants using a fusion of nCas9 and human APOBEC3A,” *Nat. Biotechnol.*, vol. 36, no. 10, pp. 950–953, 2018, doi: 10.1038/nbt.4261.

[170] Y.-J. Jung, F. M. Nogoy, S.-K. Lee, Y.-G. Cho, and K.-K. Kang, “Application of ZFN for Site Directed Mutagenesis of Rice SSIVa Gene,” *Biotechnol. Bioprocess Eng.*, vol. 23, no. 1, pp. 108–115, 2018, doi: 10.1007/s12257-017-0420-9.

[171] Z. Shimatani *et al.*, “Inheritance of co-edited genes by CRISPR-based targeted nucleotide substitutions in rice,” *Plant Physiol. Biochem.*, vol. 131, pp. 78–83, 2018, doi: https://doi.org/10.1016/j.plaphy.2018.04.028.

[172] H. Butt, M. Jamil, J. Y. Wang, S. Al-Babili, and M. Mahfouz, “Engineering plant architecture via CRISPR/Cas9-mediated alteration of strigolactone biosynthesis,” *BMC Plant Biol.*, vol. 18, no. 1, p. 174,

2018, doi: 10.1186/s12870-018-1387-1.

[173] Z. Zhong *et al.*, "Plant Genome Editing Using FnCpf1 and LbCpf1 Nucleases at Redefined and Altered PAM Sites," *Mol. Plant*, vol. 11, no. 7, pp. 999–1002, Jul. 2018, doi: 10.1016/J.MOLP.2018.03.008.

[174] M. Wang, Y. Mao, Y. Lu, Z. Wang, X. Tao, and J.-K. Zhu, "Multiplex gene editing in rice with simplified CRISPR-Cpf1 and CRISPR-Cas9 systems," *J. Integr. Plant Biol.*, vol. 60, no. 8, pp. 626–631, Aug. 2018, doi: https://doi.org/10.1111/jipb.12667.

[175] B. Ren *et al.*, "Improved Base Editor for Efficiently Inducing Genetic Variations in Rice with CRISPR/Cas9-Guided Hyperactive hAID Mutant," *Mol. Plant*, vol. 11, no. 4, pp. 623–626, Apr. 2018, doi: 10.1016/J.MOLP.2018.01.005.

[176] H. Onodera *et al.*, "Establishment of a conditional TALEN system using the translational enhancer dMac3 and an inducible promoter activated by glucocorticoid treatment to increase the frequency of targeted mutagenesis in plants," *PLoS One*, vol. 13, no. 12, p. e0208959, Dec. 2018, [Online]. Available: https://doi.org/10.1371/journal.pone.0208959.

[177] C. Miao *et al.*, "Mutations in a subfamily of abscisic acid receptor genes promote rice growth and productivity," *Proc. Natl. Acad. Sci.*, vol. 115, no. 23, pp. 6058 LP – 6063, Jun. 2018, doi: 10.1073/pnas.1804774115.

[178] K. Abe, E. Araki, Y. Suzuki, S. Toki, and H. Saika, "Production of high oleic/low linoleic rice by genome editing," *Plant Physiol. Biochem.*, vol. 131, pp. 58–62, Oct. 2018, doi: 10.1016/J.PLAPHY.2018.04.033.

[179] K. Hua, X. Tao, F. Yuan, D. Wang, and J. K. Zhu, "Precise A·T to G·C Base Editing in the Rice Genome," *Mol. Plant*, vol. 11, no. 4, pp. 627–630, Apr. 2018, doi: 10.1016/J.MOLP.2018.02.007.

[180] F. Yan *et al.*, "Highly Efficient A·T to G·C Base Editing by Cas9n-Guided tRNA Adenosine Deaminase in Rice," *Mol. Plant*, vol. 11, no. 4, pp. 631–634, Apr. 2018, doi: 10.1016/J.MOLP.2018.02.008.

[181] A. Macovei *et al.*, "Novel alleles of rice eIF4G generated by CRISPR/Cas9-targeted mutagenesis confer resistance to Rice tungro spherical virus," *Plant Biotechnol. J.*, vol. 16, no. 11, pp. 1918–1927, Nov. 2018, doi: 10.1111/PBI.12927.

[182] P. Rajesh Shah, S. Varanavasiappan, E. Kokiladevi, A. Ramanathan, and K. K. Kumar, "Genome Editing of Rice PFT1 Gene to Study its Role in Rice Sheath Blight Disease Resistance," *Int. J. Curr. Microbiol. Appl. Sci.*, vol. 8, no. 06, 2019, doi: 10.20546/ijcmas.2019.806.281.

[183] I. Khanday, D. Skinner, B. Yang, R. Mercier, and V. Sundaresan, "A male-expressed rice embryogenic trigger redirected for asexual propagation through seeds," *Nature*, vol. 565, no. 7737, pp. 91–95, 2019, doi: 10.1038/s41586-018-0785-8.

[184] X. Yin, A. Anand, P. Quick, and A. Bandyopadhyay, "Editing a Stomatal Developmental Gene in Rice with CRISPR/Cpf1 BT - Plant Genome Editing with CRISPR Systems: Methods and Protocols," Y. Qi, Ed. New York, NY: Springer New York, 2019, pp. 257–268.

[185] C. Wang *et al.*, "Clonal seeds from hybrid rice by simultaneous genome engineering of meiosis and fertilization genes," *Nat. Biotechnol.*, vol. 37, no. 3, pp. 283–286, 2019, doi: 10.1038/s41587-018-0003-0.

[186] H. Butt *et al.*, "CRISPR directed evolution of the spliceosome for resistance to splicing inhibitors," *Genome Biol.*, vol. 20, no. 1, p. 73, 2019, doi: 10.1186/s13059-019-1680-9.

[187] T. Miyamoto *et al.*, "OsMYB108 loss-of-function enriches p-coumaroylated and tricin lignin units in rice cell walls," *Plant J.*, vol. 98, no. 6, pp. 975–987, Jun. 2019, doi: https://doi.org/10.1111/tpj.14290.

[188] L. Hao *et al.*, "CRISPR/Cas9-Mediated Adenine Base Editing in Rice Genome," *Rice Science*, vol. 26, no. 2. 2019, doi: 10.1016/j.rsci.2018.07.002.

[189] R. Oliva *et al.*, "Broad-spectrum resistance to bacterial blight in rice using genome editing," *Nat. Biotechnol.*, vol. 37, no. 11, 2019, doi: 10.1038/s41587-019-0267-z.

[190] M. Jiang, Y. Liu, Y. Liu, Y. Tan, J. Huang, and Q. Shu, "Mutation of Inositol 1,3,4-trisphosphate 5/6-kinase6 Impairs Plant Growth and Phytic Acid Synthesis in Rice," *Plants*, vol. 8, no. 5. 2019, doi: 10.3390/plants8050114.

[191] Q. Ren *et al.*, "Bidirectional Promoter-Based CRISPR-Cas9 Systems for Plant Genome Editing ," *Frontiers in Plant Science* , vol. 10. p. 1173, 2019, [Online]. Available: https://www.frontiersin.org/article/10.3389/fpls.2019.01173.

[192] Z. Zhong *et al.*, "Improving Plant Genome Editing with High-Fidelity xCas9 and Non-canonical PAM-Targeting Cas9-NG," *Mol. Plant*, vol. 12, no. 7, pp. 1027–1036, Jul. 2019, doi: 10.1016/J.MOLP.2019.03.011.

[193] B. Ren *et al.*, "Cas9-NG Greatly Expands the Targeting Scope of the Genome-Editing Toolkit by Recognizing NG and Other Atypical PAMs in Rice," *Mol. Plant*, vol. 12, no. 7, pp. 1015–1026, Jul. 2019, doi: 10.1016/J.MOLP.2019.03.010.

[194] K. Hua, X. Tao, and J.-K. K. Zhu, "Expanding the base editing scope in rice by using Cas9 variants," *Plant Biotechnol. J.*, vol. 17, no. 2, pp. 499–504, Feb. 2019, doi: https://doi.org/10.1111/pbi.12993.

[195] X. Tang *et al.*, "Single transcript unit CRISPR 2.0 systems for robust Cas9 and Cas12a mediated plant genome editing," *Plant Biotechnol. J.*, vol. 17, no. 7, pp. 1431–1445, Jul. 2019, doi: https://doi.org/10.1111/pbi.13068.

[196] N. Aliaga-Franco *et al.*, "Identification of Transgene-Free CRISPR-Edited Plants of Rice, Tomato, and Arabidopsis by Monitoring DsRED Fluorescence in Dry Seeds ," *Frontiers in Plant Science* , vol. 10. 2019, [Online]. Available: https://www.frontiersin.org/articles/10.3389/fpls.2019.01150.

[197] Y. Takeda *et al.*, "Lignin characterization of rice CONIFERALDEHYDE 5-HYDROXYLASE loss-of-function mutants generated with the CRISPR/Cas9 system," *Plant J.*, vol. 97, no. 3, pp. 543–554, Feb. 2019, doi: https://doi.org/10.1111/tpj.14141.

[198] H. Saika, A. Mori, M. Endo, and S. Toki, "Targeted deletion of rice retrotransposon Tos17 via CRISPR/Cas9," *Plant Cell Rep.*, vol. 38, no. 4, pp. 455–458, 2019, doi: 10.1007/s00299-018-2357-7.

[199] R. Qin *et al.*, "Developing a highly efficient and wildly adaptive CRISPR-SaCas9 toolset for plant genome editing," *Plant Biotechnol. J.*, vol. 17, no. 4, pp. 706–708, Apr. 2019, doi: https://doi.org/10.1111/pbi.13047.

[200] A. Zhang *et al.*, "Enhanced rice salinity tolerance via CRISPR/Cas9-targeted mutagenesis of the OsRR22

gene," *Mol. Breed.*, vol. 39, no. 3, p. 47, 2019, doi: 10.1007/s11032-019-0954-y.

[201] S. Lan *et al.*, "Rapid Creation of New Photoperiod-/Thermo-Sensitive Genic Male-Sterile Rice Materials by CRISPR/Cas9 System," *Rice Sci.*, vol. 26, no. 2, pp. 129–132, 2019, doi: https://doi.org/10.1016/j.rsci.2018.12.006.

[202] A. Endo, H. Saika, M. Takemura, N. Misawa, and S. Toki, "A novel approach to carotenoid accumulation in rice callus by mimicking the cauliflower Orange mutation via genome editing," *Rice*, vol. 12, no. 1, p. 81, 2019, doi: 10.1186/s12284-019-0345-3.

[203] K. Hua, X. Tao, P. Han, R. Wang, and J. K. Zhu, "Genome Engineering in Rice Using Cas9 Variants that Recognize NG PAM Sequences," *Mol. Plant*, vol. 12, no. 7, 2019, doi: 10.1016/j.molp.2019.03.009.

[204] R. Xu, R. Qin, H. Li, J. Li, J. Yang, and P. Wei, "Enhanced genome editing in rice using single transcript unit CRISPR-LbCpf1 systems," *Plant Biotechnology Journal*, vol. 17, no. 3. 2019, doi: 10.1111/pbi.13028.

[205] Z. Wu *et al.*, "Multiple transcriptional factors control stomata development in rice," *New Phytol.*, vol. 223, no. 1, pp. 220–232, Jul. 2019, doi: https://doi.org/10.1111/nph.15766.

[206] L. Songmei *et al.*, "Characterization and Evaluation of OsLCT1 and OsNramp5 Mutants Generated Through CRISPR/Cas9-Mediated Mutagenesis for Breeding Low Cd Rice," *Rice Sci.*, vol. 26, no. 2, pp. 88–97, 2019, doi: https://doi.org/10.1016/j.rsci.2019.01.002.

[207] J. Wang *et al.*, "xCas9 expands the scope of genome editing with reduced efficiency in rice," *Plant Biotechnology Journal*, vol. 17, no. 4. 2019, doi: 10.1111/pbi.13053.

[208] S. Li *et al.*, "Developing disease-resistant thermosensitive male sterile rice by multiplex gene editing," *J. Integr. Plant Biol.*, vol. 61, no. 12, pp. 1201–1205, Dec. 2019, doi: https://doi.org/10.1111/jipb.12774.

[209] X. Wang *et al.*, "Disruption of an amino acid transporter LHT1 leads to growth inhibition and low yields in rice," *BMC Plant Biol.*, vol. 19, no. 1, p. 268, 2019, doi: 10.1186/s12870-019-1885-9.

[210] Y. Cui, M. Zhu, Z. Xu, and Q. Xu, "Assessment of the effect of ten heading time genes on reproductive transition and yield components in rice using a CRISPR/Cas9 system," *Theor. Appl. Genet.*, vol. 132, no. 6, pp. 1887–1896, 2019, doi: 10.1007/s00122-019-03324-1.

[211] H. Yubing *et al.*, "Improvements of TKC Technology Accelerate Isolation of Transgene-Free CRISPR/Cas9-Edited Rice Plants," *Rice Sci.*, vol. 26, no. 2, pp. 109–117, 2019, doi: https://doi.org/10.1016/j.rsci.2018.11.001.

[212] A. A. Malzahn *et al.*, "Application of CRISPR-Cas12a temperature sensitivity for improved genome editing in rice, maize, and Arabidopsis," *BMC Biol.*, vol. 17, no. 1, p. 9, 2019, doi: 10.1186/s12915-019-0629-5.

[213] X. Hu *et al.*, "Using CRISPR-Cas9 to generate semi-dwarf rice lines in elite landraces," *Sci. Rep.*, vol. 9, no. 1, p. 19096, 2019, doi: 10.1038/s41598-019-55757-9.

[214] T. Kazama *et al.*, "Curing cytoplasmic male sterility via TALEN-mediated mitochondrial genome editing," *Nat. Plants*, vol. 5, no. 7, pp. 722–730, 2019, doi: 10.1038/s41477-019-0459-z.

[215] K. Negishi, H. Kaya, K. Abe, N. Hara, H. Saika, and S. Toki, "An adenine base editor with expanded targeting scope using SpCas9-NGv1 in rice," *Plant Biotechnol. J.*, vol. 17, no. 8, pp. 1476–1478, Aug.

2019, doi: https://doi.org/10.1111/pbi.13120.

[216] E. Toda *et al.*, "An efficient DNA- and selectable-marker-free genome-editing system using zygotes in rice," *Nat. Plants*, vol. 5, no. 4, pp. 363–368, 2019, doi: 10.1038/s41477-019-0386-z.

[217] S. Li *et al.*, "Precise gene replacement in rice by RNA transcript-templated homologous recombination," *Nat. Biotechnol. 2019 374*, vol. 37, no. 4, pp. 445–450, Mar. 2019, doi: 10.1038/s41587-019-0065-7.

[218] G. Liu, K. Yin, Q. Zhang, C. Gao, and J.-L. Qiu, "Modulating chromatin accessibility by transactivation and targeting proximal dsgRNAs enhances Cas9 editing efficiency in vivo," *Genome Biol.*, vol. 20, no. 1, p. 145, 2019, doi: 10.1186/s13059-019-1762-8.

[219] L. Pérez *et al.*, "CRISPR/Cas9 mutations in the rice Waxy/GBSSI gene induce allele-specific and zygosity-dependent feedback effects on endosperm starch biosynthesis," *Plant Cell Rep.*, vol. 38, no. 3, pp. 417–433, 2019, doi: 10.1007/s00299-019-02388-z.

[220] H. Y. Lee, Z. Chen, C. Zhang, and G. M. Yoon, "Editing of the OsACS locus alters phosphate deficiency-induced adaptive responses in rice seedlings," *J. Exp. Bot.*, vol. 70, no. 6, pp. 1927–1940, Mar. 2019, doi: 10.1093/jxb/erz074.

[221] Y. Han *et al.*, "Generation of semi-dwarf rice (Oryza sativa L.) lines by CRISPR/Cas9-directed mutagenesis of OsGA20ox2 and proteomic analysis of unveiled changes caused by mutations ," *3 Biotech*, vol. 9, no. 11, p. 387, 2019, doi: 10.1007/s13205-019-1919-x.

[222] Y.-A. Kim, H. Moon, and C.-J. Park, "CRISPR/Cas9-targeted mutagenesis of Os8N3 in rice to confer resistance to Xanthomonas oryzae pv. oryzae," *Rice*, vol. 12, no. 1, p. 67, 2019, doi: 10.1186/s12284-019-0325-7.

[223] R. Banakar *et al.*, "High-frequency random DNA insertions upon co-delivery of CRISPR-Cas9 ribonucleoprotein and selectable marker plasmid in rice," *Sci. Rep.*, vol. 9, no. 1, p. 19902, 2019, doi: 10.1038/s41598-019-55681-y.

[224] S. Liao *et al.*, "CRISPR/Cas9-Induced Mutagenesis of Semi-Rolled Leaf1,2 Confers Curled Leaf Phenotype and Drought Tolerance by Influencing Protein Expression Patterns and ROS Scavenging in Rice (Oryza sativa L.)," *Agronomy*, vol. 9, no. 11. 2019, doi: 10.3390/agronomy9110728.

[225] X. Wang *et al.*, "Breeding of indica glutinous cytoplasmic male sterile line wx209a via crispr/cas9 mediated genomic editing," *Czech J. Genet. Plant Breed.*, vol. 55, no. 3, 2019, doi: 10.17221/197/2017-CJGPB.

[226] J. Zhou *et al.*, "Multiplex QTL editing of grain-related genes improves yield in elite rice varieties," *Plant Cell Rep.*, vol. 38, no. 4, pp. 475–485, 2019, doi: 10.1007/s00299-018-2340-3.

[227] X. Fu *et al.*, "Enhanced Expression of QTL qLL9/DEP1 Facilitates the Improvement of Leaf Morphology and Grain Yield in Rice," *International Journal of Molecular Sciences*, vol. 20, no. 4. 2019, doi: 10.3390/ijms20040866.

[228] Y. J. Jung *et al.*, "Acquisition of seed dormancy breaking in rice (Oryza sativa L.) via CRISPR/Cas9-targeted mutagenesis of OsVP1 gene," *Plant Biotechnol. Rep.*, vol. 13, no. 5, pp. 511–520, 2019, doi: 10.1007/s11816-019-00580-x.

[229] Y. J. Jung *et al.*, "CRISPR/Cas9-targeted mutagenesis of F3'H, DFR and LDOX, genes related to anthocyanin biosynthesis in black rice (Oryza sativa L.)," *Plant Biotechnol. Rep.*, vol. 13, no. 5, pp. 521–531, 2019, doi: 10.1007/s11816-019-00579-4.

[230] R. Cheng, L. Gong, Z. Li, and Y.-K. Liang, "Rice BIG gene is required for seedling viability," *J. Plant Physiol.*, vol. 232, pp. 39–50, 2019, doi: https://doi.org/10.1016/j.jplph.2018.11.006.

[231] Y. Xu *et al.*, "ScCas9 recognizes NNG protospacer adjacent motif in genome editing of rice," *Sci. China Life Sci.*, vol. 63, no. 3, pp. 450–452, 2020, doi: 10.1007/s11427-019-1630-2.

[232] R. Qin *et al.*, "Increasing fidelity and efficiency by modifying cytidine base-editing systems in rice," *Crop J.*, vol. 8, no. 3, pp. 396–402, Jun. 2020, doi: 10.1016/J.CJ.2019.04.007.

[233] Y. Lu *et al.*, "Targeted, efficient sequence insertion and replacement in rice," *Nat. Biotechnol.*, vol. 38, no. 12, pp. 1402–1407, 2020, doi: 10.1038/s41587-020-0581-5.

[234] Y. J. Jung *et al.*, "Generation and Transcriptome Profiling of Slr1-d7 and Slr1-d8 Mutant Lines with a New Semi-Dominant Dwarf Allele of SLR1 Using the CRISPR/Cas9 System in Rice," *International Journal of Molecular Sciences*, vol. 21, no. 15. 2020, doi: 10.3390/ijms21155492.

[235] X. Hu, X. Meng, J. Li, K. Wang, and H. Yu, "Improving the efficiency of the CRISPR-Cas12a system with tRNA-crRNA arrays," *Crop J.*, vol. 8, no. 3, 2020, doi: 10.1016/j.cj.2019.06.007.

[236] A. Komatsu, M. Ohtake, Z. Shimatani, and K. Nishida, "Production of Herbicide-Sensitive Strain to Prevent Volunteer Rice Infestation Using a CRISPR-Cas9 Cytidine Deaminase Fusion ," *Frontiers in Plant Science* , vol. 11. 2020, [Online]. Available: https://www.frontiersin.org/article/10.3389/fpls.2020.00925.

[237] E. Lacchini *et al.*, "CRISPR-mediated accelerated domestication of African rice landraces," *PLoS One*, vol. 15, no. 3, p. e0229782, Mar. 2020, [Online]. Available: https://doi.org/10.1371/journal.pone.0229782.

[238] Y. Xu *et al.*, "Intron-targeted gene insertion in rice using CRISPR/Cas9: A case study of the Pi-ta gene," *Crop J.*, vol. 8, no. 3, pp. 424–431, 2020, doi: https://doi.org/10.1016/j.cj.2019.03.006.

[239] J. Zhang *et al.*, "Disruption of MIR396e and MIR396f improves rice yield under nitrogen-deficient conditions," *Natl. Sci. Rev.*, vol. 7, no. 1, pp. 102–112, Jan. 2020, doi: 10.1093/nsr/nwz142.

[240] M. Wang *et al.*, "Targeted base editing in rice with CRISPR/ScCas9 system," *Plant Biotechnol. J.*, vol. 18, no. 8, pp. 1645–1647, Aug. 2020, doi: https://doi.org/10.1111/pbi.13330.

[241] X. Zheng *et al.*, "The Improvement of CRISPR-Cas9 System With Ubiquitin-Associated Domain Fusion for Efficient Plant Genome Editing," *Front. Plant Sci.*, vol. 11, 2020, doi: 10.3389/fpls.2020.00621.

[242] X. Liu *et al.*, "A simple and efficient cloning system for CRISPR/Cas9-mediated genome editing in rice," *PeerJ*, vol. 2020, no. 1, 2020, doi: 10.7717/peerj.8491.

[243] R. Banakar, M. Schubert, M. Collingwood, C. Vakulskas, A. L. Eggenberger, and K. Wang, "Comparison of CRISPR-Cas9/Cas12a Ribonucleoprotein Complexes for Genome Editing Efficiency in the Rice Phytoene Desaturase (OsPDS) Gene," *Rice*, vol. 13, no. 1, p. 4, 2020, doi: 10.1186/s12284-019-0365-z.

[244] K. Hua, Y. Jiang, X. Tao, and J. K. Zhu, "Precision genome engineering in rice using prime editing system," *Plant Biotechnol. J.*, vol. 18, no. 11, 2020, doi: 10.1111/pbi.13395.

[245] W. Xu *et al.*, “Versatile Nucleotides Substitution in Plant Using an Improved Prime Editing System,” *Mol. Plant*, vol. 13, no. 5, pp. 675–678, May 2020, doi: 10.1016/J.MOLP.2020.03.012.

[246] C. Li *et al.*, “SWISS: multiplexed orthogonal genome editing in plants with a Cas9 nickase and engineered CRISPR RNA scaffolds,” *Genome Biol.*, vol. 21, no. 1, p. 141, 2020, doi: 10.1186/s13059-020-02051-x.

[247] Z. Zhong *et al.*, “Intron-Based Single Transcript Unit CRISPR Systems for Plant Genome Editing,” *Rice*, vol. 13, no. 1, p. 8, 2020, doi: 10.1186/s12284-020-0369-8.

[248] W. Xu *et al.*, “Discriminated sgRNAs-Based SurroGate System Greatly Enhances the Screening Efficiency of Plant Base-Edited Cells,” *Mol. Plant*, vol. 13, no. 1, pp. 169–180, 2020, doi: https://doi.org/10.1016/j.molp.2019.10.007.

[249] D. Zeng *et al.*, “Quantitative regulation of Waxy expression by CRISPR/Cas9-based promoter and 5’UTR-intron editing improves grain quality in rice,” *Plant Biotechnol. J.*, vol. 18, no. 12, pp. 2385–2387, Dec. 2020, doi: https://doi.org/10.1111/pbi.13427.

[250] R. K. Sahoo and N. Tuteja, “Development of Agrobacterium-mediated transformation technology for mature seed-derived callus tissues of indica rice cultivar IR64,” *GM Crops Food*, vol. 3, no. 2, pp. 123–128, Apr. 2012, doi: 10.4161/gmcr.20032.

[251] V. V Santosh Kumar *et al.*, “CRISPR-Cas9 mediated genome editing of drought and salt tolerance (OsDST) gene in indica mega rice cultivar MTU1010,” *Physiol. Mol. Biol. Plants*, vol. 26, no. 6, pp. 1099–1110, 2020, doi: 10.1007/s12298-020-00819-w.

[252] Q. Gao *et al.*, “Targeted Mutagenesis of the Rice FW 2.2-Like Gene Family Using the CRISPR/Cas9 System Reveals OsFWL4 as a Regulator of Tiller Number and Plant Yield in Rice,” *International Journal of Molecular Sciences*, vol. 21, no. 3. 2020, doi: 10.3390/ijms21030809.

[253] Y. Ueda *et al.*, “Gene regulatory network and its constituent transcription factors that control nitrogen-deficiency responses in rice,” *New Phytol.*, vol. 227, no. 5, pp. 1434–1452, Sep. 2020, doi: https://doi.org/10.1111/nph.16627.

[254] K. Zafar *et al.*, “Precise CRISPR-Cas9 Mediated Genome Editing in Super Basmati Rice for Resistance Against Bacterial Blight by Targeting the Major Susceptibility Gene,” *Front. Plant Sci.*, vol. 11, 2020, doi: 10.3389/fpls.2020.00575.

[255] K. Akama, N. Akter, H. Endo, M. Kanesaki, M. Endo, and S. Toki, “An In Vivo Targeted Deletion of the Calmodulin-Binding Domain from Rice Glutamate Decarboxylase 3 (OsGAD3) Increases γ-Aminobutyric Acid Content in Grains,” *Rice*, vol. 13, no. 1, 2020, doi: 10.1186/s12284-020-00380-w.

[256] O. X. Dong *et al.*, “Marker-free carotenoid-enriched rice generated through targeted gene insertion using CRISPR-Cas9,” *Nat. Commun.*, vol. 11, no. 1, 2020, doi: 10.1038/s41467-020-14981-y.

[257] D. Zeng *et al.*, “Engineered Cas9 variant tools expand targeting scope of genome and base editing in rice,” *Plant Biotechnol. J.*, vol. 18, no. 6, 2020, doi: 10.1111/pbi.13293.

[258] K. Hua, X. Tao, W. Liang, Z. Zhang, R. Gou, and J. K. Zhu, “Simplified adenine base editors improve adenine base editing efficiency in rice,” *Plant Biotechnol. J.*, vol. 18, no. 3, 2020, doi: 10.1111/pbi.13244.

[259] F. Wang *et al.*, “Developing high-efficiency base editors by combining optimized synergistic core

components with new types of nuclear localization signal peptide," *Crop J.*, vol. 8, no. 3, pp. 408–417, 2020, doi: https://doi.org/10.1016/j.cj.2020.01.003.

[260] R. Xu, J. Li, X. Liu, T. Shan, R. Qin, and P. Wei, "Development of Plant Prime-Editing Systems for Precise Genome Editing," *Plant Commun.*, vol. 1, no. 3, p. 100043, May 2020, doi: 10.1016/J.XPLC.2020.100043.

[261] H. Butt, G. S. Rao, K. Sedeek, R. Aman, R. Kamel, and M. Mahfouz, "Engineering herbicide resistance via prime editing in rice," *Plant Biotechnol. J.*, vol. 18, no. 12, pp. 2370–2372, Dec. 2020, doi: https://doi.org/10.1111/pbi.13399.

[262] Q. Lin *et al.*, "Prime genome editing in rice and wheat," *Nat. Biotechnol.*, vol. 38, no. 5, pp. 582–585, 2020, doi: 10.1038/s41587-020-0455-x.

[263] H. Li, J. Li, J. Chen, L. Yan, and L. Xia, "Precise Modifications of Both Exogenous and Endogenous Genes in Rice by Prime Editing," *Mol. Plant*, vol. 13, no. 5, pp. 671–674, May 2020, doi: 10.1016/J.MOLP.2020.03.011.

[264] R. Qin, J. Li, X. Liu, R. Xu, J. Yang, and P. Wei, "SpCas9-NG self-targets the sgRNA sequence in plant genome editing," *Nat. Plants*, vol. 6, no. 3, pp. 197–201, 2020, doi: 10.1038/s41477-020-0603-9.

[265] M. Ming *et al.*, "CRISPR–Cas12b enables efficient plant genome engineering," *Nat. Plants*, vol. 6, no. 3, pp. 202–208, 2020, doi: 10.1038/s41477-020-0614-6.

[266] Y. Kuang *et al.*, "Base-Editing-Mediated Artificial Evolution of OsALS1 In Planta to Develop Novel Herbicide-Tolerant Rice Germplasms," *Mol. Plant*, vol. 13, no. 4, pp. 565–572, Apr. 2020, doi: 10.1016/J.MOLP.2020.01.010.

[267] F. Zhang *et al.*, "The SUN Domain Proteins OsSUN1 and OsSUN2 Play Critical but Partially Redundant Roles in Meiosis ," *Plant Physiol.*, vol. 183, no. 4, pp. 1517–1530, Aug. 2020, doi: 10.1104/pp.20.00140.

[268] Z. Ali *et al.*, "Fusion of the Cas9 endonuclease and the VirD2 relaxase facilitates homology-directed repair for precise genome engineering in rice," *Commun. Biol.*, vol. 3, no. 1, p. 44, 2020, doi: 10.1038/s42003-020-0768-9.

[269] C. Zhang *et al.*, "Highly efficient CRISPR-SaKKH tools for plant multiplex cytosine base editing," *Crop J.*, vol. 8, no. 3, pp. 418–423, 2020, doi: https://doi.org/10.1016/j.cj.2020.03.002.

[270] C. Yuyu *et al.*, "Effects of GS3 and GL3.1 for Grain Size Editing by CRISPR/Cas9 in Rice," *Rice Sci.*, vol. 27, no. 5, pp. 405–413, 2020, doi: https://doi.org/10.1016/j.rsci.2019.12.010.

[271] C. Zhang *et al.*, "Genome Engineering in Plant Using an Efficient CRISPR-xCas9 Toolset With an Expanded PAM Compatibility ," *Frontiers in Genome Editing* , vol. 2. 2020, [Online]. Available: https://www.frontiersin.org/articles/10.3389/fgeed.2020.618385.

[272] Q. Zhang, K. Yin, G. Liu, S. Li, M. Li, and J.-L. Qiu, "Fusing T5 exonuclease with Cas9 and Cas12a increases the frequency and size of deletion at target sites," *Sci. China Life Sci.*, vol. 63, no. 12, pp. 1918–1927, 2020, doi: 10.1007/s11427-020-1671-6.

[273] G. Nawaz *et al.*, "Knockout of Pi21 by CRISPR/Cas9 and iTRAQ-Based Proteomic Analysis of Mutants Revealed New Insights into M. oryzae Resistance in Elite Rice Line," *Genes*, vol. 11, no. 7. 2020, doi: 10.3390/genes11070735.

[274] C. Suketomo, T. Kazama, and K. Toriyama, “Fertility restoration of Chinese wild rice-type cytoplasmic male sterility by CRISPR/Cas9-mediated genome editing of nuclear-encoded *RETROGRADE-REGULATED MALE STERILITY*,” *Plant Biotechnol.*, vol. 37, no. 3, pp. 285–292, 2020, doi: 10.5511/plantbiotechnology.20.0326b.

[275] L. Huang *et al.*, “Creating novel Wx alleles with fine-tuned amylose levels and improved grain quality in rice by promoter editing using CRISPR/Cas9 system,” *Plant Biotechnol. J.*, vol. 18, no. 11, pp. 2164–2166, Nov. 2020, doi: https://doi.org/10.1111/pbi.13391.

[276] Y. He *et al.*, “Repurposing of Anthocyanin Biosynthesis for Plant Transformation and Genome Editing ,” *Frontiers in Genome Editing* , vol. 2. 2020, [Online]. Available: https://www.frontiersin.org/articles/10.3389/fgeed.2020.607982.

[277] E. Yue, H. Cao, and B. Liu, “OsmiR535, a Potential Genetic Editing Target for Drought and Salinity Stress Tolerance in Oryza sativa,” *Plants*, vol. 9, no. 10. 2020, doi: 10.3390/plants9101337.

[278] B. Wang *et al.*, “A novel CCCH-type zinc finger protein SAW1 activates OsGA20ox3 to regulate gibberellin homeostasis and anther development in rice,” *J. Integr. Plant Biol.*, vol. 62, no. 10, pp. 1594–1606, Oct. 2020, doi: https://doi.org/10.1111/jipb.12924.

[279] S. Nithya, Y. Kumam, S. Varanavasiappan, K. K. Kumar, and M. M. and D. Sudhakar, “Targeted mutation in eIF4G gene in rice,” *Electron. J. Plant Breed.*, vol. 11, no. 04 SE-Research Article, Dec. 2020, [Online]. Available: https://www.ejplantbreeding.org/index.php/EJPB/article/view/3699.

[280] K. A. Molla, J. Shih, and Y. Yang, “Single-nucleotide editing for zebra3 and wsl5 phenotypes in rice using CRISPR/Cas9-mediated adenine base editors,” *aBIOTECH*, vol. 1, no. 2, pp. 106–118, 2020, doi: 10.1007/s42994-020-00018-x.

[281] Y. Huang, Z. Han, N. Cheng, M. Luo, X. Bai, and Y. Xing, “Minor Effects of 11 Dof Family Genes Contribute to the Missing Heritability of Heading Date in Rice (Oryza sativa L.) ,” *Frontiers in Plant Science* , vol. 10. 2020, [Online]. Available: https://www.frontiersin.org/articles/10.3389/fpls.2019.01739.

[282] K. Negishi, M. Mikami, S. Toki, and M. Endo, “Enhanced FnCas12a-Mediated Targeted Mutagenesis Using crRNA With Altered Target Length in Rice ,” *Frontiers in Genome Editing* , vol. 2. 2020, [Online]. Available: https://www.frontiersin.org/articles/10.3389/fgeed.2020.608563.

[283] Y. Cui *et al.*, “CYCLIC NUCLEOTIDE-GATED ION CHANNELs 14 and 16 Promote Tolerance to Heat and Chilling in Rice ,” *Plant Physiol.*, vol. 183, no. 4, pp. 1794–1808, Aug. 2020, doi: 10.1104/pp.20.00591.

[284] Q. Gong *et al.*, “Nicking Endonuclease-Mediated Vector Construction Strategies for Plant Gene Functional Research,” *Plants*, vol. 9, no. 9. 2020, doi: 10.3390/plants9091090.

[285] A. Nishizawa-Yokoi, M. Mikami, and S. Toki, “A Universal System of CRISPR/Cas9-Mediated Gene Targeting Using All-in-One Vector in Plants ,” *Frontiers in Genome Editing* , vol. 2. 2020, [Online]. Available: https://www.frontiersin.org/articles/10.3389/fgeed.2020.604289.

[286] J. Ren *et al.*, “Expanding the scope of genome editing with SpG and SpRY variants in rice,” *Sci. China Life Sci.*, vol. 64, no. 10, pp. 1784–1787, 2021, doi: 10.1007/s11427-020-1883-5.

[287] M. Jiang *et al.*, “An Inositol 1,3,4,5,6-Pentakisphosphate 2-Kinase 1 Mutant with a 33-nt Deletion Showed Enhanced Tolerance to Salt and Drought Stress in Rice,” *Plants*, vol. 10, no. 1. 2021, doi:

10.3390/plants10010023.

[288] D. Liu *et al.*, "Diversification of plant agronomic traits by genome editing of brassinosteroid signaling family genes in rice," *Plant Physiol.*, 2021, doi: 10.1093/plphys/kiab394.

[289] S. Sretenovic, D. Yin, A. Levav, J. D. Selengut, S. M. Mount, and Y. Qi, "Expanding plant genome-editing scope by an engineered iSpyMacCas9 system that targets A-rich PAM sequences," *Plant Commun.*, vol. 2, no. 2, p. 100101, Mar. 2021, doi: 10.1016/J.XPLC.2020.100101.

[290] X. Liu *et al.*, "Fine-tuning Flowering Time via Genome Editing of Upstream Open Reading Frames of Heading Date 2 in Rice," *Rice*, vol. 14, no. 1, 2021, doi: 10.1186/s12284-021-00504-w.

[291] W. Wang, S. Ma, P. Hu, Y. Ji, and F. Sun, "Genome Editing of Rice eIF4G Loci Confers Partial Resistance to Rice Black-Streaked Dwarf Virus," *Viruses*, vol. 13, no. 10, 2021, doi: 10.3390/v13102100.

[292] M. Molina-Risco, O. Ibarra, M. Faion-Molina, B. Kim, E. M. Septiningsih, and M. J. Thomson, "Optimizing agrobacterium-mediated transformation and crispr-cas9 gene editing in the tropical japonica rice variety presidio," *Int. J. Mol. Sci.*, vol. 22, no. 20, 2021, doi: 10.3390/ijms222010909.

[293] B. Usman *et al.*, "CRISPR/Cas9 guided mutagenesis of grain size 3 confers increased rice (Oryza sativa L.) grain length by regulating cysteine proteinase inhibitor and ubiquitin-related proteins," *Int. J. Mol. Sci.*, vol. 22, no. 6, 2021, doi: 10.3390/ijms22063225.

[294] H. Pak *et al.*, "Creation of male-sterile lines that can be restored to fertility by exogenous methyl jasmonate for the establishment of a two-line system for the hybrid production of rice (Oryza sativa L.)," *Plant Biotechnol. J.*, vol. 19, no. 2, pp. 365–374, Feb. 2021, doi: https://doi.org/10.1111/pbi.13471.

[295] M. Carlessi, L. Mariotti, F. Giaume, F. Fornara, P. Perata, and S. Gonzali, "Targeted knockout of the gene OsHOL1 removes methyl iodide emissions from rice plants," *Sci. Rep.*, vol. 11, no. 1, p. 17010, 2021, doi: 10.1038/s41598-021-95198-x.

[296] F. Guo *et al.*, "Functional analysis of auxin receptor OsTIR1/OsAFB family members in rice grain yield, tillering, plant height, root system, germination, and auxinic herbicide resistance," *New Phytol.*, vol. 229, no. 5, pp. 2676–2692, Mar. 2021, doi: https://doi.org/10.1111/nph.17061.

[297] C. Liu *et al.*, "Concurrent Disruption of Genetic Interference and Increase of Genetic Recombination Frequency in Hybrid Rice Using CRISPR/Cas9 ," *Frontiers in Plant Science* , vol. 12. 2021, [Online]. Available: https://www.frontiersin.org/articles/10.3389/fpls.2021.757152.

[298] G. Ma *et al.*, "CRISPR/Sc++-mediated genome editing in rice," *J. Integr. Plant Biol.*, vol. 63, no. 9, pp. 1606–1610, Sep. 2021, doi: https://doi.org/10.1111/jipb.13166.

[299] Z. Xu *et al.*, "SpRY greatly expands the genome editing scope in rice with highly flexible PAM recognition," *Genome Biol.*, vol. 22, no. 1, p. 6, 2021, doi: 10.1186/s13059-020-02231-9.

[300] J. Li, R. Xu, R. Qin, X. Liu, F. Kong, and P. Wei, "Genome editing mediated by SpCas9 variants with broad non-canonical PAM compatibility in plants," *Mol. Plant*, vol. 14, no. 2, pp. 352–360, Feb. 2021, doi: 10.1016/J.MOLP.2020.12.017.

[301] Y. Zhang *et al.*, "Expanding the scope of plant genome engineering with Cas12a orthologs and highly multiplexable editing systems," *Nat. Commun.*, vol. 12, no. 1, 2021, doi: 10.1038/s41467-021-22330-w.

[302] R. Zhang *et al.*, "Generating broad-spectrum tolerance to ALS-inhibiting herbicides in rice by base editing," *Sci. China Life Sci.*, vol. 64, no. 10, 2021, doi: 10.1007/s11427-020-1800-5.

[303] Y. Zhou *et al.*, "Engineering of rice varieties with enhanced resistances to both blast and bacterial blight diseases via CRISPR/Cas9," *Plant Biotechnol. J.*, vol. n/a, no. n/a, Dec. 2021, doi: https://doi.org/10.1111/pbi.13766.

[304] H. Yu *et al.*, "A route to de novo domestication of wild allotetraploid rice," *Cell*, vol. 184, no. 5, pp. 1156-1170.e14, 2021, doi: https://doi.org/10.1016/j.cell.2021.01.013.

[305] K. Yu *et al.*, "Highly efficient generation of bacterial leaf blight-resistant and transgene-free rice using a genome editing and multiplexed selection system," *BMC Plant Biol.*, vol. 21, no. 1, 2021, doi: 10.1186/s12870-021-02979-7.

[306] H. Yu *et al.*, "A route to de novo domestication of wild allotetraploid rice," *Cell*, vol. 184, no. 5, pp. 1156-1170.e14, Mar. 2021, doi: 10.1016/J.CELL.2021.01.013.

[307] Q. Ren *et al.*, "PAM-less plant genome editing using a CRISPR–SpRY toolbox," *Nat. Plants*, vol. 7, no. 1, pp. 25–33, 2021, doi: 10.1038/s41477-020-00827-4.

[308] B. Li *et al.*, "Efficient Breeding of Early-Maturing Rice Cultivar by Editing PHYC via CRISPR/Cas9," *Rice*, vol. 14, no. 1, p. 86, 2021, doi: 10.1186/s12284-021-00527-3.

[309] Nurhayati, S. W. Ardie, T. J. Santoso, and Sudarsono, "Crispr/cas9-mediated genome editing in rice cv. Ipb3s results in a semi-dwarf phenotypic mutant," *Biodiversitas*, vol. 22, no. 9, 2021, doi: 10.13057/biodiv/d220924.

[310] Q. Ren *et al.*, "Improved plant cytosine base editors with high editing activity, purity, and specificity," *Plant Biotechnol. J.*, vol. 19, no. 10, 2021, doi: 10.1111/pbi.13635.

[311] T. Liu *et al.*, "The ScCas9++ variant expands the CRISPR toolbox for genome editing in plants," *J. Integr. Plant Biol.*, vol. 63, no. 9, 2021, doi: 10.1111/jipb.13164.

[312] Z. Ni *et al.*, "Engineering Resistance to Bacterial Blight and Bacterial Leaf Streak in Rice," *Rice*, vol. 14, no. 1, 2021, doi: 10.1186/s12284-021-00482-z.

[313] R. Xu *et al.*, "Genome editing with type II-C CRISPR-Cas9 systems from Neisseria meningitidis in rice," *Plant Biotechnol. J.*, vol. n/a, no. n/a, Sep. 2021, doi: https://doi.org/10.1111/pbi.13716.

[314] B. Bahariah, M. Y. A. Masani, O. A. Rasid, and G. K. A. Parveez, "Multiplex CRISPR/Cas9-mediated genome editing of the FAD2 gene in rice: a model genome editing system for oil palm," *J. Genet. Eng. Biotechnol.*, vol. 19, no. 1, p. 86, 2021, doi: 10.1186/s43141-021-00185-4.

[315] X. Yang *et al.*, "OsTTG1, a WD40 repeat gene, regulates anthocyanin biosynthesis in rice," *Plant J.*, vol. 107, no. 1, pp. 198–214, Jul. 2021, doi: https://doi.org/10.1111/tpj.15285.

[316] D. Luo, J. C. Huguet-Tapia, R. T. Raborn, F. F. White, V. P. Brendel, and B. Yang, "The Xa7 resistance gene guards the rice susceptibility gene SWEET14 against exploitation by the bacterial blight pathogen," *Plant Commun.*, vol. 2, no. 3, p. 100164, 2021, doi: https://doi.org/10.1016/j.xplc.2021.100164.

[317] D. Tezuka, H. Matsuura, W. Saburi, H. Mori, and R. Imai, "A Ubiquitously Expressed UDP-Glucosyltransferase, UGT74J1, Controls Basal Salicylic Acid Levels in Rice," *Plants*, vol. 10, no. 9. 2021,

doi: 10.3390/plants10091875.

[318] K. Teng, X. Wang, X. Guo, Y. Liu, and R. Li, “Generation of a New Glutinous Photothermosensitive Genic-Male-Sterile (PTGMS) Line by CRISPR/Cas9-Directed Mutagenesis of Wx in Rice (Oryza sativa L.),” *Agriculture*, vol. 11, no. 11. 2021, doi: 10.3390/agriculture11111044.

[319] D. Yan *et al.*, “High-efficiency and multiplex adenine base editing in plants using new TadA variants,” *Mol. Plant*, vol. 14, no. 5, pp. 722–731, 2021, doi: 10.1016/j.molp.2021.02.007.

[320] N. Yang *et al.*, “Editing gene families by CRISPR/Cas9: accelerating the isolation of multiple transgene-free null mutant combinations with much reduced labor-intensive analysis,” *Plant Biotechnol. J.*, vol. n/a, no. n/a, Nov. 2021, doi: https://doi.org/10.1111/pbi.13744.

[321] H. Zhang *et al.*, “An Editing-Site-Specific PCR Method for Detection and Quantification of CAO1-Edited Rice,” *Foods*, vol. 10, no. 6. 2021, doi: 10.3390/foods10061209.

[322] A. M. Luong *et al.*, “Functional Diversification of euANT/PLT Genes in Oryza sativa Panicle Architecture Determination ,” *Frontiers in Plant Science* , vol. 12. 2021, [Online]. Available: https://www.frontiersin.org/articles/10.3389/fpls.2021.692955.

[323] X. Wu *et al.*, “Enhancing rice grain production by manipulating the naturally evolved cis-regulatory element-containing inverted repeat sequence of OsREM20,” *Mol. Plant*, vol. 14, no. 6, pp. 997–1011, 2021, doi: https://doi.org/10.1016/j.molp.2021.03.016.

[324] R. Xu, F. Kong, R. Qin, J. Li, X. Liu, and P. Wei, “Development of an efficient plant dual cytosine and adenine editor,” *J. Integr. Plant Biol.*, vol. 63, no. 9, pp. 1600–1605, Sep. 2021, doi: https://doi.org/10.1111/jipb.13146.

[325] A. N. Chan, L.-L. Wang, Y.-J. Zhu, Y.-Y. Fan, J.-Y. Zhuang, and Z.-H. Zhang, “Identification through fine mapping and verification using CRISPR/Cas9-targeted mutagenesis for a minor QTL controlling grain weight in rice,” *Theor. Appl. Genet.*, vol. 134, no. 1, pp. 327–337, 2021, doi: 10.1007/s00122-020-03699-6.

[326] Y. Lu *et al.*, “A donor-DNA-free CRISPR/Cas-based approach to gene knock-up in rice,” *Nat. Plants*, vol. 7, no. 11, pp. 1445–1452, 2021, doi: 10.1038/s41477-021-01019-4.

[327] T. Arulganesh *et al.*, “Genome editing of elite rice cultivar CO51 for bacterial leaf blight resistance,” *Electron. J. Plant Breed.*, vol. 12, no. 4 SE-Research Article, Dec. 2021, [Online]. Available: https://www.ejplantbreeding.org/index.php/EJPB/article/view/4051.

[328] J. Zhou *et al.*, “Efficient deletion of multiple circle RNA loci by CRISPR-Cas9 reveals Os06circ02797 as a putative sponge for OsMIR408 in rice,” *Plant Biotechnol. J.*, vol. 19, no. 6, pp. 1240–1252, Jun. 2021, doi: https://doi.org/10.1111/pbi.13544.

[329] V. M. M. Achary and M. K. Reddy, “CRISPR-Cas9 mediated mutation in GRAIN WIDTH and WEIGHT2 (GW2) locus improves aleurone layer and grain nutritional quality in rice,” *Sci. Rep.*, vol. 11, no. 1, p. 21941, 2021, doi: 10.1038/s41598-021-00828-z.

[330] D. Hu *et al.*, “Multiplex CRISPR-Cas9 editing of DNA methyltransferases in rice uncovers a class of non-CG methylation specific for GC-rich regions,” *Plant Cell*, vol. 33, no. 9, pp. 2950–2964, Sep. 2021, doi: 10.1093/plcell/koab162.

[331] M. T. Andrew-Peter-Leon, R. Selvaraj, K. K. Kumar, M. Muthamilarasan, J. K. Yasin, and M. A. Pillai, "Loss of Function of OsFBX267 and OsGA20ox2 in Rice Promotes Early Maturing and Semi-Dwarfism in γ-Irradiated IWP and Genome-Edited Pusa Basmati-1 ," *Frontiers in Plant Science* , vol. 12. 2021, [Online]. Available: https://www.frontiersin.org/articles/10.3389/fpls.2021.714066.

[332] Y. Liang, S. Biswas, B. Kim, J. Bailey-Serres, and E. M. Septiningsih, "Improved Transformation and Regeneration of Indica Rice: Disruption of SUB1A as a Test Case via CRISPR-Cas9," *International Journal of Molecular Sciences* , vol. 22, no. 13. 2021, doi: 10.3390/ijms22136989.

[333] Y. Yu *et al.*, "Overexpression of the rice ORANGE gene OsOR negatively regulates carotenoid accumulation, leads to higher tiller numbers and decreases stress tolerance in Nipponbare rice," *Plant Sci.*, vol. 310, p. 110962, 2021, doi: https://doi.org/10.1016/j.plantsci.2021.110962.

[334] J. E. Reeger, M. Wheatley, Y. Yang, and K. M. Brown, "Targeted mutation of transcription factor genes alters metaxylem vessel size and number in rice roots," *Plant Direct*, vol. 5, no. 6, p. e00328, Jun. 2021, doi: https://doi.org/10.1002/pld3.328.

[335] S. Omukai, S. Arimura, K. Toriyama, and T. Kazama, "Disruption of mitochondrial open reading frame 352 partially restores pollen development in cytoplasmic male sterile rice," *Plant Physiol.*, vol. 187, no. 1, pp. 236–246, Sep. 2021, doi: 10.1093/plphys/kiab236.

[336] S. Sretenovic *et al.*, "Exploring C-To-G Base Editing in Rice, Tomato, and Poplar ," *Frontiers in Genome Editing* , vol. 3. 2021, [Online]. Available: https://www.frontiersin.org/articles/10.3389/fgeed.2021.756766.

[337] J. Li *et al.*, "Rice SPL10 positively regulates trichome development through expression of HL6 and auxin-related genes," *J. Integr. Plant Biol.*, vol. 63, no. 8, pp. 1521–1537, Aug. 2021, doi: https://doi.org/10.1111/jipb.13140.

[338] Y. Gao *et al.*, "Sheath blight resistance in rice is negatively regulated by WRKY53 via SWEET2a activation," *Biochem. Biophys. Res. Commun.*, vol. 585, pp. 117–123, 2021, doi: https://doi.org/10.1016/j.bbrc.2021.11.042.

[339] G. Young Chae, W.-J. Hong, M. Jeong Jang, K.-H. Jung, and S. Kim, "Recurrent mutations promote widespread structural and functional divergence of MULE-derived genes in plants," *Nucleic Acids Res.*, vol. 49, no. 20, pp. 11765–11777, Nov. 2021, doi: 10.1093/nar/gkab932.

[340] Y. Kumam *et al.*, "Genome editing of indica rice ASD16 for imparting resistance against rice tungro disease," *J. Plant Biochem. Biotechnol.*, vol. 31, no. 4, pp. 880–893, 2022, doi: 10.1007/s13562-021-00765-y.

[341] Z. Xiang *et al.*, "Agrobacterium-Mediated High-Efficiency Genetic Transformation and Genome Editing of Chaling Common Wild Rice (Oryza rufipogon Griff.) Using Scutellum Tissue of Embryos in Mature Seeds ," *Frontiers in Plant Science* , vol. 13. 2022, [Online]. Available: https://www.frontiersin.org/articles/10.3389/fpls.2022.849666.

[342] A. K. Biswal *et al.*, "Novel Mutant Alleles Reveal a Role of the Extra-Large G Protein in Rice Grain Filling, Panicle Architecture, Plant Growth, and Disease Resistance ," *Frontiers in Plant Science* , vol. 12. 2022, [Online]. Available: https://www.frontiersin.org/articles/10.3389/fpls.2021.782960.

[343] T. Thakur *et al.*, "Efficient Genetic Transformation of Rice for CRISPR/Cas9 Mediated Genome-Editing

and Stable Overexpression Studies: A Case Study on Rice Lipase 1 and Galactinol Synthase Encoding Genes," *Agronomy*, vol. 12, no. 1. 2022, doi: 10.3390/agronomy12010179.

[344] R. Li, J. Zhang, Z. Li, R. J. Peters, and B. Yang, "Dissecting the labdane-related diterpenoid biosynthetic gene clusters in rice reveals directional cross-cluster phytotoxicity," *New Phytol.*, vol. 233, no. 2, pp. 878–889, Jan. 2022, doi: https://doi.org/10.1111/nph.17806.

[345] C.-Y. Kim *et al.*, "A rice gene encoding glycosyl hydrolase plays contrasting roles in immunity depending on the type of pathogens," *Mol. Plant Pathol.*, vol. 23, no. 3, pp. 400–416, Mar. 2022, doi: https://doi.org/10.1111/mpp.13167.

[346] K. Kawai *et al.*, "Evolutionary alterations in gene expression and enzymatic activities of gibberellin 3-oxidase 1 in Oryza," *Commun. Biol.*, vol. 5, no. 1, p. 67, 2022, doi: 10.1038/s42003-022-03008-5.

[347] K. Chen *et al.*, "A FLASH pipeline for arrayed CRISPR library construction and the gene function discovery of rice receptor-like kinases," *Mol. Plant*, vol. 15, no. 2, pp. 243–257, 2022, doi: https://doi.org/10.1016/j.molp.2021.09.015.

[348] J. Tan *et al.*, "An Efficient Marker Gene Excision Strategy Based on CRISPR/Cas9-Mediated Homology-Directed Repair in Rice," *International Journal of Molecular Sciences*, vol. 23, no. 3. 2022, doi: 10.3390/ijms23031588.

[349] L. Luo *et al.*, "TRANS-ACTING SIRNA3-derived short interfering RNAs confer cleavage of mRNAs in rice," *Plant Physiol.*, vol. 188, no. 1, pp. 347–362, Jan. 2022, doi: 10.1093/plphys/kiab452.

[350] Z. Wang *et al.*, "An aspartic protease 47 causes quantitative recessive resistance to rice black-streaked dwarf virus disease and southern rice black-streaked dwarf virus disease," *New Phytol.*, vol. 233, no. 6, pp. 2520–2533, Mar. 2022, doi: https://doi.org/10.1111/nph.17961.

[351] Y. Zong *et al.*, "An engineered prime editor with enhanced editing efficiency in plants," *Nat. Biotechnol.*, vol. 40, no. 9, pp. 1394–1402, 2022, doi: 10.1038/s41587-022-01254-w.

[352] Y.-N. Lin *et al.*, "Rice Cell Division Cycle 20s are required for faithful chromosome segregation and cytokinesis during meiosis," *Plant Physiol.*, vol. 188, no. 2, pp. 1111–1128, Feb. 2022, doi: 10.1093/plphys/kiab543.

[353] W. Jiang, H. Zhou, H. Bi, M. Fromm, B. Yang, and D. P. Weeks, "Demonstration of CRISPR/Cas9/sgRNA-mediated targeted gene modification in Arabidopsis, tobacco, sorghum and rice," *Nucleic Acids Res.*, vol. 41, no. 20, pp. e188–e188, Nov. 2013, doi: 10.1093/nar/gkt780.

[354] P. Che *et al.*, "Developing a flexible, high-efficiency Agrobacterium-mediated sorghum transformation system with broad application," *Plant Biotechnol. J.*, vol. 16, no. 7, p. 1388, Jul. 2018, doi: 10.1111/PBI.12879.

[355] A. Li *et al.*, "Editing of an Alpha-Kafirin Gene Family Increases, Digestibility and Protein Quality in Sorghum," *Plant Physiol.*, vol. 177, no. 4, p. 1425, Aug. 2018, doi: 10.1104/PP.18.00200.

[356] S. N. Char *et al.*, "An Agrobacterium-delivered CRISPR/Cas9 system for targeted mutagenesis in sorghum," *Plant Biotechnol. J.*, vol. 18, no. 2, p. 319, Feb. 2020, doi: 10.1111/PBI.13229.

[357] E. J. Brant, M. C. Baloglu, A. Parikh, and F. Altpeter, "CRISPR/Cas9 mediated targeted mutagenesis of

LIGULELESS-1 in sorghum provides a rapidly scorable phenotype by altering leaf inclination angle," *Biotechnol. J.*, p. 2100237, 2021, doi: 10.1002/BIOT.202100237.

[358] D. Zhang *et al.*, "Creation of fragrant sorghum by CRISPR/Cas9," *J. Integr. Plant Biol.*, vol. 64, no. 5, 2022, doi: 10.1111/jipb.13232.

[359] K. Aregawi *et al.*, "Morphogene-assisted transformation of Sorghum bicolor allows more efficient genome editing," *Plant Biotechnol. J.*, vol. 20, no. 4, 2022, doi: 10.1111/pbi.13754.

[360] S. J. Curtin *et al.*, "Targeted Mutagenesis of Duplicated Genes in Soybean with Zinc-Finger Nucleases ," *Plant Physiol.*, vol. 156, no. 2, pp. 466–473, Jun. 2011, doi: 10.1104/pp.111.172981.

[361] W. Haun *et al.*, "Improved soybean oil quality by targeted mutagenesis of the fatty acid desaturase 2 gene family," *Plant Biotechnol. J.*, vol. 12, no. 7, pp. 934–940, Sep. 2014, doi: https://doi.org/10.1111/pbi.12201.

[362] T. B. Jacobs, P. R. LaFayette, R. J. Schmitz, and W. A. Parrott, "Targeted genome modifications in soybean with CRISPR/Cas9," *BMC Biotechnol.*, vol. 15, no. 1, p. 16, 2015, doi: 10.1186/s12896-015-0131-2.

[363] J.-M. Michno, X. Wang, J. Liu, S. J. Curtin, T. J. Y. Kono, and R. M. Stupar, "CRISPR/Cas mutagenesis of soybean and Medicago truncatula using a new web-tool and a modified Cas9 enzyme," *GM Crops Food*, vol. 6, no. 4, pp. 243–252, Oct. 2015, doi: 10.1080/21645698.2015.1106063.

[364] X. Sun *et al.*, "Targeted mutagenesis in soybean using the CRISPR-Cas9 system," *Sci. Rep.*, vol. 5, no. 1, p. 10342, 2015, doi: 10.1038/srep10342.

[365] Z. Li *et al.*, "Cas9-Guide RNA Directed Genome Editing in Soybean," *Plant Physiol.*, vol. 169, no. 2, pp. 960–970, Oct. 2015, doi: 10.1104/pp.15.00783.

[366] Z. L. Demorest *et al.*, "Direct stacking of sequence-specific nuclease-induced mutations to produce high oleic and low linolenic soybean oil," *BMC Plant Biol.*, vol. 16, no. 1, p. 225, 2016, doi: 10.1186/s12870-016-0906-1.

[367] F. Tang, S. Yang, J. Liu, and H. Zhu, "Rj4, a Gene Controlling Nodulation Specificity in Soybeans, Encodes a Thaumatin-Like Protein But Not the One Previously Reported," *Plant Physiol.*, vol. 170, no. 1, pp. 26–32, Jan. 2016, doi: 10.1104/pp.15.01661.

[368] S. J. Curtin *et al.*, "MicroRNA Maturation and MicroRNA Target Gene Expression Regulation Are Severely Disrupted in Soybean dicer-like1 Double Mutants," *G3 Genes|Genomes|Genetics*, vol. 6, no. 2, pp. 423–433, Feb. 2016, doi: 10.1534/g3.115.022137.

[369] H. Du *et al.*, "Efficient targeted mutagenesis in soybean by TALENs and CRISPR/Cas9," *J. Biotechnol.*, vol. 217, pp. 90–97, 2016, doi: https://doi.org/10.1016/j.jbiotec.2015.11.005.

[370] Y. Fan, J. Liu, S. Lyu, Q. Wang, S. Yang, and H. Zhu, "The Soybean Rfg1 Gene Restricts Nodulation by Sinorhizobium fredii USDA193 ," *Frontiers in Plant Science* , vol. 8. 2017, [Online]. Available: https://www.frontiersin.org/articles/10.3389/fpls.2017.01548.

[371] S. J. Curtin *et al.*, "CRISPR/Cas9 and TALENs generate heritable mutations for genes involved in small RNA processing of Glycine max and Medicago truncatula," *Plant Biotechnol. J.*, vol. 16, no. 6, pp. 1125–

1137, Jun. 2018, doi: https://doi.org/10.1111/pbi.12857.

[372] Y. Kanazashi *et al.*, “Simultaneous site-directed mutagenesis of duplicated loci in soybean using a single guide RNA,” *Plant Cell Rep.*, vol. 37, no. 3, pp. 553–563, 2018, doi: 10.1007/s00299-018-2251-3.

[373] Y. Cai *et al.*, “CRISPR/Cas9-Mediated Deletion of Large Genomic Fragments in Soybean,” *International Journal of Molecular Sciences*, vol. 19, no. 12. 2018, doi: 10.3390/ijms19123835.

[374] N. al Amin *et al.*, “CRISPR-Cas9 mediated targeted disruption of FAD2–2 microsomal omega-6 desaturase in soybean (Glycine max.L),” *BMC Biotechnol.*, vol. 19, no. 1, p. 9, 2019, doi: 10.1186/s12896-019-0501-2.

[375] Y. Huang *et al.*, “GmHsp90A2 is involved in soybean heat stress as a positive regulator,” *Plant Sci.*, vol. 285, pp. 26–33, 2019, doi: https://doi.org/10.1016/j.plantsci.2019.04.016.

[376] A. Bao *et al.*, “CRISPR/Cas9-mediated targeted mutagenesis of GmSPL9 genes alters plant architecture in soybean,” *BMC Plant Biol.*, vol. 19, no. 1, p. 131, 2019, doi: 10.1186/s12870-019-1746-6.

[377] Q. Cheng *et al.*, “CRISPR/Cas9-mediated targeted mutagenesis of GmLHY genes alters plant height and internode length in soybean,” *BMC Plant Biol.*, vol. 19, no. 1, p. 562, 2019, doi: 10.1186/s12870-019-2145-8.

[378] P. T. Do *et al.*, “Demonstration of highly efficient dual gRNA CRISPR/Cas9 editing of the homeologous GmFAD2–1A and GmFAD2–1B genes to yield a high oleic, low linoleic and α-linolenic acid phenotype in soybean,” *BMC Plant Biol.*, vol. 19, no. 1, p. 311, 2019, doi: 10.1186/s12870-019-1906-8.

[379] J. Han, B. Guo, Y. Guo, B. Zhang, X. Wang, and L.-J. Qiu, “Creation of Early Flowering Germplasm of Soybean by CRISPR/Cas9 Technology ,” *Frontiers in Plant Science* , vol. 10. 2019, [Online]. Available: https://www.frontiersin.org/articles/10.3389/fpls.2019.01446.

[380] C. Li *et al.*, “Mutagenesis of seed storage protein genes in Soybean using CRISPR/Cas9,” *BMC Res. Notes*, vol. 12, no. 1, p. 176, 2019, doi: 10.1186/s13104-019-4207-2.

[381] N. D. Bonawitz *et al.*, “Zinc finger nuclease-mediated targeting of multiple transgenes to an endogenous soybean genomic locus via non-homologous end joining,” *Plant Biotechnol. J.*, vol. 17, no. 4, pp. 750–761, Apr. 2019, doi: https://doi.org/10.1111/pbi.13012.

[382] B. W. Campbell *et al.*, “Functional analysis and development of a CRISPR/Cas9 allelic series for a CPR5 ortholog necessary for proper growth of soybean trichomes,” *Sci. Rep.*, vol. 9, no. 1, p. 14757, 2019, doi: 10.1038/s41598-019-51240-7.

[383] S. Wang *et al.*, “The Soybean Sugar Transporter GmSWEET15 Mediates Sucrose Export from Endosperm to Early Embryo,” *Plant Physiol.*, vol. 180, no. 4, pp. 2133–2141, Aug. 2019, doi: 10.1104/pp.19.00641.

[384] Y. Wang *et al.*, “Light- and temperature-entrainable circadian clock in soybean development,” *Plant. Cell Environ.*, vol. 43, no. 3, pp. 637–648, Mar. 2020, doi: https://doi.org/10.1111/pce.13678.

[385] Y. Cai *et al.*, “Target base editing in soybean using a modified CRISPR/Cas9 system,” *Plant Biotechnol. J.*, vol. 18, no. 10, pp. 1996–1998, Oct. 2020, doi: https://doi.org/10.1111/pbi.13386.

[386] L. Wang *et al.*, “Natural variation and CRISPR/Cas9-mediated mutation in GmPRR37 affect photoperiodic flowering and contribute to regional adaptation of soybean,” *Plant Biotechnol. J.*, vol. 18, no. 9, pp.

1869–1881, Sep. 2020, doi: https://doi.org/10.1111/pbi.13346.

[387] J. Wang *et al.*, "Generation of seed lipoxygenase-free soybean using CRISPR-Cas9," *Crop J.*, vol. 8, no. 3, pp. 432–439, 2020, doi: https://doi.org/10.1016/j.cj.2019.08.008.

[388] N. Wu *et al.*, "Construction and Analysis of GmFAD2-1A and GmFAD2-2A Soybean Fatty Acid Desaturase Mutants Based on CRISPR/Cas9 Technology," *International Journal of Molecular Sciences*, vol. 21, no. 3. 2020, doi: 10.3390/ijms21031104.

[389] P. Zhang *et al.*, "Multiplex CRISPR/Cas9-mediated metabolic engineering increases soya bean isoflavone content and resistance to soya bean mosaic virus," *Plant Biotechnol. J.*, vol. 18, no. 6, pp. 1384–1395, Jun. 2020, doi: https://doi.org/10.1111/pbi.13302.

[390] C. Yang *et al.*, "GmNAC8 acts as a positive regulator in soybean drought stress," *Plant Sci.*, vol. 293, p. 110442, 2020, doi: https://doi.org/10.1016/j.plantsci.2020.110442.

[391] L. Chen *et al.*, "Soybean AP1 homologs control flowering time and plant height," *J. Integr. Plant Biol.*, vol. 62, no. 12, pp. 1868–1879, Dec. 2020, doi: https://doi.org/10.1111/jipb.12988.

[392] C. Li *et al.*, "A Domestication-Associated Gene GmPRR3b Regulates the Circadian Clock and Flowering Time in Soybean," *Mol. Plant*, vol. 13, no. 5, pp. 745–759, 2020, doi: https://doi.org/10.1016/j.molp.2020.01.014.

[393] W. Wang *et al.*, "GmNMHC5, A Neoteric Positive Transcription Factor of Flowering and Maturity in Soybean," *Plants*, vol. 9, no. 6. 2020, doi: 10.3390/plants9060792.

[394] J. Ma, S. Yang, D. Wang, K. Tang, X. X. Feng, and X. Z. Feng, "Genetic Mapping of a Light-Dependent Lesion Mimic Mutant Reveals the Function of Coproporphyrinogen III Oxidase Homolog in Soybean ," *Frontiers in Plant Science* , vol. 11. 2020, [Online]. Available: https://www.frontiersin.org/articles/10.3389/fpls.2020.00557.

[395] J. Jia *et al.*, "Soybean DICER-LIKE2 Regulates Seed Coat Color via Production of Primary 22-Nucleotide Small Interfering RNAs from Long Inverted Repeats," *Plant Cell*, vol. 32, no. 12, pp. 3662–3673, Dec. 2020, doi: 10.1105/tpc.20.00562.

[396] K. S. Virdi *et al.*, "Similar Seed Composition Phenotypes Are Observed From CRISPR-Generated In-Frame and Knockout Alleles of a Soybean KASI Ortholog ," *Frontiers in Plant Science* , vol. 11. 2020, [Online]. Available: https://www.frontiersin.org/articles/10.3389/fpls.2020.01005.

[397] H. Le *et al.*, "CRISPR/Cas9-Mediated Knockout of Galactinol Synthase-Encoding Genes Reduces Raffinose Family Oligosaccharide Levels in Soybean Seeds ," *Frontiers in Plant Science* , vol. 11. 2020, [Online]. Available: https://www.frontiersin.org/articles/10.3389/fpls.2020.612942.

[398] S. Sugano *et al.*, "Simultaneous induction of mutant alleles of two allergenic genes in soybean by using site-directed mutagenesis," *BMC Plant Biol.*, vol. 20, no. 1, p. 513, 2020, doi: 10.1186/s12870-020-02708-6.

[399] K. Adachi *et al.*, "Site-directed mutagenesis by biolistic transformation efficiently generates inheritable mutations in a targeted locus in soybean somatic embryos and transgene-free descendants in the T1 generation," *Transgenic Res.*, vol. 30, no. 1, pp. 77–89, 2021, doi: 10.1007/s11248-020-00229-4.

[400] X. Fang *et al.*, “MS1 is essential for male fertility by regulating the microsporocyte cell plate expansion in soybean,” *Sci. China Life Sci.*, vol. 64, no. 9, pp. 1533–1545, 2021, doi: 10.1007/s11427-021-1973-0.

[401] F. Niu, Q. Jiang, X. Sun, Z. Hu, L. Wang, and H. Zhang, “Large DNA fragment deletion in *lncRNA77580* regulates neighboring gene expression in soybean (*Glycine max*),” *Funct. Plant Biol.*, vol. 48, no. 11, pp. 1139–1147, 2021, [Online]. Available: https://doi.org/10.1071/FP20400.

[402] T. Wang *et al.*, “Confirmation of GmPPR576 as a fertility restorer gene of cytoplasmic male sterility in soybean,” *J. Exp. Bot.*, vol. 72, no. 22, pp. 7729–7742, Dec. 2021, doi: 10.1093/jxb/erab382.

[403] Y. Xiao, B. Karikari, L. Wang, F. Chang, and T. Zhao, “Structure characterization and potential role of soybean phospholipases A multigene family in response to multiple abiotic stress uncovered by CRISPR/Cas9 technology,” *Environ. Exp. Bot.*, vol. 188, p. 104521, 2021, doi: https://doi.org/10.1016/j.envexpbot.2021.104521.

[404] Z. Li *et al.*, “Multiplex CRISPR/Cas9-mediated knockout of soybean LNK2 advances flowering time,” *Crop J.*, vol. 9, no. 4, pp. 767–776, 2021, doi: https://doi.org/10.1016/j.cj.2020.09.005.

[405] J. Ma, S. Sun, J. Whelan, and H. Shou, “CRISPR/Cas9-Mediated Knockout of GmFATB1 Significantly Reduced the Amount of Saturated Fatty Acids in Soybean Seeds,” *International Journal of Molecular Sciences*, vol. 22, no. 8. 2021, doi: 10.3390/ijms22083877.

[406] B. Jiang *et al.*, “The cloning and CRISPR/Cas9-mediated mutagenesis of a male sterility gene MS1 of soybean,” *Plant Biotechnol. J.*, vol. 19, no. 6, pp. 1098–1100, Jun. 2021, doi: https://doi.org/10.1111/pbi.13601.

[407] Z. Cai *et al.*, “CRISPR/Cas9-mediated gene editing of GmJAGGED1 increased yield in the low-latitude soybean variety Huachun 6,” *Plant Biotechnol. J.*, vol. 19, no. 10, pp. 1898–1900, Oct. 2021, doi: https://doi.org/10.1111/pbi.13673.

[408] X. Chen *et al.*, “Generation of male-sterile soybean lines with the CRISPR/Cas9 system,” *Crop J.*, vol. 9, no. 6, pp. 1270–1277, 2021, doi: https://doi.org/10.1016/j.cj.2021.05.003.

[409] M. Nadeem *et al.*, “GmMs1 encodes a kinesin-like protein essential for male fertility in soybean (Glycine max L.),” *J. Integr. Plant Biol.*, vol. 63, no. 6, pp. 1054–1064, Jun. 2021, doi: https://doi.org/10.1111/jipb.13110.

[410] W. Wang *et al.*, “GmNMHC5 may promote nodulation via interaction with GmGAI in soybean,” *Crop J.*, vol. 10, no. 1, pp. 273–279, 2022, doi: https://doi.org/10.1016/j.cj.2021.03.019.

[411] G. Yu *et al.*, “A soybean NAC homolog contributes to resistance to Phytophthora sojae mediated by dirigent proteins,” *Crop J.*, vol. 10, no. 2, pp. 332–341, 2022, doi: https://doi.org/10.1016/j.cj.2021.08.009.

[412] S. Kumar *et al.*, “Efficient gene targeting in soybean using Ochrobactrum haywardense-mediated delivery of a marker-free donor template,” *Plant Physiol.*, vol. 189, no. 2, pp. 585–594, Jun. 2022, doi: 10.1093/plphys/kiac075.

[413] H. Zhai *et al.*, “GmMDE genes bridge the maturity gene E1 and florigens in photoperiodic regulation of flowering in soybean,” *Plant Physiol.*, vol. 189, no. 2, pp. 1021–1036, Jun. 2022, doi: 10.1093/plphys/kiac092.

[414] Z. Yang *et al.*, "A small heat shock protein, GmHSP17.9, from nodule confers symbiotic nitrogen fixation and seed yield in soybean," *Plant Biotechnol. J.*, vol. 20, no. 1, pp. 103–115, Jan. 2022, doi: https://doi.org/10.1111/pbi.13698.

[415] J. Jose and A. Sachdev, "Efficient Designing, Validation, and Transformation of GmIPK2 Specific CRISPR/Cas9 Construct for Low-Phytate Soybean TI2 - Acta Scientific Agriculture," no. doi: 10.31080/asag.2022.06.1105.

[416] R. Mu *et al.*, "GmBICs Modulate Low Blue Light-Induced Stem Elongation in Soybean ," *Frontiers in Plant Science* , vol. 13. 2022, [Online]. Available: https://www.frontiersin.org/articles/10.3389/fpls.2022.803122.

[417] Z. Zhang *et al.*, "Elimination of an unfavorable allele conferring pod shattering in an elite soybean cultivar by CRISPR/Cas9," *aBIOTECH*, vol. 3, no. 2, pp. 110–114, 2022, doi: 10.1007/s42994-022-00071-8.

[418] Y. Zhang *et al.*, "CRISPR/Cas9-Mediated Targeted Mutagenesis of GmUGT Enhanced Soybean Resistance Against Leaf-Chewing Insects Through Flavonoids Biosynthesis ," *Frontiers in Plant Science* , vol. 13. 2022, [Online]. Available: https://www.frontiersin.org/articles/10.3389/fpls.2022.802716.

[419] S. Svitashev, J. K. Young, C. Schwartz, H. Gao, S. C. Falco, and A. M. Cigan, "Targeted Mutagenesis, Precise Gene Editing, and Site-Specific Gene Insertion in Maize Using Cas9 and Guide RNA," *Plant Physiol.*, vol. 169, no. 2, pp. 931–945, Sep. 2015, doi: 10.1104/PP.15.00793.

[420] J. Zhu *et al.*, "Efficiency and Inheritance of Targeted Mutagenesis in Maize Using CRISPR-Cas9," *J. Genet. Genomics*, vol. 43, no. 1, pp. 25–36, Jan. 2016, doi: 10.1016/J.JGG.2015.10.006.

[421] C. Feng, J. Yuan, R. Wang, Y. Liu, J. A. Birchler, and F. Han, "Efficient Targeted Genome Modification in Maize Using CRISPR/Cas9 System," *J. Genet. Genomics*, vol. 43, no. 1, pp. 37–43, Jan. 2016, doi: 10.1016/J.JGG.2015.10.002.

[422] J. Shi *et al.*, "ARGOS8 variants generated by CRISPR-Cas9 improve maize grain yield under field drought stress conditions," *Plant Biotechnol. J.*, vol. 15, no. 2, pp. 207–216, Feb. 2017, doi: 10.1111/PBI.12603.

[423] Z. Liang, K. Zhang, K. Chen, and C. Gao, "Targeted Mutagenesis in Zea mays Using TALENs and the CRISPR/Cas System," *J. Genet. Genomics*, vol. 41, no. 2, pp. 63–68, Feb. 2014, doi: 10.1016/J.JGG.2013.12.001.

[424] B. Wang *et al.*, "Development of a Haploid-Inducer Mediated Genome Editing System for Accelerating Maize Breeding," *Mol. Plant*, vol. 12, no. 4, pp. 597–602, Apr. 2019, doi: 10.1016/J.MOLP.2019.03.006.

[425] X. Qi *et al.*, "Genome Editing Enables Next-Generation Hybrid Seed Production Technology," *Mol. Plant*, vol. 13, no. 9, 2020, doi: 10.1016/j.molp.2020.06.003.

[426] T. Kelliher *et al.*, "One-step genome editing of elite crop germplasm during haploid induction," *Nat. Biotechnol.*, vol. 37, no. 3, 2019, doi: 10.1038/s41587-019-0038-x.

[427] J. Li *et al.*, "A functional characterization of TaMs1 orthologs in Poaceae plants," *Crop J.*, 2021, doi: 10.1016/j.cj.2020.12.002.

[428] S. N. Char *et al.*, "An Agrobacterium-delivered CRISPR/Cas9 system for high-frequency targeted mutagenesis in maize," *Plant Biotechnol. J.*, vol. 15, no. 2, 2017, doi: 10.1111/pbi.12611.

[429] P. Barone *et al.*, “Efficient Gene Targeting in Maize Using Inducible CRISPR-Cas9 and Marker-free Donor Template,” *Mol. Plant*, vol. 13, no. 8, 2020, doi: 10.1016/j.molp.2020.06.008.

[430] K. Lee *et al.*, “Activities and specificities of CRISPR/Cas9 and Cas12a nucleases for targeted mutagenesis in maize,” *Plant Biotechnol. J.*, vol. 17, no. 2, 2019, doi: 10.1111/pbi.12982.

[431] Q. Zhang *et al.*, “A novel ternary vector system united with morphogenic genes enhances CRISPR/Cas delivery in MAIze,” *Plant Physiol.*, vol. 181, no. 4, 2019, doi: 10.1104/pp.19.00767.

[432] R. Chen *et al.*, “Generation of transgene-free maize male sterile lines using the crispr/cas9 system,” *Front. Plant Sci.*, vol. 9, 2018, doi: 10.3389/fpls.2018.01180.

[433] S. Svitashev, C. Schwartz, B. Lenderts, J. K. Young, and A. Mark Cigan, “Genome editing in maize directed by CRISPR-Cas9 ribonucleoprotein complexes,” *Nat. Commun.*, vol. 7, 2016, doi: 10.1038/ncomms13274.

[434] N. M. Doll *et al.*, “Single and multiple gene knockouts by CRISPR–Cas9 in maize,” *Plant Cell Rep.*, vol. 38, no. 4, 2019, doi: 10.1007/s00299-019-02378-1.

[435] C. Feng *et al.*, “High-efficiency genome editing using a dmc1 promoter-controlled CRISPR/Cas9 system in maize,” *Plant Biotechnol. J.*, vol. 16, no. 11, 2018, doi: 10.1111/pbi.12920.

[436] C. Gong, S. Huang, R. Song, and W. Qi, “Comparative study between the crispr/cpf1 (Cas12a) and crispr/cas9 systems for multiplex gene editing in maize,” *Agric.*, vol. 11, no. 5, 2021, doi: 10.3390/agriculture11050429.

[437] Y. Wang *et al.*, “Creation of aromatic maize by CRISPR/Cas,” *J. Integr. Plant Biol.*, vol. 63, no. 9, 2021, doi: 10.1111/jipb.13105.

[438] J. Zhang *et al.*, “Generation of Transgene-Free Semidwarf Maize Plants by Gene Editing of Gibberellin-Oxidase20-3 Using CRISPR/Cas9,” *Front. Plant Sci.*, vol. 11, 2020, doi: 10.3389/fpls.2020.01048.

[439] C. Schwartz *et al.*, “CRISPR–Cas9-mediated 75.5-Mb inversion in maize,” *Nat. Plants*, vol. 6, no. 12, 2020, doi: 10.1038/s41477-020-00817-6.

[440] Y. Yan, J. Zhu, X. Qi, B. Cheng, C. Liu, and C. Xie, “Establishment of an efficient seed fluorescence reporter-assisted CRISPR/Cas9 gene editing in maize,” *J. Integr. Plant Biol.*, vol. 63, no. 9, 2021, doi: 10.1111/jipb.13086.

[441] A. A. Malzahn *et al.*, “Application of CRISPR-Cas12a temperature sensitivity for improved genome editing in rice, maize, and Arabidopsis,” *BMC Biol.*, vol. 17, no. 1, 2019, doi: 10.1186/s12915-019-0629-5.

[442] Y. Jiang *et al.*, “CRISPR/Cas9-based discovery of maize transcription factors regulating male sterility and their functional conservation in plants,” *Plant Biotechnol. J.*, vol. 19, no. 9, 2021, doi: 10.1111/pbi.13590.

[443] C. Jing, M. Wei, P. Fang, R. Song, and W. Qi, “Pollen-specific crispr/cas9 system to increase heritable gene mutations in maize,” *Agric.*, vol. 11, no. 8, 2021, doi: 10.3390/agriculture11080751.

[444] H. J. Liu *et al.*, “High-throughput CRISPR/Cas9 mutagenesis streamlines trait gene identification in maize,” *Plant Cell*, vol. 32, no. 5, 2020, doi: 10.1105/tpc.19.00934.

[445] S. A. Bage *et al.*, "Genetic characterization of novel and CRISPR-Cas9 gene edited maize brachytic 2 alleles," *Plant Gene*, vol. 21, 2020, doi: 10.1016/j.plgene.2019.100198.

[446] W. Qi, T. Zhu, Z. Tian, C. Li, W. Zhang, and R. Song, "High-efficiency CRISPR/Cas9 multiplex gene editing using the glycine tRNA-processing system-based strategy in maize," *BMC Biotechnol.*, vol. 16, no. 1, 2016, doi: 10.1186/s12896-016-0289-2.

[447] S. Dong *et al.*, "Efficient Targeted Mutagenesis Mediated by CRISPR-Cas12a Ribonucleoprotein Complexes in Maize," *Front. Genome Ed.*, vol. 3, 2021, doi: 10.3389/fgeed.2021.670529.

[448] H. Gao *et al.*, "Superior field performance of waxy corn engineered using CRISPR–Cas9," *Nat. Biotechnol.*, vol. 38, no. 5, 2020, doi: 10.1038/s41587-020-0444-0.

[449] Y. Y. Jiang *et al.*, "Prime editing efficiently generates W542L and S621I double mutations in two ALS genes in maize," *Genome Biol.*, vol. 21, no. 1, 2020, doi: 10.1186/s13059-020-02170-5.

[450] Q. Li *et al.*, "CRISPR/Cas9-mediated knockout and overexpression studies reveal a role of maize phytochrome C in regulating flowering time and plant height," *Plant Biotechnol. J.*, vol. 18, no. 12, 2020, doi: 10.1111/pbi.13429.

[451] Z. Zhang *et al.*, "A Large Transposon Insertion in the stiff1 Promoter Increases Stalk Strength in Maize," *Plant Cell*, vol. 32, no. 1, 2020, doi: 10.1105/tpc.19.00486.

[452] D. Peterson *et al.*, "Advances in Agrobacterium transformation and vector design result in high-frequency targeted gene insertion in maize," *Plant Biotechnol. J.*, vol. 19, no. 10, 2021, doi: 10.1111/pbi.13613.

[453] Z. Li *et al.*, "Genome-wide analyses on transcription factors and their potential microRNA regulators involved in maize male fertility," *Crop J.*, 2021, doi: 10.1016/j.cj.2021.03.016.

[454] Y. Zuo, F. Feng, W. Qi, and R. Song, "Dek42 encodes an RNA-binding protein that affects alternative pre-mRNA splicing and maize kernel development," *J. Integr. Plant Biol.*, vol. 61, no. 6, 2019, doi: 10.1111/jipb.12798.

[455] C. Huang *et al.*, "ZmCCT9 enhances maize adaptation to higher latitudes," *Proc. Natl. Acad. Sci. U. S. A.*, vol. 115, no. 2, 2017, doi: 10.1073/pnas.1718058115.

[456] Q. Wu *et al.*, "The maize heterotrimeric G protein β subunit controls shoot meristem development and immune responses," *Proc. Natl. Acad. Sci. U. S. A.*, vol. 117, no. 3, 2020, doi: 10.1073/pnas.1917577116.

[457] Y. Li *et al.*, "Precise base editing of non-allelic acetolactate synthase genes confers sulfonylurea herbicide resistance in maize," *Crop J.*, vol. 8, no. 3, 2020, doi: 10.1016/j.cj.2019.10.001.

[458] M. Luo *et al.*, "Molecular dissection of maize seedling salt tolerance using a genome-wide association analysis method," *Plant Biotechnol. J.*, vol. 19, no. 10, 2021, doi: 10.1111/pbi.13607.

[459] F. Wang *et al.*, "Maize ZmPT7 regulates Pi uptake and redistribution which is modulated by phosphorylation," *Plant Biotechnol. J.*, vol. 18, no. 12, 2020, doi: 10.1111/pbi.13414.

[460] Y. Cao, X. Liang, P. Yin, M. Zhang, and C. Jiang, "A domestication-associated reduction in K + -preferring HKT transporter activity underlies maize shoot K + accumulation and salt tolerance," *New Phytol.*, vol. 222, no. 1, 2019, doi: 10.1111/nph.15605.

[461] Q. Wu, M. Regan, H. Furukawa, and D. Jackson, "Role of heterotrimeric Gα proteins in maize development and enhancement of agronomic traits," *PLoS Genet.*, vol. 14, no. 4, 2018, doi: 10.1371/journal.pgen.1007374.

[462] G. Wu *et al.*, "Characterization of maize phytochrome-interacting factors in light signaling and photomorphogenesis," *Plant Physiol.*, vol. 181, no. 2, 2019, doi: 10.1104/pp.19.00239.

[463] Z. Zhang, J. Guo, Y. Zhao, and J. Chen, "Identification and characterization of maize ACD6-like gene reveal ZmACD6 as the maize orthologue conferring resistance to Ustilago maydis," *Plant Signal. Behav.*, vol. 14, no. 10, 2019, doi: 10.1080/15592324.2019.1651604.

[464] J. S. Jaqueth *et al.*, "Fertility restoration of maize CMS-C altered by a single amino acid substitution within the Rf4 bHLH transcription factor," *Plant J.*, vol. 101, no. 1, 2020, doi: 10.1111/tpj.14521.

[465] H. Zhao *et al.*, "Loss of Function of an RNA Polymerase III Subunit Leads to Impaired Maize Kernel Development1[OPEN]," *Plant Physiol.*, vol. 184, no. 1, 2020, doi: 10.1104/PP.20.00502.

[466] L. Ma *et al.*, "Genome-wide characterization of jasmonates signaling components reveals the essential role of ZmCOI1a-ZmJAZ15 action module in regulating maize immunity to gibberella stalk rot," *Int. J. Mol. Sci.*, vol. 22, no. 2, 2021, doi: 10.3390/ijms22020870.

[467] K. Xie *et al.*, "Map-based cloning and characterization of Zea mays male sterility33 (ZmMs33) gene, encoding a glycerol-3-phosphate acyltransferase," *Theor. Appl. Genet.*, vol. 131, no. 6, 2018, doi: 10.1007/s00122-018-3083-9.

[468] J. Zhang, C. Feng, H. Su, Y. Liu, Y. Liu, and F. Han, "The cohesin complex subunit ZmSMC3 participates in meiotic centromere pairing in maize," *Plant Cell*, vol. 32, 2020, doi: 10.1105/TPC.19.00834.

[469] R. Xu *et al.*, "A C-terminal encoded peptide, ZmCEP1, is essential for kernel development in maize," *J. Exp. Bot.*, vol. 72, no. 15, 2021, doi: 10.1093/jxb/erab224.

[470] M. E. McCaw *et al.*, "Development of a Transformable Fast-Flowering Mini-Maize as a Tool for Maize Gene Editing," *Front. Genome Ed.*, vol. 2, 2021, doi: 10.3389/fgeed.2020.622227.

[471] C. Chen *et al.*, "Identification of a novel ZmSBEIIb-interacting protein involved in apparent amylose synthesis," *Can. J. Plant Sci.*, vol. 100, no. 6, 2020, doi: 10.1139/cjps-2019-0219.

[472] L. Gao *et al.*, "A kelch-repeat superfamily gene, ZmNL4, controls leaf width in maize (Zea mays L.)," *Plant J.*, vol. 107, no. 3, 2021, doi: 10.1111/tpj.15348.

[473] C. Li, Y. Yue, H. Chen, W. Qi, and R. Song, "The zmbzip22 transcription factor regulates 27-kd γ-zein gene transcription during maize endosperm development[open]," *Plant Cell*, vol. 30, no. 10, 2018, doi: 10.1105/tpc.18.00422.

[474] J. Xu *et al.*, "Seeing is believing: a visualization toolbox to enhance selection efficiency in maize genome editing," *Plant Biotechnol. J.*, vol. 19, no. 5, 2021, doi: 10.1111/pbi.13575.

[475] Y. Jiang *et al.*, "Zmfar1 and zmabcg26 regulated by microrna are essential for lipid metabolism in maize anther," *Int. J. Mol. Sci.*, vol. 22, no. 15, 2021, doi: 10.3390/ijms22157916.

[476] Q. Chen *et al.*, "Genome-wide association analyses reveal the importance of alternative splicing in diversifying gene function and regulating phenotypic variation in Maize," *Plant Cell*, vol. 30, no. 7, 2018,

doi: 10.1105/tpc.18.00109.

[477] M. Li *et al.*, “The coordinated knr6–agap–arf1 complex modulates vegetative and reproductive traits by participating in vesicle trafficking in maize,” *Cells*, vol. 10, no. 10, 2021, doi: 10.3390/cells10102601.

[478] S. Zhang *et al.*, “ZmMs25 encoding a plastid-localized fatty acyl reductase is critical for anther and pollen development in maize,” *J. Exp. Bot.*, vol. 72, no. 12, 2021, doi: 10.1093/jxb/erab142.

[479] Z. Pan *et al.*, “ZmSRL5 is involved in drought tolerance by maintaining cuticular wax structure in maize,” *J. Integr. Plant Biol.*, vol. 62, no. 12, 2020, doi: 10.1111/jipb.12982.

[480] K. M. Pathi *et al.*, “Engineering Smut Resistance in Maize by Site-Directed Mutagenesis of LIPOXYGENASE 3,” *Front. Plant Sci.*, vol. 11, 2020, doi: 10.3389/fpls.2020.543895.

[481] C. Teng, H. Zhang, R. Hammond, K. Huang, B. C. Meyers, and V. Walbot, “Dicer-like 5 deficiency confers temperature-sensitive male sterility in maize,” *bioRxiv*, 2018, doi: 10.1101/498410.

[482] E. Poretsky *et al.*, “Differential activities of maize plant elicitor peptides as mediators of immune signaling and herbivore resistance,” *Plant J.*, vol. 104, no. 6, 2020, doi: 10.1111/tpj.15022.

[483] S. N. Char *et al.*, “Heritable site-specific mutagenesis using TALENs in maize,” *Plant Biotechnol. J.*, vol. 13, no. 7, pp. 1002–1010, Sep. 2015, doi: https://doi.org/10.1111/pbi.12344.

[484] A. Kouranov *et al.*, “Demonstration of targeted crossovers in hybrid maize using CRISPR technology,” *Commun. Biol.*, vol. 5, no. 1, 2022, doi: 10.1038/s42003-022-03004-9.

[485] X. Liu *et al.*, “Use of CRISPR/Cas9-Based Gene Editing to Simultaneously Mutate Multiple Homologous Genes Required for Pollen Development and Male Fertility in Maize,” *Cells*, vol. 11, no. 3, 2022, doi: 10.3390/cells11030439.

[486] Y. Luo *et al.*, “Genetic variation in YIGE1 contributes to ear length and grain yield in maize,” *New Phytol.*, vol. 234, no. 2, 2022, doi: 10.1111/nph.17882.

[487] C. Liu *et al.*, “Targeted generation of Null Mutants in ZmGDIα confers resistance against maize rough dwarf disease without agronomic penalty,” *Plant Biotechnol. J.*, vol. 20, no. 5, 2022, doi: 10.1111/pbi.13793.

[488] H. Guan *et al.*, “Genetic Variation in ZmPAT7 Contributes to Tassel Branch Number in Maize,” *Int. J. Mol. Sci.*, vol. 23, no. 5, 2022, doi: 10.3390/ijms23052586.

[489] W. Sun *et al.*, “Maize Interveinal Chlorosis 1 links the Yang Cycle and Fe homeostasis through Nicotianamine biosynthesis,” *Plant Physiol.*, vol. 188, no. 4, 2022, doi: 10.1093/plphys/kiac009.

[490] X. Qi *et al.*, “ZmCOI2a and ZmCOI2b redundantly regulate anther dehiscence and gametophytic male fertility in maize,” *Plant J.*, vol. 110, no. 3, 2022, doi: 10.1111/tpj.15708.

[491] L. Liu *et al.*, “Enhancing grain-yield-related traits by CRISPR–Cas9 promoter editing of maize CLE genes,” *Nat. Plants*, pp. 1–8, Feb. 2021, doi: 10.1038/s41477-021-00858-5.

[492] J. Zhang *et al.*, “The SUMO ligase MMS21 profoundly influences maize development through its impact on genome activity and stability,” *PLoS Genet.*, vol. 17, no. 10, 2021, doi: 10.1371/journal.pgen.1009830.

[493] Y. Li *et al.*, “Loss-of-function alleles of ZmPLD3 cause haploid induction in maize,” *Nat. Plants*, vol. 7, no. 12, 2021, doi: 10.1038/s41477-021-01037-2.

[494] P. Li, Z. Li, G. Xie, and J. Zhang, “Trihelix transcription factor zmthx20 is required for kernel development in maize,” *Int. J. Mol. Sci.*, vol. 22, no. 22, 2021, doi: 10.3390/ijms222212137.

[495] P. Gong *et al.*, “SAMBA controls cell division rate during maize development,” *Plant Physiol.*, vol. 188, no. 1, pp. 411–424, Jan. 2022, doi: 10.1093/PLPHYS/KIAB514.

[496] R. S. Yang *et al.*, “Glutaredoxins regulate maize inflorescence meristem development via redox control of TGA transcriptional activity,” *Nat. Plants*, vol. 7, no. 12, 2021, doi: 10.1038/s41477-021-01029-2.

[497] S. Wang, S. Zhang, W. Wang, X. Xiong, F. Meng, and X. Cui, “Efficient targeted mutagenesis in potato by the CRISPR/Cas9 system,” *Plant Cell Rep.*, vol. 34, no. 9, pp. 1473–1476, 2015, doi: 10.1007/s00299-015-1816-7.

[498] W. J. Stiekema, F. Heidekamp, J. D. Louwerse, H. A. Verhoeven, and P. Dijkhuis, “Introduction of foreign genes into potato cultivars Bintje and Désirée using an Agrobacterium tumefaciens binary vector,” *Plant Cell Rep.*, vol. 7, no. 1, pp. 47–50, 1988, doi: 10.1007/BF00272976.

[499] N. M. Butler, P. A. Atkins, D. F. Voytas, and D. S. Douches, “Generation and Inheritance of Targeted Mutations in Potato (Solanum tuberosum L.) Using the CRISPR/Cas System,” *PLoS One*, vol. 10, no. 12, p. e0144591, Dec. 2015, [Online]. Available: https://doi.org/10.1371/journal.pone.0144591.

[500] B. M. Clasen *et al.*, “Improving cold storage and processing traits in potato through targeted gene knockout,” *Plant Biotechnol. J.*, vol. 14, no. 1, pp. 169–176, Jan. 2016, doi: https://doi.org/10.1111/pbi.12370.

[501] A. Forsyth, T. Weeks, C. Richael, and H. Duan, “Transcription Activator-Like Effector Nucleases (TALEN)-Mediated Targeted DNA Insertion in Potato Plants ,” *Frontiers in Plant Science* , vol. 7. 2016, [Online]. Available: https://www.frontiersin.org/articles/10.3389/fpls.2016.01572.

[502] N. M. Butler, N. J. Baltes, D. F. Voytas, and D. S. Douches, “Geminivirus-Mediated Genome Editing in Potato (Solanum tuberosum L.) Using Sequence-Specific Nucleases ,” *Frontiers in Plant Science* , vol. 7. 2016, [Online]. Available: https://www.frontiersin.org/articles/10.3389/fpls.2016.01045.

[503] A. Nicolia *et al.*, “Targeted gene mutation in tetraploid potato through transient TALEN expression in protoplasts,” *J. Biotechnol.*, vol. 204, pp. 17–24, 2015, doi: https://doi.org/10.1016/j.jbiotec.2015.03.021.

[504] S. Sawai *et al.*, “Sterol Side Chain Reductase 2 Is a Key Enzyme in the Biosynthesis of Cholesterol, the Common Precursor of Toxic Steroidal Glycoalkaloids in Potato ,” *Plant Cell*, vol. 26, no. 9, pp. 3763–3774, Sep. 2014, doi: 10.1105/tpc.114.130096.

[505] M. Andersson, H. Turesson, A. Nicolia, A.-S. Fält, M. Samuelsson, and P. Hofvander, “Efficient targeted multiallelic mutagenesis in tetraploid potato (Solanum tuberosum) by transient CRISPR-Cas9 expression in protoplasts,” *Plant Cell Rep.*, vol. 36, no. 1, pp. 117–128, 2017, doi: 10.1007/s00299-016-2062-3.

[506] X. Zhou, M. Zha, J. Huang, L. Li, M. Imran, and C. Zhang, “StMYB44 negatively regulates phosphate transport by suppressing expression of PHOSPHATE1 in potato,” *J. Exp. Bot.*, vol. 68, no. 5, pp. 1265–1281, Feb. 2017, doi: 10.1093/jxb/erx026.

[507] M. Andersson *et al.*, “Genome editing in potato via CRISPR-Cas9 ribonucleoprotein delivery,” *Physiol. Plant.*, vol. 164, no. 4, pp. 378–384, Dec. 2018, doi: https://doi.org/10.1111/ppl.12731.

[508] H. Kusano *et al.*, “Establishment of a modified CRISPR/Cas9 system with increased mutagenesis frequency using the translational enhancer dMac3 and multiple guide RNAs in potato,” *Sci. Rep.*, vol. 8, no. 1, p. 13753, 2018, doi: 10.1038/s41598-018-32049-2.

[509] M. Nakayasu *et al.*, “Generation of α-solanine-free hairy roots of potato by CRISPR/Cas9 mediated genome editing of the St16DOX gene,” *Plant Physiol. Biochem.*, vol. 131, pp. 70–77, 2018, doi: https://doi.org/10.1016/j.plaphy.2018.04.026.

[510] M. Ye *et al.*, “Generation of self-compatible diploid potato by knockout of S-RNase,” *Nat. Plants*, vol. 4, no. 9, pp. 651–654, 2018, doi: 10.1038/s41477-018-0218-6.

[511] F. Veillet *et al.*, “Transgene-Free Genome Editing in Tomato and Potato Plants Using Agrobacterium-Mediated Delivery of a CRISPR/Cas9 Cytidine Base Editor,” *International Journal of Molecular Sciences*, vol. 20, no. 2. 2019, doi: 10.3390/ijms20020402.

[512] S. Yasumoto *et al.*, “Efficient genome engineering using Platinum TALEN in potato,” *Plant Biotechnol.*, vol. 36, no. 3, pp. 167–173, 2019, doi: 10.5511/plantbiotechnology.19.0805a.

[513] J. Ma, “Targeted Editing of the StPDS Gene using the CRISPR/Cas9 system in Tetraploid Potato,” *Emirates J. Food Agric.*, vol. 31, no. 7 SE-Research Article, Aug. 2019, doi: https://doi.org/10.9755/ejfa.2019.v31.i7.1974.

[514] F. Enciso-Rodriguez, N. C. Manrique-Carpintero, S. S. Nadakuduti, C. R. Buell, D. Zarka, and D. Douches, “Overcoming Self-Incompatibility in Diploid Potato Using CRISPR-Cas9 ,” *Frontiers in Plant Science* , vol. 10. 2019, [Online]. Available: https://www.frontiersin.org/articles/10.3389/fpls.2019.00376.

[515] I. E. Johansen *et al.*, “High efficacy full allelic CRISPR/Cas9 gene editing in tetraploid potato,” *Sci. Rep.*, vol. 9, no. 1, p. 17715, 2019, doi: 10.1038/s41598-019-54126-w.

[516] A. Tuncel *et al.*, “Cas9-mediated mutagenesis of potato starch-branching enzymes generates a range of tuber starch phenotypes,” *Plant Biotechnol. J.*, vol. 17, no. 12, pp. 2259–2271, Dec. 2019, doi: https://doi.org/10.1111/pbi.13137.

[517] F. Veillet *et al.*, “The Solanum tuberosum GBSSI gene: a target for assessing gene and base editing in tetraploid potato,” *Plant Cell Rep.*, vol. 38, no. 9, pp. 1065–1080, 2019, doi: 10.1007/s00299-019-02426-w.

[518] N. M. Butler, S. H. Jansky, and J. Jiang, “First-generation genome editing in potato using hairy root transformation,” *Plant Biotechnol. J.*, vol. 18, no. 11, pp. 2201–2209, Nov. 2020, doi: https://doi.org/10.1111/pbi.13376.

[519] S. Yasumoto *et al.*, “Targeted genome editing in tetraploid potato through transient TALEN expression by *Agrobacterium* infection,” *Plant Biotechnol.*, vol. 37, no. 2, pp. 205–211, 2020, doi: 10.5511/plantbiotechnology.20.0525a.

[520] M. N. González *et al.*, “Reduced Enzymatic Browning in Potato Tubers by Specific Editing of a Polyphenol Oxidase Gene via Ribonucleoprotein Complexes Delivery of the CRISPR/Cas9 System ,” *Frontiers in Plant Science* , vol. 10. 2020, [Online]. Available:

https://www.frontiersin.org/articles/10.3389/fpls.2019.01649.

[521] F. Sevestre, M. Facon, F. Wattebled, and N. Szydlowski, “Facilitating gene editing in potato: a Single-Nucleotide Polymorphism (SNP) map of the Solanum tuberosum L. cv. Desiree genome,” *Sci. Rep.*, vol. 10, no. 1, p. 2045, 2020, doi: 10.1038/s41598-020-58985-6.

[522] F. Veillet, M.-P. Kermarrec, L. Chauvin, J.-E. Chauvin, and F. Nogué, “CRISPR-induced indels and base editing using the Staphylococcus aureus Cas9 in potato,” *PLoS One*, vol. 15, no. 8, p. e0235942, Aug. 2020, [Online]. Available: https://doi.org/10.1371/journal.pone.0235942.

[523] F. Veillet *et al.*, “Expanding the CRISPR Toolbox in P. patens Using SpCas9-NG Variant and Application for Gene and Base Editing in Solanaceae Crops,” *International Journal of Molecular Sciences*, vol. 21, no. 3. 2020, doi: 10.3390/ijms21031024.

[524] Z. Bánfalvi, E. Csákvári, V. Villányi, and M. Kondrák, “Generation of transgene-free PDS mutants in potato by Agrobacterium-mediated transformation,” *BMC Biotechnol.*, vol. 20, no. 1, p. 25, 2020, doi: 10.1186/s12896-020-00621-2.

[525] A. V Khromov, A. V Makhotenko, S. S. Makarova, T. P. Suprunova, N. O. Kalinina, and M. E. Taliansky, “Delivery of CRISPR/Cas9 Ribonucleoprotein Complex into Plant Apical Meristem Cells Leads to Large Deletions in an Editing Gene,” *Russ. J. Bioorganic Chem.*, vol. 46, no. 6, pp. 1242–1249, 2020, doi: 10.1134/S1068162020060138.

[526] M. N. González *et al.*, “Comparative potato genome editing: Agrobacterium tumefaciens-mediated transformation and protoplasts transfection delivery of CRISPR/Cas9 components directed to StPPO2 gene,” *Plant Cell, Tissue Organ Cult.*, vol. 145, no. 2, pp. 291–305, 2021, doi: 10.1007/s11240-020-02008-9.

[527] N. P. Kieu, M. Lenman, E. S. Wang, B. L. Petersen, and E. Andreasson, “Mutations introduced in susceptibility genes through CRISPR/Cas9 genome editing confer increased late blight resistance in potatoes,” *Sci. Rep.*, vol. 11, no. 1, p. 4487, 2021, doi: 10.1038/s41598-021-83972-w.

[528] Z. Zheng, G. Ye, Y. Zhou, X. Pu, W. Su, and J. Wang, “Editing sterol side chain reductase 2 gene (StSSR2) via CRISPR/Cas9 reduces the total steroidal glycoalkaloids in potato,” *All Life*, vol. 14, no. 1, pp. 401–413, Jan. 2021, doi: 10.1080/26895293.2021.1925358.

[529] A. Takeuchi *et al.*, “Creation of a potato mutant lacking the starch branching enzyme gene *StSBE3* that was generated by genome editing using the CRISPR/dMac3-Cas9 system,” *Plant Biotechnol.*, vol. 38, no. 3, pp. 345–353, 2021, doi: 10.5511/plantbiotechnology.21.0727a.

[530] X. Zhao *et al.*, “Amylose starch with no detectable branching developed through DNA-free CRISPR-Cas9 mediated mutagenesis of two starch branching enzymes in potato,” *Sci. Rep.*, vol. 11, no. 1, p. 4311, 2021, doi: 10.1038/s41598-021-83462-z.

[531] G. Acha *et al.*, “A Traceable DNA-Replicon Derived Vector to Speed Up Gene Editing in Potato: Interrupting Genes Related to Undesirable Postharvest Tuber Traits as an Example,” *Plants*, vol. 10, no. 9. 2021, doi: 10.3390/plants10091882.

[532] L. Ramírez Gonzales *et al.*, “Potato CYCLING DOF FACTOR 1 and its lncRNA counterpart StFLORE link tuber development and drought response,” *Plant J.*, vol. 105, no. 4, pp. 855–869, Feb. 2021, doi: https://doi.org/10.1111/tpj.15093.

[533] N. Hegde, S. Joshi, N. Soni, and A. C. Kushalappa, “The caffeoyl-CoA O-methyltransferase gene SNP replacement in Russet Burbank potato variety enhances late blight resistance through cell wall reinforcement,” *Plant Cell Rep.*, vol. 40, no. 1, pp. 237–254, 2021, doi: 10.1007/s00299-020-02629-6.

[534] B. B. Salam *et al.*, “Sucrose promotes stem branching through cytokinin,” *Plant Physiol.*, vol. 185, no. 4, pp. 1708–1721, Apr. 2021, doi: 10.1093/plphys/kiab003.

[535] J. Odipio, T. Alicai, I. Ingelbrecht, D. A. Nusinow, R. Bart, and N. J. Taylor, “Efficient CRISPR/Cas9 Genome Editing of Phytoene desaturase in Cassava ,” *Frontiers in Plant Science* , vol. 8. 2017, [Online]. Available: https://www.frontiersin.org/article/10.3389/fpls.2017.01780.

[536] B. S. E. *et al.*, “Accelerated ex situ breeding of GBSS- and PTST1-edited cassava for modified starch,” *Sci. Adv.*, vol. 4, no. 9, p. eaat6086, Apr. 2022, doi: 10.1126/sciadv.aat6086.

[537] M. A. Gomez *et al.*, “Simultaneous CRISPR/Cas9-mediated editing of cassava eIF4E isoforms nCBP-1 and nCBP-2 reduces cassava brown streak disease symptom severity and incidence,” *Plant Biotechnol. J.*, vol. 17, no. 2, pp. 421–434, Feb. 2019, doi: https://doi.org/10.1111/pbi.12987.

[538] K. M. Veley *et al.*, “Gene tagging via CRISPR-mediated homology-directed repair in cassava,” *G3 Genes|Genomes|Genetics*, vol. 11, no. 4, p. jkab028, Apr. 2021, doi: 10.1093/g3journal/jkab028.

[539] A. W. Hummel *et al.*, “Allele exchange at the EPSPS locus confers glyphosate tolerance in cassava,” *Plant Biotechnol. J.*, vol. 16, no. 7, pp. 1275–1282, Jul. 2018, doi: https://doi.org/10.1111/pbi.12868.

[540] S. Luo *et al.*, “Editing of the starch branching enzyme gene SBE2 generates high-amylose storage roots in cassava,” *Plant Mol. Biol.*, vol. 108, no. 4, pp. 429–442, 2022, doi: 10.1007/s11103-021-01215-y.

[541] W. Zhang *et al.*, “Identification of no pollen 1 provides a candidate gene for heterosis utilization in foxtail millet (Setaria italica L.),” *Crop J.*, vol. 9, no. 6, pp. 1309–1319, 2021, doi: https://doi.org/10.1016/j.cj.2021.03.018.

[542] S. Mamidi *et al.*, “A genome resource for green millet Setaria viridis enables discovery of agronomically valuable loci,” *Nat. Biotechnol.*, vol. 38, no. 10, pp. 1203–1210, 2020, doi: 10.1038/s41587-020-0681-2.

[543] Z. Liang, Y. Wu, L. Ma, Y. Guo, and Y. Ran, “Efficient Genome Editing in Setaria italica Using CRISPR/Cas9 and Base Editors ,” *Frontiers in Plant Science* , vol. 12. 2022, [Online]. Available: https://www.frontiersin.org/articles/10.3389/fpls.2021.815946.
